# Revealing drivers and risks for power grid frequency stability with explainable AI

**DOI:** 10.1016/j.patter.2021.100365

**Published:** 2021-10-08

**Authors:** Johannes Kruse, Benjamin Schäfer, Dirk Witthaut

**Affiliations:** 1 Forschungszentrum Jülich, Institute of Energy and Climate Research - Systems Analysis and Technology Evaluation (IEK-STE), 52425 Jülich, Germany; 2 Institute for Theoretical Physics, University of Cologne, 50937 Köln, Germany; 3 School of Mathematical Sciences, Queen Mary University of London, London E1 4NS, UK; 4 Faculty of Science and Technology, Norwegian University of Life Sciences, 1432 Ås, Norway

**Keywords:** power grid, frequency, RoCoF, nadir, stability, power system, explanations, explainable artificial intelligence, machine learning

## Abstract

Stable operation of an electric power system requires strict operational limits for the grid frequency. Fluctuations and external impacts can cause large frequency deviations and increased control efforts. Although these complex interdependencies can be modeled using machine learning algorithms, the black box character of many models limits insights and applicability. In this article, we introduce an explainable machine learning model that accurately predicts frequency stability indicators for three European synchronous areas. Using Shapley additive explanations, we identify key features and risk factors for frequency stability. We show how load and generation ramps determine frequency gradients, and we identify three classes of generation technologies with converse impacts. Control efforts vary strongly depending on the grid and time of day and are driven by ramps as well as electricity prices. Notably, renewable power generation is central only in the British grid, while forecasting errors play a major role in the Nordic grid.

## Highlights

• Power grid frequency stability is analyzed via explainable artificial intelligence
• Effect of generation ramps differ between Continental Europe, Britain, Nordic grids
• Control efforts are driven by electricity prices and load ramps
• Renewable generation and forecasting errors dominate Britain and Nordic grids

## The bigger picture

The transition to a sustainable energy system is challenging for the operation and stability of electric power systems as power generation becomes increasingly uncertain, grid loads increase, and their dynamical properties fundamentally change. At the same time, operational data are available at an unprecedented level of detail, enabling new methods of monitoring and control. To fully harness these data, advanced methods from machine learning must be used.

In this paper, we present explainable artificial intelligence (XAI) as a tool to quantify, predict, and explain essential aspects of power system operation and stability in three major European synchronous areas. We focus on the power grid frequency, which measures the balance of generation and load and thus provides the central observable for control and balancing. Combining XAI with domain knowledge, we identify the main drivers and stability risks, while our model and open dataset may enable further XAI research on power systems.

## Teaser

The transition to a sustainable energy system is one of the greatest challenges of our time. With operational data becoming available at an unprecedented level of detail, advanced methods from machine learning must be used to fully harness these data.In our article, we present explainable artificial intelligence (XAI) as a tool to quantify, predict, and explain essential aspects of power system operation and stability in three major European areas, giving indications of how to stably operate future power systems.

## Introduction

The power grid frequency plays a central role for power system control, as it reflects the balance of power generation and demand.^1^ An oversupply of power leads to a frequency increase, while a shortage causes a frequency decrease. Large frequency deviations correspond to large power imbalances, which threaten system stability and may lead to large-scale blackouts.^2^ Frequency stability is regarded as a major challenge for the transition to a sustainable energy system because renewable power sources do not provide an intrinsic inertia.^3^ Understanding the emergence of large frequency deviations is therefore essential.

Deviations from the reference frequency of 50/60 Hz have distinct causes, which are in turn modified by the complex interplay of different elements of the energy system. For example, changes in power generation due to electricity trading intervals causes regular frequency jumps,^4^ the magnitude of which depends on several technical parameters.^3^^,^^5^ Fluctuating wind and solar power^6^^,^^7^ or singular load patterns due to societal events^8^ create frequency fluctuations on different scales. To guarantee frequency stability in such a complex and uncertain environment, transmission system operators (TSOs) intensively monitor the system and allocate expensive control reserves as necessary. An improved understanding of the frequency dynamics and external influences could greatly facilitate control efforts and contribute to power system stability. While several studies have investigated univariate correlations to quantify the impact of individual features,^9^, ^10^, ^11^ a comprehensive, data-based analysis is lacking.

Modern machine learning (ML) methods are well suited to this task as they can handle a large number of features and large volumes of data. In recent years, the volume of publicly available energy system data has grown steadily, including frequency recordings^12^^,^^13^ and data on a variety of external features, such as generation and load time series.^14^^,^^15^ An optimal basis for analyzing and predicting grid frequency with data-driven models therefore already exists.^16^ However, complex ML models do not provide insights on the mapping of input to output.^17^^,^^18^ This is particularly problematic for critical infrastructures such as power systems, where the black box character poses a security risk.^19^^,^^20^

Approaches using explainable artificial intelligence (XAI) could change this. XAI is a quickly growing research field, which covers inherently transparent ML models as well as post-modeling explanations for black box models.^21^ Shapley additive explanations (SHAP) values are an example of post-modeling explanations, offering a method of measuring feature effects and avoiding inconsistencies present in other approaches.^22^^,^^23^ In particular, SHAP values have certain desirable properties, such as additivity, efficiency, and symmetry. SHAP values can be quickly computed for gradient boosted trees,^24^ which in turn offer a powerful nonlinear modeling and are particularly suited to tabular data. The combination of tree-based models and SHAP values is already widely used, with applications ranging from medicine^25^ to geoscience.^26^ In contrast, only a few applications of this methodology have been presented in the field of energy systems analysis to date: for example, to explain solar power forecasts,^27^ transient security assessments,^28^ or power project failures.^29^

Here, we present an explainable ML model based on gradient boosted trees for selected indicators of frequency stability, and we evaluate its predictive power for three grids in Europe: Continental Europe (CE), the Nordic area, and Great Britain (GB). We demonstrate the benefits of explainability via SHAP values, ranging from coarse-grained global feature importances to detailed dependencies and finally to fine-grained interactions between different external features. In particular, we quantify the impacts of generation and load ramps on the rate of change of frequency (RoCoF) at the beginning of each hour. SHAP values explain the different impacts and roles of different generation technologies. We use aggregated SHAP values to analyze efforts to control generation, which vary strongly depending on the grid and time of the day. We then investigate enduring frequency deviations, which can be attributed to systemic power imbalances, and discuss the role of solar power generation. As data, we utilize the hourly time series of four stability indicators (model outputs or targets) and 66 external features (model inputs) for the years 2015–2019 (see also our Zenodo^30^ repository).

Our approach complements established simulation-based methods that predict frequency deviations on the basis of load and generation forecasts. Although simulations can be very accurate, they are reliant on the quality of input data, underlying forecasts, and specific parameters. Data-driven models can reveal additional driving factors, unknown effects, and emerging risks and thus complement and improve existing simulations. For instance, our analysis highlights the role of forecasting errors, which varies depending on the grid.

The next two sections of this paper present the four frequency stability indicators and our ML model. Then, the most important features in each synchronous area are identified before the influence on generator ramps—in particular, on RoCoF predictions—are discussed and nonlinear feature dependencies are revealed. We go on to demonstrate how SHAP analysis reveals feature interactions before concluding with a discussion.

## Results

### Frequency stability: Indicators and influences

**Figure 1:**
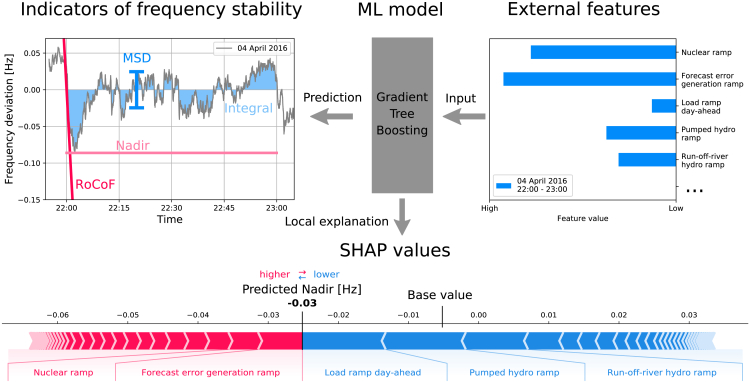
Overview of our explainable ML model From right to left: using publicly available external features from the ENTSO-E transparency platform,^36^ such as load ramps or generation ramps, a gradient tree boosting ML model was constructed to predict indicators of frequency stability. The model was then interpreted using SHAP values, which quantify the effect of the features on the model output in relation to a base value (see experimental procedures). The SHAP value of each feature is shown at the bottom of the figure, separated by white arrows; only the most important features are named. Together with the base value, positive (red) and negative (blue) SHAP values add up to the model prediction. The data represent a sample hour in 2016 from Continental Europe.

The power grid frequency fluctuates on various timescales, ranging from seconds to weeks.^31^ In our model, we aggregated frequency deviations to hourly indicators, which are directly relevant for power system stability (Figure 1; experimental procedures). We analyzed the maximum frequency deviation within the hour (nadir)^32^ and the RoCoF,^32^ which are of central relevance for grid monitoring and control. Nadirs above a threshold level indicate immediate danger and can be counteracted with measures such as load shedding. High RoCoFs are dangerous because control actions require a few seconds to take effect. In addition, we evaluated two integrated stability measures to account for the duration of frequency deviations. We characterized the variability of hourly time series using the mean square deviation (MSD) from 50 Hz. The MSD also indicates the total (primary) control effort, meaning that a large MSD reflects high operational costs.^33^ Finally, we evaluated the integrated frequency deviation (integral), which is proportional to the mean deviation within the hour. Large integrals correspond to a systematic imbalance between the hourly power generation and the demand. Regional differences in the grid frequency within a synchronous area are small during normal operation and are typically damped out after several seconds.^34^^,^^35^ Although we used local grid frequency measurements, the above indicators characterize frequency stability in an entire synchronous area.

We evaluated these four indicators on an hourly basis, as this timescale is central for power system operation.^37^ Electricity is traded predominantly in blocks of one hour, and generation is adapted at the beginning of each hour, leading to deterministic patterns in frequency.^4^ When the load decreases continuously during an hour, but the dispatch is set to the hourly mean, then power is scarce at the beginning of the hour and the frequency drops. As a consequence, frequency deviations show a pronounced daily profile, which we use later as a null model to evaluate prediction performance. Another reason for choosing to evaluate on an hourly basis is that most external features are only publicly available at an hourly resolution.^14^

The assessment of frequency stability indicators conventionally focuses on the transient response after a major disturbance.^1^ Many model-based simulation studies have investigated the effects of various parameters on the frequency response, in particular the effect of inertia,^38^ as well as effects on the properties of the load-frequency control system.^10^ In recent years, ambient and deterministic frequency fluctuations have received more attention in the context of model-based simulations. Studies have highlighted the influence of inertia, control system parameters,^5^ and intermittent wind power feed-in^39^ on the frequency statistics. Deterministic frequency deviations (DFDs) have been studied using dynamical models^4^ and stochastic models^40^ revealing the importance of the daily load evolution and generation jumps caused by electricity trading. The main limitation of the simulation approach is that data and parameters are often not publicly available to accurately model all interactions within the power system. For example, load-frequency control systems are operated by individual TSOs, and parameters may have been disclosed to other TSOs.^41^ Thus, simplified assumptions are used, which often do not reflect effects present in real-world data.

Over the last few years, comprehensive datasets have become publicly available, enabling an *empirical analysis* of power system frequency stability.^13^^,^^35^ Most data-driven studies focus on the impact of a single isolated feature and resort to a linear correlation analysis. For instance, studies have quantified the correlations between different measures of frequency quality and the load value and ramps in the Nordic grid,^11^ wind power generation in the Irish grid,^9^ load ramps in the British grid,^10^ and societal events coinciding with large frequency deviations.^8^ A correlation between load and solar ramps as well as trading volumes reflects the role of solar power in power balancing.^42^ The relation between wind power generation and large frequency increments in the CE grid has been studied using conditional probabilities by Haehne et al.^7^ Although existing studies provide us with essential insights into power system operation and frequency stability, they are limited in two ways. Firstly, linear correlation analyses cannot capture nonlinear dependencies and may thus underestimate or even overlook important effects. Secondly, only one feature/covariate is used in most cases, and the observed effects are not adjusted for other variables. This is problematic when features are correlated, e.g. due to confounding. Modern ML methods can capture multiple dependencies and thus provide more accurate results.^16^

### An explainable model for frequency deviations

We developed an explainable ML model to predict indicators of frequency stability from external features (Figure 1; experimental procedures). We used gradient tree boosting (GTB), which produces nonlinear models with state-of-the-art performance for many ML applications^43^ while enabling a fast and efficient calculation of SHAP values to explain the predictions.^24^ We fed our model with physically meaningful features based on load, generation, and electricity price time series. Our data included both day-ahead available features, such as the day-ahead predicted load change (“load ramp day-ahead”) and ex post available features, such as the error between the day-ahead forecast and the actual total generation change (“forecast error generation ramp”). Finally, we computed SHAP values to quantify how each feature contributes to the model output. For example, in Figure 1 (bottom), the feature “load ramp day-ahead” has a negative contribution (blue), thus causing the predicted nadir to be lower than its average. SHAP values make local predictions more transparent and enable aggregated insights into global feature effects, dependencies, and interactions. However, it should be noted that SHAP values do not guarantee causal relations (see experimental procedures for a more detailed discussion).

Based on its $R^{2}$ score, our model outperformed the daily average profile of the stability indicators, which is an important system-specific null model (experimental procedures and supplemental experimental procedures S5). We achieved performances 3.7 (CE), 7.6 (Nordic), and 16.3 (GB) times higher than the daily profile, thus indicating additional important dependencies. Restricting the full model to day-ahead available features resulted in similar performance gains of 2.6 (CE), 3.0 (Nordic), and 8.9 (GB), which opens the possibility of predicting stability indicators a day ahead. The ability to include ex post available features, such as forecast errors, was particularly beneficial in the Nordic area. Here, the full model performed 2.6 times better than the restricted day-ahead model. This indicates the importance of forecast errors for the Nordic frequency dynamics, which we examine in the next section.

### Main features affecting frequency deviations

**Figure 2:**
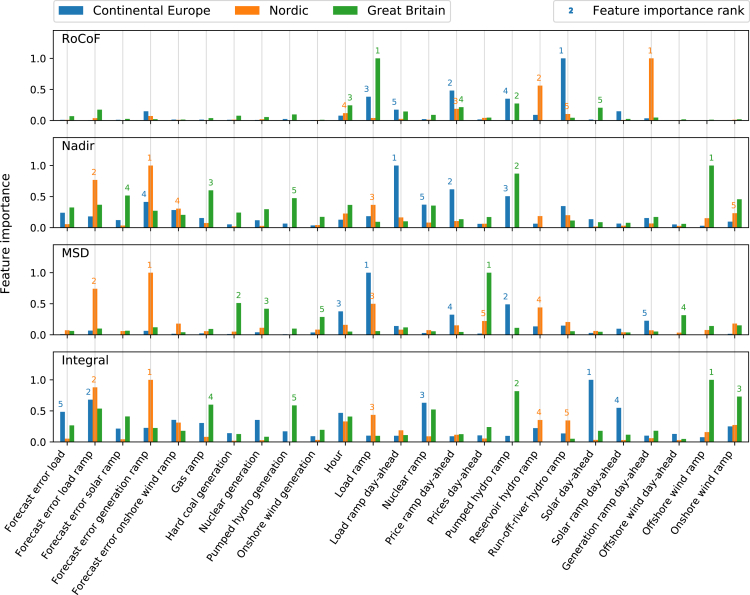
Most important features for predicting frequency stability The feature importance in our model is measured by the mean absolute SHAP value. The union of the five most important features for each stability indicator and area is shown (see experimental procedures). The importance rank of the five most important features for each area is given above the corresponding bar. While forecast errors and load and generation ramps have a high relative importance, the total synchronous generation is not among the five most important features and its average effect is therefore negligible.

We demonstrated our model explainability on the coarse-grained level of global feature importances, which characterize how much a certain feature affects the hourly frequency stability indicators within the trained model (Figure 2).

In the RoCoF model, only a few features dominated: mainly generation ramps from hydropower and load ramps. The importance of hydropower generation ramps relates to their large ramping speed, which we discuss below. In the Nordic area, the total day-ahead generation ramp is much more important than load ramps for the RoCoF. This suggests that changes in power export and storage may be relevant here, as these are not represented in the load for the area.

The nadir was primarily affected by ramps and their respective forecasting errors. In CE, the day-ahead load ramp was the most important feature. This reflects the importance of DFDs, which are strongly correlated to the direction of the load ramps.^4^ In the Nordic grid, the forecast errors of generation and load ramp were by far the most important features, partly explaining the large performance gain when ex post data were included in the model (see above). In contrast, there were many features of almost equal importance in the British nadir model. Here, wind power ramps and solar ramp forecast errors were among the five most important features. This indicates the importance of renewables for frequency deviations in GB.

The MSD behaved similarly to the nadir in CE and in the Nordic grid, with some subtle differences. Load ramps were the most important feature in CE. Forecasting errors again dominated in the Nordic grid, but load and hydropower generation ramps also played a role. A different situation was found in GB. Day-ahead prices dominated the MSD prediction, with some generation forms (coal and nuclear) coming in at a distant second, while generation ramps did not significantly contribute. These differences point to a more complex behavior of the MSD, which we further discuss below.

Finally, the integral was largely affected by forecasting errors for load and generation ramps, which caused long-lasting power mismatches. This was particularly evident in the Nordic grid, where other features were not as important. In GB, wind power ramps were ranked highly, confirming the importance of renewables. In CE, solar power generation and ramps, as well as nuclear power ramps, were relevant for the prediction. We investigated how the interaction of these two distinct generation types explain model variance.

In summary, CE exhibited strong DFDs that were connected to hourly load and generation ramps. This is consistent with previous results^4^^,^^41^ (Figure S10). Nordic frequency deviations were strongly connected to forecasting errors, which is in line with Nordic TSOs reporting forecast errors as a driver.^44^ In GB, hourly DFDs were less important (supplemental experimental procedures S4) and frequency deviations were mainly affected by renewables, i.e. their fluctuations and forecast errors (cf. Nationalgrid ESO^45^). The total synchronous generation, which approximates the inertia in our model (experimental procedures), affected the British frequency dynamics only in extreme situations where there was very low inertia (supplemental experimental procedures S6). Despite the importance of reduced inertia in renewable energy systems,^3^^,^^45^ our model showed that the average effect of inertia on the aggregated stability indicators is negligible (Figure 2). This was consistent with other studies on aggregated frequency fluctuations (cf. Vorobev et al.^5^), which found that inertia is important for extreme events but aggregated dynamics are not. It should be noted that we focused on frequent daily fluctuations and stability concerns, which are highly relevant for TSOs and for reducing daily operational costs.^33^ This supplements studies focusing on blackouts and cascading failures.^46^

### Characterizing the effect of generation ramps

**Figure 3:**
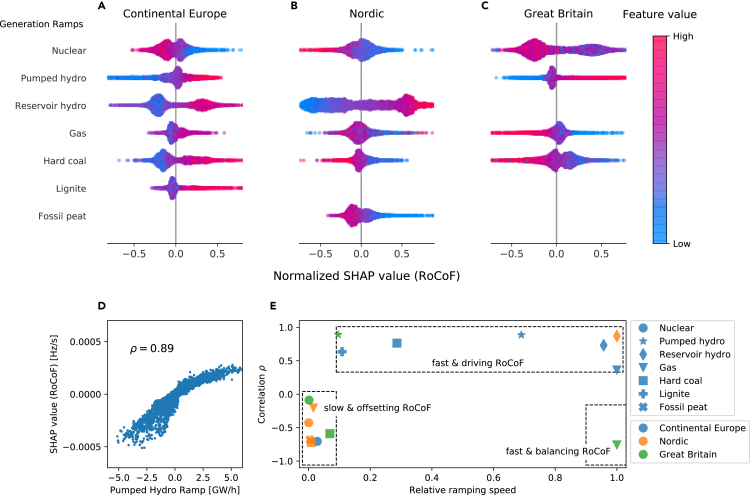
Effect of generation ramps on the RoCoF (A–C) Examination of the effects of dispatchable, i.e., weather-independent, generation technologies, which generally affect the hourly RoCoF due to their gradual change at the beginning of (hourly) trading intervals.^4^ The bee swarm chart depicts the SHAP effects on the RoCoF in the Continental Europe (A), the Nordic (B), and the Great Britain (C) grid areas. For each area and generation type, we normalized the SHAP values by their maximum absolute value to improve visibility. Each colored dot represents one time step in the dataset and the dots pile up vertically to indicate their density on the x axis. The figure only examines generation ramps with a feature importance higher than 0.01 to ensure reliability of results. (D) Quantification of the direction of the dependencies with the correlation between the feature value and the SHAP effect, shown here for pumped hydro ramps. (E) Combining the directions with the relative ramping speeds of the generation technologies (see experimental procedures) helps to distinguish RoCoF-driving and RoCoF-offsetting technologies within the three areas.

Fast generation ramps significantly affect the hourly RoCoF. For this reason, we went beyond mean feature importances and examined the direction of these dependencies using SHAP values (Figure 3). The effect of ramps is mostly monotonic, meaning that a feature effect either increases or decreases monotonically with the feature value (Figures 3A–3C). Remarkably, the direction of the dependency varies depending on the type of generation and the grid. As expected, hydropower generation ramps were consistently positively correlated (see Figure 3D for CE). The dependency of hard coal ramps for CE was the opposite to the dependency for GB and the Nordic grid.

The observed differences between the generation types can be explained in terms of the *relative* ramping speed of a generation type within a respective area (see experimental procedures on how this speed is estimated). In the Nordic grid, hydropower, a technology capable of fast ramps, is essential and all other generation types must be considered slow in comparison. In GB and CE, non-hydropower dominates the generation mix and technologies with slower ramps than hydropower plants but ramps faster than other generation types play important roles. Notably, hard coal is one of the slow generation types in GB but one of the fast types in CE due to the importance of nuclear and lignite generation in CE, which are even slower than coal. We categorized the generation types using SHAP values for the generation ramps to predict the RoCoF and relative ramping speeds (Figures 3D and 3E). We found that fast generation ramps drove the RoCoF. A positive ramp was associated with more positive frequency jumps. In contrast, ramps of slow generation types offset the RoCoF, leading to a negative correlation. The only exception here was the behavior of gas power plants in GB, which showed a negative correlation despite being fast. This is due to their role as the prime source of balancing reserve in GB.^47^ To summarize, the ramps no longer drove the RoCoF, but the RoCoF drove the ramps.

Notably, a model-agnostic data analysis does not produce such consistent results, as our features are strongly correlated (experimental procedures). For example, the Pearson correlation coefficient between nuclear ramps and RoCoFs in CE is positive (supplemental experimental procedures S3). Instead, the SHAP framework indicates a negative relationship, which we consistently explain with relative ramping speeds.

### Relating large control efforts to nonlinear dependencies

**Figure 4:**
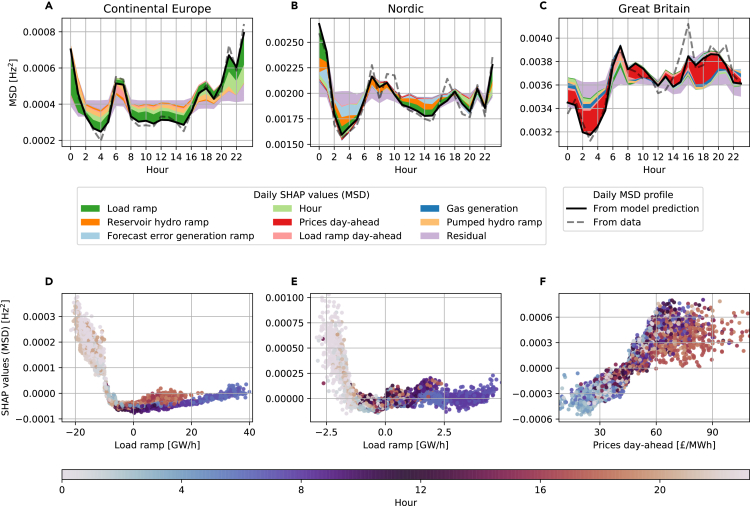
Explaining the daily average control effort with SHAP values (A–C) The daily average profile of the MSD (dashed line), i.e., the daily average control effort, is very well reproduced by the ML model (solid line), but its shape differs between the areas. We examine these differences with daily SHAP values for the MSD in Continental Europe (A), the Nordic grid (B), and Great Britain (C). Daily SHAP values (see experimental procedures) are sorted such that negative effects are plotted above the prediction line and positive effects below it. The importance of the plotted feature effect decreases the farther away the feature is from the prediction line. Less important features are aggregated in a residual variable. (D–F) For the Continental Europe (D) and Nordic (E) grids, the dependency plots of the most important daily SHAP effects reveal nonlinear relationships. These relationships explain the large control effort around midnight (color code), while the linear effect of prices explains the low control effort in GB during the night (F).

Frequency stability indicators often exhibit a complex nonlinear dependency on the features. Using the MSD, an indicator for the (primary) control effort,^33^ as an example, we found that the daily profiles of the MSD differed strongly between the three grids (Figure 4). These differences were well reproduced by the ML model and were explained using daily aggregated SHAP values (experimental procedures).

In CE, the control effort peaked around midnight (Figure 4A) due to the nonlinear effects of negative load ramps. Details on this relation are shown in a dependency plot (Figure 4D). Load ramps between −7 and +25 GW/h had a small negative effect on the MSD because such small ramps are easy to control. Outside this range, the effect increased strongly in a nonlinear and asymmetric manner. Negative load ramps had much larger effects than positive load ramps, and they occurred almost exclusively around midnight (see color code). In the Nordic daily profile, load ramps were also the most important feature (Figure 4B), and they showed a very similar nonlinear dependency (Figure 4E). In contrast, the daily MSD profile in GB strongly depended on day-ahead prices (Figure 4C), which had an almost linear dependency (Figure 4F). The control effort peaked during the day in response to high prices in the day-ahead market, while the MSD and the prices were low at night (00:00 to 04:00 h).

Notably, fluctuating renewables did not contribute strongly to the daily MSD profile in our model, although they are an important driver for frequency fluctuations in GB in general (cf. Figure 2). The observed differences between the synchronous areas could be due to different control regulations. For example, in GB, wind power farms must provide frequency control,^48^ and secondary control is allocated manually.^49^

### Explaining systematic imbalances with interactions

**Figure 5:**
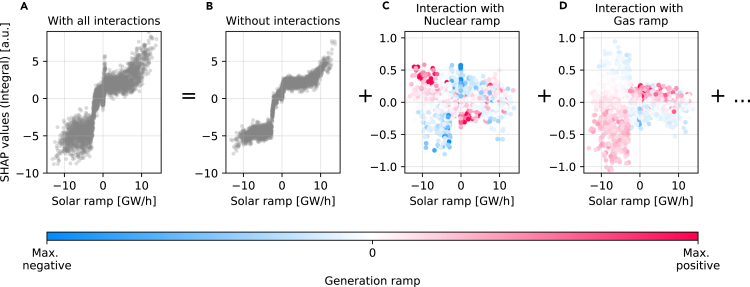
The effect of feature interactions on systematic power imbalances (A) CE is used to show the SHAP effects of solar ramps on the frequency integral, which are the most important effects in CE (Figure 2). The integral, which represents systematic imbalances, decreases for negative solar ramps, but the SHAP effects vary strongly, as indicated by their vertical dispersion. (B–D) Using SHAP interaction values (see experimental procedures), this dispersion was broken down into different interaction effects. These effects depend on the generation type, as negative nuclear ramps weaken the effect of negative solar ramps (C), while negative gas ramps lead to an amplified effect (D). Subtracting all interaction effects from the original SHAP values (A) yields the remaining effect of solar ramps (B), which is strongly altered.

The SHAP framework explains the role of different features and reveals how predictions depend on the *interaction* of features (see Figure 5 for an application of the integral in the CE grid and experimental procedures for technical details). It should be noted that the ML predictors for the other targets also displayed clear interactions. The most important features were solar and nuclear power ramps, which had a reverse dependency (Figure S13). Without interactions, the SHAP value increased gradually and nonlinearly with the solar ramp (Figure 5B). Strong negative ramps of solar power generation induced an ongoing shortage of power and thus led to negative integrals.

Interactions with nuclear and gas ramps altered the effect of solar ramps by up to 60%, leading to a strong vertical dispersion of the observed SHAP values (Figure 5A). In particular, negative nuclear ramps amplified the effect of negative solar ramps, while negative gas ramps dampened the effect on the integral (Figures 5C and 5D). These opposite interactions were again related to different ramping speeds. Nuclear power has the lowest ramping speed in CE, which meant that negative nuclear ramps amplified the continuous ramping behavior in interaction with solar ramps. In contrast, gas power had a fast ramp and therefore often provided balancing power, leading to the opposite effect. In general, these results demonstrate that interactions can influence how strongly a single feature affects power system stability.

## Discussion

Our model is based on explainable ML, and it predicts important indicators for power system frequency stability using external features, such as day-ahead electricity prices or total system load. Using real data (ex post analysis), our ML model outperformed the daily profile, a system-specific null model, by a factor of up to 16.3. Using only day-ahead available data, our ML models performed similarly in most cases. When SHAP values were calculated and examined, our model revealed important features and dependencies, and could thus pave the way for multiple applications.

Our model offers a versatile and substantially improved approach for analyzing risks and drivers of grid frequency stability. Previous data-driven studies analyzed the impact of one external feature on grid frequency fluctuations based on linear correlations^9^, ^10^, ^11^ or conditional probabilities.^7^ Such univariate analyses cannot be adjusted for effects of other features, which could be correlated with the feature of interest and may lead to incorrect conclusions (cf. Weißbach et al.^42^). Moreover, univariate, linear dependency analyses underestimate the effects of nonlinearities and ignore feature interactions. Our model includes multiple variables and fits nonlinear dependencies and interactions, which are made transparent by SHAP values. It breaks down the effect of correlated features (as discussed in Figure 3) and reveals otherwise undetectable nonlinear effects (Figure 4) and feature interactions (Figure 5). In addition, our model visualizes feature effects in the daily average evolution of frequency stability (Figure 4), which adds to the many useful visualization tools available in the SHAP framework. Our publicly available model^50^ and dataset^30^ can be used to predict any frequency stability indicator, thus offering a ready-to-use and flexible tool for analyzing power grid stability.

We applied our model to three different synchronous areas and identified options for improving power grid operation strategies. We discussed four examples for potential applications: (1) first, we showed how generation ramps drove the RoCoF and the nadir and revealed subtle differences between generation types and grids. These insights can help to optimize ramping behavior and mitigate DFDs^51^ and improve frequency quality. In particular, hydropower generation ramps should be optimized in response to other ramps. (2) Our results show that forecasting errors play an essential role in the Nordic grid. While TSOs are generally aware of the problem,^44^ the SHAP analysis provides a much more detailed view and reveals when and how these errors affect frequency stability. An example is given in Figure 4B, which shows that the features “forecasting errors of the generation ramps” are particularly important during the night. Our model identifies situations where forecasting errors are particularly critical and will thus improve risk awareness in grid operation. (3) Low inertia has been identified as a major threat for the stability of future power grids.^3^ Our analysis provides a more finely nuanced view on this topic. In our XAI model, inertia does not generally have a high feature importance. Instead, the impact of inertia on frequency stability is nonlinear and more pronounced for low inertia values (Figure S14). (4) Finally, the predictive power of our XAI model can be harnessed for online grid monitoring and preventive control measures. For instance, a model-predictive frequency restoration reserve has been proposed to mitigate DFDs.^52^ The applicability of such predictive control strategies can be extended by data-enabled methods (cf., e.g., Huang et al.^53^).

The main restrictions to our model performance and explainability are due to the quality of available power system data. Firstly, frequency deviations due to renewable fluctuations^7^ or load fluctuations^8^ occur on timescales that are smaller than the intervals of electricity trading. The limited time resolution of publicly available power system time series restricts both the performance of an ML model and its ability to suggest causal relationships because the time order of events is partly hidden. Secondly, all locations in a synchronous power grid affect the frequency deviations; but in large grid areas, such as CE, many countries provide no or only a limited amount of data.^14^ This further emphasizes the need for open data in energy system analysis and design.^54^

Our paper contributes to the applicability of XAI methods in energy systems and engineering sciences in general. Firstly, deriving causal relationships from data is a key challenge in modern ML techniques. With the power grid frequency, we provide a very well-suited test bed; while not all features and interactions are available, there is plenty of domain knowledge to interpret and cross-check XAI results. Secondly, we provide an excellent dataset^30^ for applications and the benchmarking of ML methods, such as causal inference or predictive models. While generation data are already publicly available,^36^ aggregating these for a whole synchronous area and combining them with frequency stability indicators yields a novel dataset for future usage. Finally, in Figure 4, we explored how daily aggregated SHAP data may be used to explain specific temporal profiles, which could be useful in other ML applications when dealing with strong daily or seasonal trends, e.g., in weather or traffic predictions.^55^^,^^56^

In conclusion, we hope that our work will trigger further applications of XAI in energy science, harnessing the strengths of modern ML tools while avoiding the drawbacks of black box approaches, which impede scientific insights^18^ and pose security risks.^19^ Our model provides insights by explaining feature effects with SHAP values in the context of the domain science. SHAP dependency and interaction plots visualize the knowledge learned by the model and offer individual explanations for each prediction. The most predictive associations then suggest causal relationships, which can then be validated by domain knowledge or further experiments. For example, we identify RoCoF-driving, RoCoF-offsetting, and RoCoF-balancing generation technologies by connecting our model results to physical ramping rates, thus suggesting different causal relationships. “Suggesting relations” is key here since neither boosted trees nor SHAP guarantee causal relationships but rather indicate associations based on the data. All in all, SHAP values alone do not provide scientific insights, but, when combined with domain knowledge, they can lead to further knowledge.

Future work includes explicitly forecasting the given indicators and classifying whether upcoming events could be problematic for grid operation. Once forecasts or other early warning and control methods have been implemented, our model will need to be retrained using these new, controlled datasets to derive the updated feature-target interactions. Furthermore, while we already outperform the daily profile, the performance of our tree-based predictor could be improved if further features were integrated and our model was compared with other ML prediction models. Finally, such regression models should be complemented by causal inference models to provide clear counterfactual statements and comparisons with XAI approaches.

## Experimental procedures

### Resource availability

#### Lead contact

Further information questions should be directed to the lead author, Johannes Kruse (jo.kruse@fz-juelich.de).

#### Materials availability

This study did not generate new unique materials.

### Data preparation of frequency stability indicators

In a modern AC power grid, the grid frequency is typically spatially synchronized and its dynamics can be represented by a single bulk time series on timescales of several seconds and more.^1^ In Europe, different synchronous areas exist, which are only inter-connected through DC links and hence display their own frequency dynamics and follow their own specific regulations. We modeled the bulk frequency dynamics for different synchronous areas in Europe, specifically for the CE, Nordic, and GB areas. We used pre-processed frequency time series $\tilde{f}\left(t\right)$ with a time resolution of τ=1 s,^13^ which were originally measured by regional TSOs.^57^, ^58^, ^59^

From the centered frequency time series $f\left(t\right)=\tilde{f}\left(t\right)-50$ Hz, we extracted four hourly stability indicators, which are directly relevant for power system operation.^32^^,^^33^ For the *i*th hour starting at time $t_{i}$, we calculated the (positive or negative) nadir, the integral and the MSD based on the hourly time steps $I_{i}=\left\{t_{i},t_{i}+\tau ,\ldots ,t_{i}+\tau \gamma \right\}$ with γ=3600:

$$\text{Nadir}\left(t_{i}\right)=f\left(\underset{t\in I_{i}}{\arg\;\max}\left|f\left(t\right)\right|\right)\text{,}$$

$$\text{Integral}\left(t_{i}\right)=\tau \sum_{t\in I_{i}}f\left(t\right),$$

$$\text{MSD}\left(t_{i}\right)=\frac{1}{\gamma }\sum_{t\in I_{i}}f^{2}\left(t\right)\text{.}$$

From the derivative of the frequency time series $\frac{\text{d}f}{\text{d}t}\left(t\right)$, we obtained the hourly (positive or negative) RoCoF by looking for the steepest slope within a window $W_{i}=\left[t_{i}-T,t_{i}+T\right]$ of length 2T around the beginning of the hour $t_{i}$:

$$\text{RoCoF}\left(t_{i}\right)=\frac{\text{d}f}{\text{d}t}\left(\underset{t\in W_{i}}{\text{arg max}}\left|\frac{\text{d}f}{\text{d}t}\right|\right)\text{.}$$

We estimated the derivative $\frac{\text{d}f}{\text{d}t}\left(t\right)$ using a low-pass filter on the frequency increments,^60^ i.e., by smoothing the increments Δf(t)=f(t)−f(t−τ) with a rectangular rolling window of length *L*. We chose the parameters *L* and *T* in such a way that they accounted for the different timescales of the RoCoF in the synchronous areas (supplemental experimental procedures S2). This resulted in a choice of L=T=60s for the CE and GB areas, while the Nordic area with its fast hydropower exhibited larger RoCoFs so that we chose L=T=30s instead.

### Data preparation of external features

We collected different power system time series as external features to predict frequency deviations in Europe. We retrieved six different sets of publicly available time series from the ENTSO-E transparency platform.^36^ These sets comprise the day-ahead load forecast, day-ahead scheduled generation, day-ahead wind and solar power forecast, day-ahead electricity prices, actual load, and actual generation per production type. Most of the time series are available on an hourly basis. Since we predicted stability indicators on an hourly basis, we downsampled a few higher-resolution time series to a common hourly resolution.

We then aggregated the data within the three synchronous areas. Since time series from ENTSO-E are only available for smaller regions within the synchronous areas (i.e., countries), we added up the load and generation data within each area. To aggregate the price data, we calculated area-wide averages weighted by the regional mean load. The time series from the ENTSO-E transparency platform contained multiple missing or corrupted data points,^14^ which required a careful aggregation and cleansing procedure (supplemental experimental procedures S1). We deemed area-wide feature aggregation necessary because all locations within the synchronous power grid contribute to large frequency deviations.^1^ We additionally prepared selected country-level data for the CE and the Nordic areas. The area-wide aggregated features resulted in a similar or higher model performance than country-level data (supplemental experimental procedures S5). Therefore, we decided to use area-wide aggregated features for this publication. An overview of the available (aggregated) features per area is available in supplemental experimental procedures S1.

Finally, we engineered additional meaningful features based on the hourly ENTSO-E time series $X\left(t_{i}\right)$, which comprise both day-ahead forecast data $X_{D-1}\left(t_{i}\right)$ and actual data $X_{D}\left(t_{i}\right)$. For each hourly interval Δt=τγ, we introduced ramp features (slopes) $\left(X\left(t_{i}\right)-X\left(t_{i}-\Delta t\right)\right)/\Delta t$, which are inspired by the importance of generation ramps for the CE frequency dynamics.^4^ We also added forecast errors $X_{D-1}\left(t_{i}\right)-X_{D}\left(t_{i}\right)$ and the artificial features of hours (of the day), weekdays, and months. To include the total available inertia as a feature, we calculated the sum of the synchronous generation which approximates to the time-dependent inertia.^38^

In summary, our dataset comprises hourly time series of 4 stability indicators (model outputs or targets) and 66 external features (model inputs) for the years 2015–2019. The dataset is available on Zenodo^30^ and our scripts for downloading and preparing the dataset are online at GitHub.^50^

### GTB model

To predict indicators of frequency stability from external features, we used GTB. Tree-based ensemble methods, such as GTB, are complex, nonlinear ML models, which makes them suitable for predicting the nonlinear behavior of power grids.^1^ They offer a quick method of calculating SHAP values, thus facilitating efficient post-modeling explanation.^24^ In addition, they are immune to the effects of feature outliers and perform inherent feature selection, making them robust to the inclusion of correlated or irrelevant features.^16^ This is beneficial for our dataset, which exhibits strongly correlated features (supplemental experimental procedures S3) as well as outliers due to bad data quality (supplemental experimental procedures S1).

To fit our GTB model, we used XGBoost, which is a scalable gradient tree boosting system that provides state-of-the-art results for many ML applications.^43^ We randomly split our data into a training set (64%), a validation set (16%), and a test set (20%). To optimize the hyperparameters of the XGBoost model, we performed a grid search over selected parameter values and evaluated the performance via 5-fold cross-validation on our training set. To determine the number of trees in the XGBoost models, we performed early stopping on the validation set. Finally, we concatenated the training and validation sets, retrained the model on this data with optimal hyperparameters, and tested its performance on the unseen test set. We also calculated the SHAP values on the test set. The detailed implementation in Python code is available on GitHub^50^ and the sets of final hyperparameters are online at Zenodo.^30^

To quantify the model performance, we evaluated the R^2^ score, which quantifies the proportion of variability explained by the model. Predicting the true targets results in a score of 1, while always predicting the mean of the target gives a score of 0. To benchmark our predictor, we compared its performance with the daily profile prediction. The daily profile, i.e., the daily average evolution of a target, is the most important recurring pattern of frequency dynamics.^61^ Predicting the stability indicators based on their daily profiles thus represents an important null model. Our GTB model consistently outperformed the daily profile for all areas and indicators (see supplemental experimental procedures S5 for a detailed performance evaluation).

### Model interpretation with SHAP

SHAP values can explain the output of any ML model.^23^ Based on the game-theoretical Shapley values, they attribute a model output to the individual effects of each input feature. In particular, SHAP values quantify the marginal effect of including a feature into the prediction and comparing them with a randomized baseline.^24^^,^^62^ Within the class of additive feature attributions, they guarantee certain optimal properties, such as local accuracy and consistency.^22^ As they are locally accurate, the SHAP values always add up to the total model output. Consistency guarantees that a SHAP value does not decrease if the corresponding feature contributes more to the prediction when the model is altered.

SHAP values represent the feature effects on individual model outputs relative to the base value, which is given by the average prediction (cf. Figure 1). By combining many of these local explanations, SHAP values also offer global insights.^24^

The mean absolute SHAP value measures the global importance of a feature within a model. We identified the five most important features for each stability indicator and area (Figure 2). Figure 2 also displays feature importances for the *union* of these feature sets, i.e. features with an importance rank below five are also displayed. In addition to global feature importances, dependency plots show how the effect of a feature changes with the value of the feature (e.g., Figure 4D). Notably, these dependencies differ from observing relationships in scatterplots or between targets and features in a simple correlation analysis. Such model-agnostic methods cannot distinguish the effect of two correlated features. In contrast, we estimated interventional SHAP values, which quantify the marginal feature effect in the model by breaking down correlations with other features.^63^^,^^64^

In addition to first-order attributions, SHAP offers interaction values that attribute the model output to pairs of interacting features.^24^ Interaction values decompose the first-order SHAP effects into diagonal effects and pairwise interaction effects (such as in Figure 5). The interaction effects therefore explain the vertical dispersion within the first-order SHAP dependency plots, thus offering scientific insights as well as additional consistency checks for the model applications.

Finally, there is a fundamental difference between predictive models and causal models.^65^ Predictive models try to infer the conditional probability of the target given the feature variables by fitting associations. Causal models identify the effect on the target when manipulating or intervening on a feature. ML models, such as the boosted trees used here, are typically predictive models. Using XAI methods to explain how these ML models work reveals only associations learned from the data.^21^ In particular, using SHAP values to explain predictive models does not necessarily reflect causal effects.^66^ However, causation involves correlation so that predictive and explainable ML models can suggest causal dependencies, which then have to be further validated, e.g. by domain knowledge or causal inference methods.

### Aggregated SHAP values

To explain daily average profiles of the model predictions, we visualized the SHAP values in a way that builds on their additivity. Due to their property of “local accuracy,” the prediction f(t) at every point in time *t* can be written as a sum of the respective SHAP values,

(Equation 1) $$f\left(t\right)=\varphi_{0}+\sum_{j=1}^{N}\varphi_{j}\left(t\right),$$

where $\varphi_{j}\left(t\right)$ is the SHAP value of feature *j* at time *t*. This property of SHAP values enables a new application in the analysis of daily profiles and other recurrent patterns. The daily profile of the prediction is the average $\langle f\left(t\right)\rangle_{h}$ for the hour *h* over all days. Based on the SHAP values $\varphi_{j}\left(t\right)$ for feature *j* (j=1,…,N) and their base value $\varphi_{0}$,^24^ we decomposed the daily profile as follows:

$${\left\langle f\left(t\right)\right\rangle }_{h}={\left\langle \varphi_{0}+\sum_{j=1}^{N}\varphi_{j}\left(t_{i}\right)\right\rangle }_{h}=\varphi_{0}+\sum_{j=1}^{N}{\left\langle \varphi_{j}\left(t\right)\right\rangle }_{h}.$$

The daily aggregated SHAP values ${\left\langle \varphi_{j}\right\rangle }_{h}$ then explain the daily profile of the prediction. To display the daily SHAP values, such as in Figures 4A–4C, we identified the three most important features according to their average effect $\frac{1}{24}\sum_{h=1}^{24}\left|{\left\langle \varphi_{j}\right\rangle }_{h}\right|$ on the daily profile in each area. In Figures 4A–4C, we then visualized these features from the union of these sets to display the most important daily SHAP values. The remaining daily SHAP values were aggregated and displayed as a residual variable.

Finally, we add three notes on the interpretation of (daily) aggregated SHAP values. (1) We note that the aggregated SHAP values do *not* coincide with SHAP values of a model trained on the aggregated data. This must be taken into account when interpreting the results. (2) Due to the nonlinearity of an ML model, a large daily SHAP value does not necessarily correspond to a large average for the corresponding feature in that hour. (3) Second-order interactions between features are “fairly” distributed between first-order SHAP values according to the classical Shapley values,^24^ i.e., large daily SHAP values can partly relate to strong interaction within this specific hour. To further resolve interactions within the daily SHAP values, the additivity of second-order SHAP values can be used to generate daily profiles of the interactions. This is beyond the scope of this paper.

### Relative ramping rates

We used relative ramping rates to validate our SHAP results, particularly for the prediction of the RoCoF. In particular, we quantified the relative ramping speed of each conventional generation technology *k* within a synchronous area. The ramping speed ${\tilde{s}}_{k}$ is determined both by the absolute change of generation $\Delta X_{k}$ and the timescale $\lambda_{k}$ on which the generator adapts its output to the new set point:

$${\tilde{s}}_{k}:=\frac{\Delta X_{k}}{\lambda_{k}}.$$

We approximated the typical value of $\Delta X_{k}$ with the median of the absolute generation changes $\Delta X_{k}\approx \underset{t_{i}}{\text{Median}}\left|X_{k}\left(t_{i}-\Delta t\right)-X_{k}\left(t_{i}\right)\right|$. The *relative* ramp speed $s_{k}$, compared with the fastest technology *m* within the area, then reads

$$s_{k}=\frac{{\tilde{s}}_{k}}{{\tilde{s}}_{m}}=\frac{\Delta X_{k}}{\Delta X_{m}}\frac{\lambda_{m}}{\lambda_{k}}\approx \frac{\Delta X_{k}}{\Delta X_{m}}\frac{r_{k}}{r_{m}}.$$

Finally, we approximated the ratio of timescales $\lambda_{k}$ by using the inverse ratio of technology-specific ramping rates $r_{k}$.^67^ The technology *m* with the largest absolute ramping speed was determined by the maximum value of $\Delta X_{k}r_{k}$.

## Data Availability

The dataset to reproduce our results is available on Zenodo: https://doi.org/10.5281/zenodo.5118352. The Python code used to create our results and the figures is also archived on Zenodo: https://doi.org/10.5281/zenodo.5497609.

## Supplemental information

Document S1. Supplemental experimental procedures S1–S6, Figures S1–S14, and Tables S1 and S2

Document S2. Article plus supplemental information
