# Supplementary material for: Revealing drivers and risks for power grid frequency stability with explainable AI
Source: Patterns (N Y). 2021 Oct 8;2(11):100365. doi: 10.1016/j.patter.2021.100365 (PMC8600233; doi:10.1016/j.patter.2021.100365)
Supplement: Document S1. Supplemental experimental procedures S1–S6, Figures S1–S14, and Tables S1 and S2 [file mmc1.pdf]

Patterns, Volume *2*

Supplemental information

Revealing drivers and risks for power grid frequency stability with explainable AI

Johannes Kruse, Benjamin Schäfer, and Dirk Witthaut

SUPPLEMENTAL EXPERIMENTAL PROCEDURES S1: EXTERNAL FEATURE AGGREGATION AND DATA CLEANSING

To model frequency stability indicators, we collect publicly available times series of external features from the ENTSO-E Transparency platform.[1] For the synchronous areas investigated here, we aggregate the ENTSO-E time series, which are originally only available for smaller regions within the areas (e.g., countries). However, the time series contain many missing data points, so that we need a careful procedure to aggregate the region contributions within the synchronous areas.

Firstly, we specify region types for which we obtain the best data quality. In all but a few cases, we retrieve country level data. Only in Continental Europe, we retrieve bidding zone data for Italy (North, Center North, Center South, South and Sicilia) and control zone data for Germany (TenneT, TransnetBW, 50Hertz, Amprion), as data quality is better for these regions. For Denmark, we also retrieve bidding zone data, as one zone belongs to the Continental Europe area, while the other belongs to the Nordic area.

Secondly, in the Continental Europe and the Nordic areas, we aggregate the region contributions and propagate missing data points through the data set. For each feature and region, we mark missing time steps as "NaN". Then, during aggregation, we propagate missing values by setting the sum of region contributions to NaN if at least one of them is NaN. For example, if the German load data had a missing value at 10:00 on the 6th of June 2018, the Continental European aggregated load data would have a NaN at this time step. Finally, we clean all aggregated features together with the grid frequency data. In particular, we omit a time step from the *whole* data set if at least one feature is NaN or the corresponding frequency measurements contains missing or corrupted values. This cleansed data set is used in model training and testing.

Notably, we will not have many data points left in the aggregated data set, if the region contributions contain too many NaNs. To avoid this problem, we collect the time series of each feature and region, which we call the *region-variable contribution*, and sort them according to their NaN share. Then, we successively add up the region-variable contributions with increasing NaN share. A contribution is only added if the NaN share S of the aggregated data set *including* the new contribution does not exceed a certain threshold S_T. If the data exceeds the threshold ($S > S_T$), we omit the contribution from the aggregation. We choose $S_T = 37\%$, which we found to result in a good balance between having enough data and including as many contributions as possible. As an example, consider a (hypothetical) area consisting of 2 countries, "A" and "B", with two features "Load" and "Total generation". Initially, we add load data from country A as this (hypothetically) has the lowest NaN share of 30%. Then, we add the total generation from country B with a NaN share of 31%. The contribution is not omitted, since the time steps with NaN values overlap and the final NaN share of the whole data set only yields 33% thus staying below the threshold. However, the last two region-variable contributions might not be added, as they introduce to many new NaN values.

This procedure omits a certain amount of data but allows us to retain a large sample size. In Continental Europe, most of the omitted contributions would increase the feature value by less than 30% on average (Figure S1A) and all omitted feature contributions are smaller than 5% of the total mean load (Figure S1B). In the Nordic area, we only omit the Finish day-ahead solar power forecast, accounting for 0.03% of the area total mean load, and in GB there are no omissions. The aggregated data sets obtained from this procedure contain more than 26800 data points in each area (Table S2). We thus generate large data sets to efficiently learn structures in the data, while still representing most of the load and generation within the areas.

Finally, we obtain 25 different (aggregated) times series of external features. Combining them with additional engineered features, such as forecast errors, we end up with 66 different external features (Table S1), which both contain day-ahead available features (such as the load forecast) and ex-post available features (such as the actual generation per type). None of the synchronous areas exhibits all 66 features and the number of model inputs thus varies between 50 and 64 (Table S2).

SUPPLEMENTAL EXPERIMENTAL PROCEDURES S2: ROCOF EXTRACTION

The Rate of Change of Frequency (RoCoF) is an indicator of frequency stability, which we use in our study. We extract the RoCoF at the beginning of each hour by smoothing the frequency increments with a rolling window of length L and then looking for the maximum (absolute) RoCoF within a window of $\pm T$ around the full hour.

We choose the values of L and T according to the typical time scale of the RoCoF in the three different synchronous areas. The average hourly evolution of the absolute frequency deviation indicates this time scale (Figure S4). In Continental Europe and Great Britain, the average deviation reaches its maximum 60 s after the full hour, while the Nordic area exhibits its peak already after 30 s. We thus choose $L = T = 60$ s in the Continental Europe and Great Britain areas, but a shorter time scale of $L = T = 30$ s in the Nordic grid area.

Ex-post	Ramps [MW/h]	Load ramp, Total generation ramp, Biomass ramp, Coal gas ramp, Fossil peat ramp, Gas ramp, Geothermal ramp, Hard coal ramp, Lignite ramp, Nuclear ramp, Offshore wind ramp, Onshore wind ramp, Oil ramp, Other ramp, Other renewables ramp, Pumped hydro ramp, Reservoir hydro ramp, Run-off-river hydro ramp, Solar ramp, Waste ramp
	Generation and load [MW]	Load, Total generation, Synchronous generation, Biomass generation, Coal gas generation, Fossil peat generation, Gas generation, Geothermal generation, Hard coal generation, Lignite generation, Nuclear generation, Oil generation, Other generation, Other renewable generation, Pumped hydro generation, Reservoir hydro generation, Run-off-river hydro generation, Solar generation, Waste generation, Wind offshore generation, Wind onshore generation,
	Forecast errors of generation and load [MW]	Forecast error load, Forecast error total generation, Forecast error solar, Forecast error offshore wind, Forecast error onshore wind
	Forecast errors of ramps [MW/h]	Forecast error load ramp, Forecast error generation ramp, Forecast error solar ramp, Forecast error offshore wind ramp, Forecast error onshore wind ramp
Day-ahead	Generation and load [MW]	Load day-ahead, Scheduled generation, Solar day-ahead, Offshore wind day-ahead, Onshore wind day-ahead
	Ramps [MW/h]	Load ramp day-ahead, Generation ramp day-ahead, Solar ramp day-ahead, Offshore wind ramp day-ahead, Onshore wind ramp day-ahead
	Other	Price ramp day-ahead [Currency/MWh/h], Prices day-ahead [Currency/MWh], Hour, Weekday, Month

Table S1. All external features in the data set. The units correspond to those used in our publicly available data set.[2]

Area	Number of features	Number of data points
Continental Europe	64	26857
Nordic	58	37154
Great Britain	50	43240

Table S2. Properties of our data sets.

SUPPLEMENTAL EXPERIMENTAL PROCEDURES S3: BASIC CORRELATION ANALYSIS

A basic correlation analysis between external features and frequency stability indicators can already reveal interesting dependencies. However, this model-agnostic correlation analysis does not account for correlations among the features, which might affect the correlation coefficient between a feature and the stability indicator. Following the main text, we demonstrate this for the effect of nuclear ramps on the RoCoF in Continental Europe.

As depicted in Figure S5, there are various strong correlations between features in all three grid areas. For example, nuclear power generation is positively correlated with the load in Continental Europe. In Figure S6, the features are correlated with our four stability indicators. We observe that nuclear ramps are positively correlated with the RoCoF in Continental Europe, which is not consistent with our SHAP results (see main text). We can explain the positive correlation of nuclear ramps with the hidden relationships to other variables, such as load ramps. Load ramps have a positive effect on the RoCoF in Continental Europe (Figure S10). Due to the strong correlation between load and nuclear power generation (Figure S5) the effect of load ramps can thus "leak" into the correlation coefficients of nuclear ramps. This can explain why we observe a positive correlation between nuclear ramps and the RoCoF in Continental Europe, although nuclear ramps are RoCoF-offsetting in this area, as revealed by SHAP analysis in the main text.

SUPPLEMENTAL EXPERIMENTAL PROCEDURES S4: DETERMINISTIC FREQUENCY DEVIATIONS

Deterministic frequency deviations (DFDs) occur at the beginning of electricity trading intervals.[4] The generation is adapted in a step-wise manner at the beginning of these intervals, which are mostly hourly time periods. The mismatch between the step-wise generation and the continuously evolving load generates an instantaneous power imbalance, which causes a deterministic frequency jump at the beginning of the hour.

Such DFDs are an important factor for frequency stability in Continental Europe. This is indicated by the time within the hour when the absolute frequency deviation peaks ("Nadir occurrence time"). Figure S12 shows the histograms of these Nadir occurrence times within the hour. In Continental Europe, most of the Nadirs occur in the

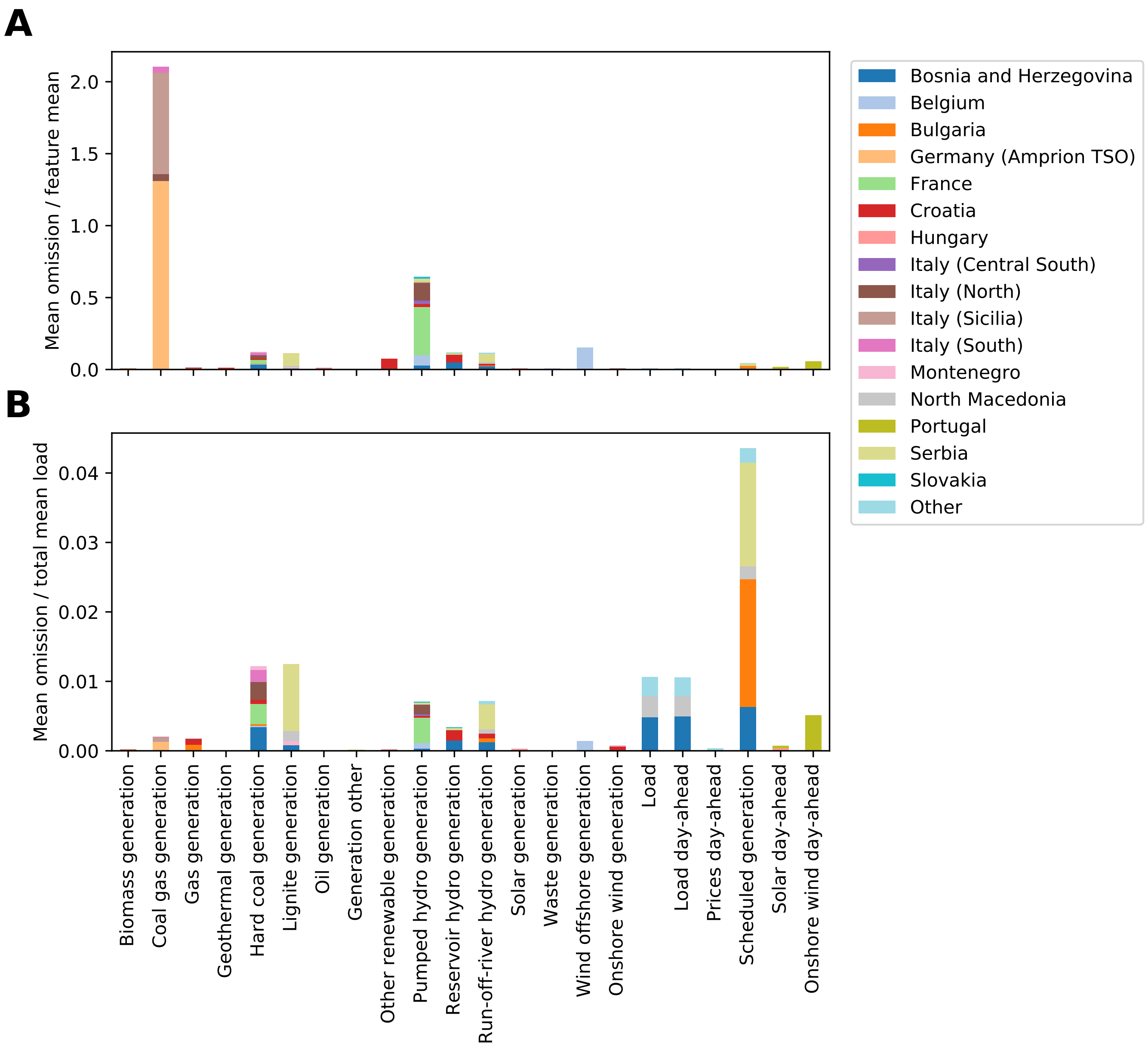

Figure S1. Omitted data contributions in Continental Europe. (a): We quantify the omitted values with the mean omitted feature value relative to the mean of the included features. The legend indicates the regions (mostly countries) where the omitted contributions come from. Regions with omitted (relative) contributions below 0.8% are aggregated in the "Other" variable. (b) The mean omitted feature value relative to the total mean load of the synchronous area remains below 5%.

first five minutes, which indicates their strong connection to the deterministic electricity trading. In contrast, large deviations in Great Britain occur much more often during the hour and not only at the beginning. This indicates that DFDs play a smaller role in Great Britain than in Continental Europe. The Nordic area is in between, showing strong hourly DFDs as well as Nadirs within the hour.

An extension of the work presented here could consider a threshold for the nadir and turn our regression machine learning task into a classification taks: Will the nadir of the next hour be above or below the security threshold?

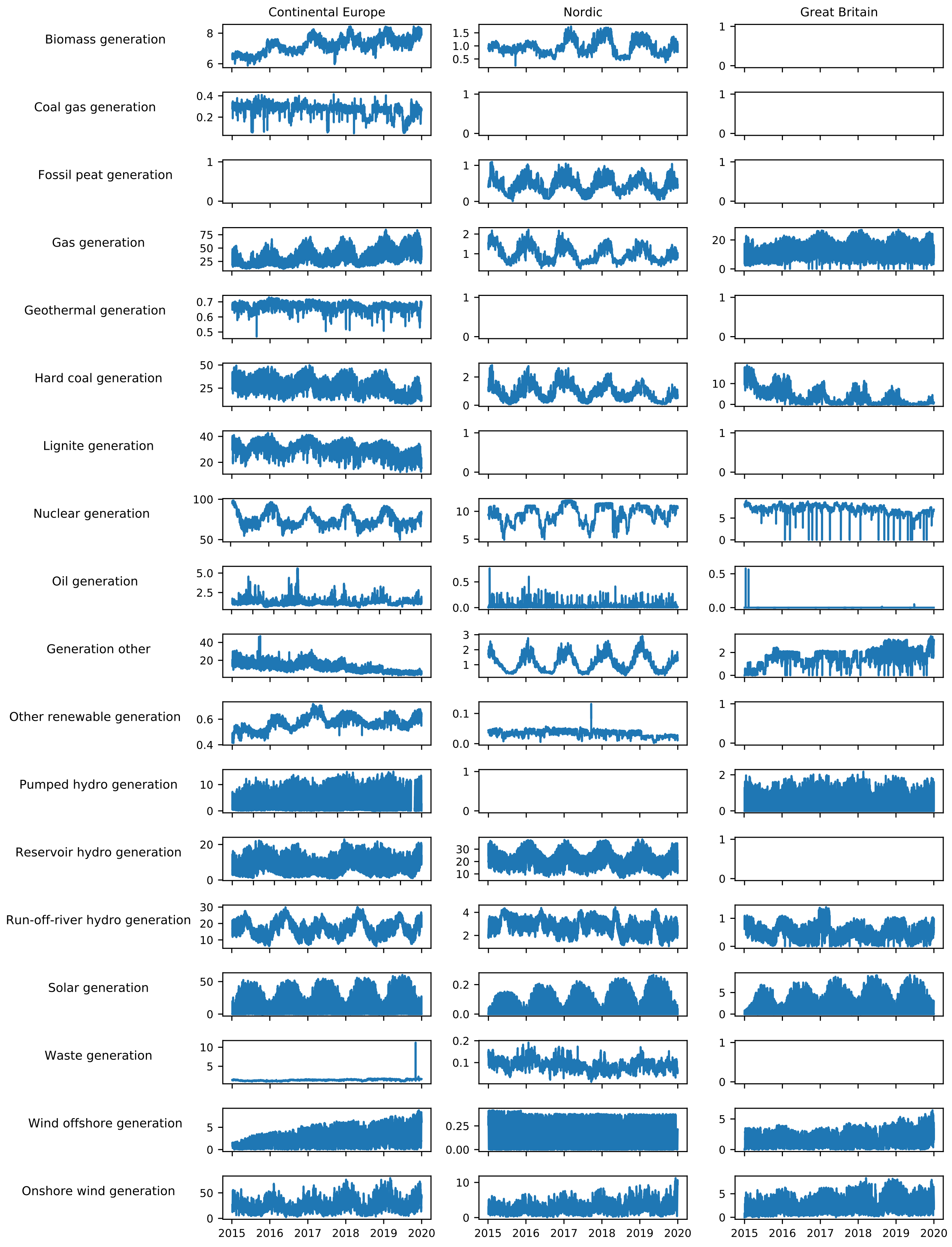

Figure S2. All (aggregated) time series from the ENTSO-E transparency platform:[1] Actual generation per type (in GW).

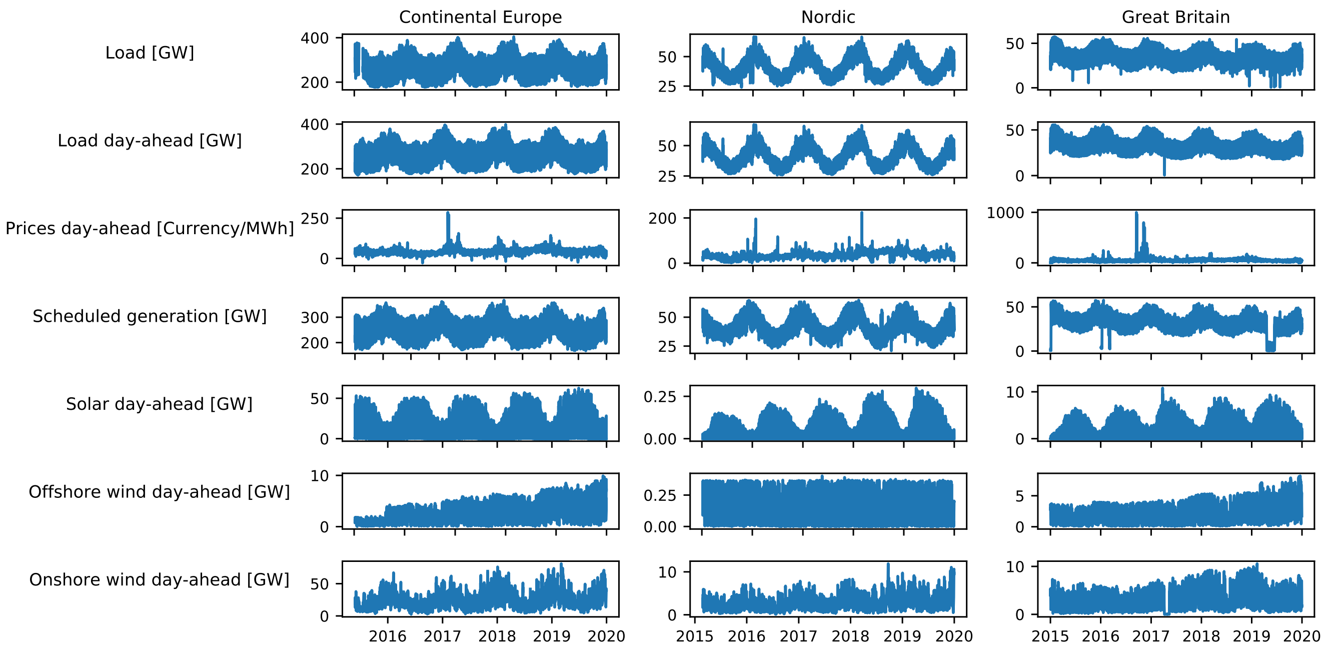

Figure S3. All (aggregated) time series from the ENTSO-E transparency platform:[1] All features except from actual generation per type.

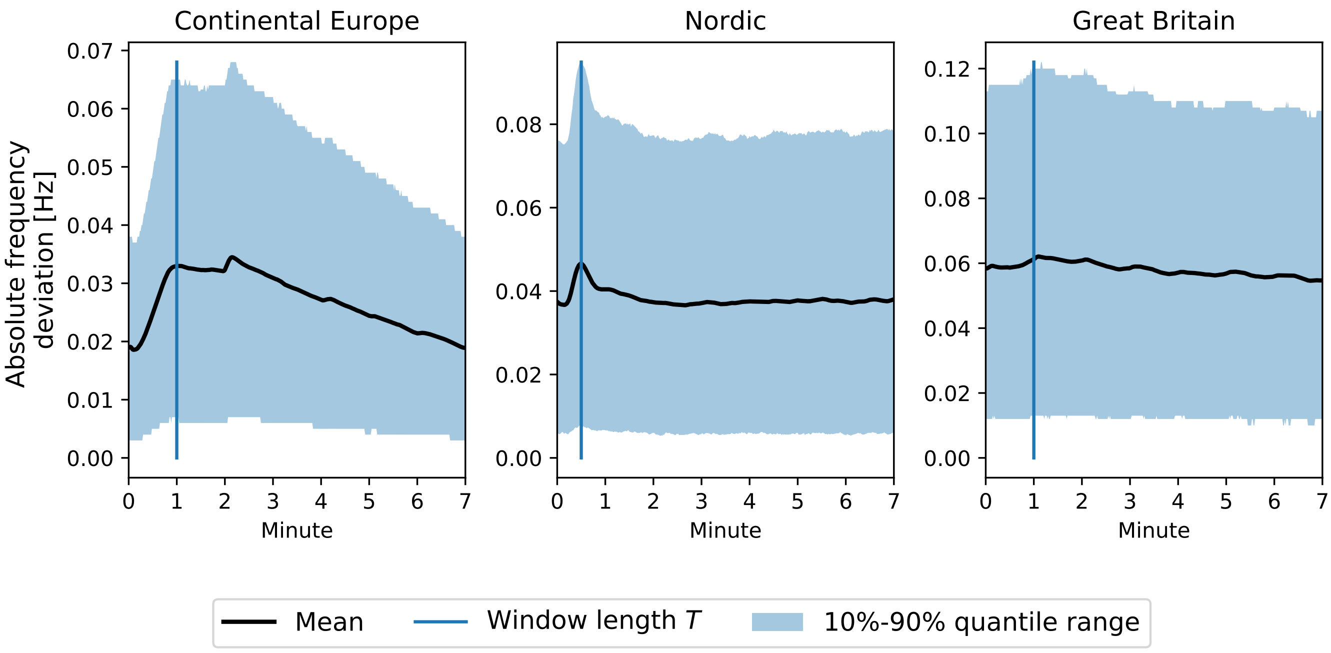

Figure S4. Evolution of hourly absolute frequency deviations. We display the mean evolution of the absolute frequency deviation during the first seven minutes of an hour. The deviation peaks at the beginning of the hour due to the impact of electricity trading.[4] The time scale of this initial increase depends on the grid area. We choose the window length T for the RoCoF extraction according to this time scale.

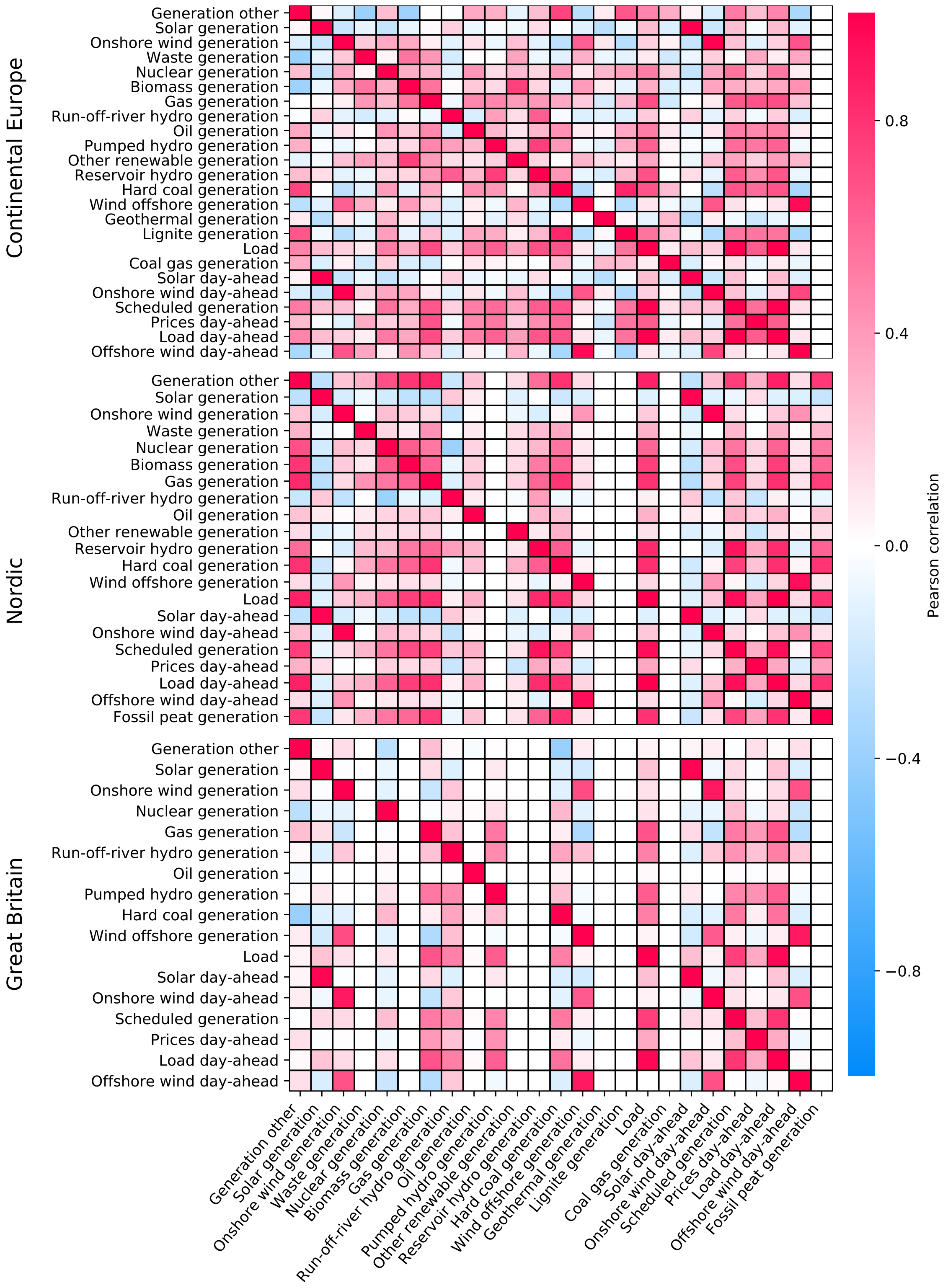

Figure S5. Pearson correlation coefficients between external features. To improve the visibility, we exclude our additional, engineered features from this plot. Features with 0 correlation everywhere have no data in a specific grid (e.g., Fossil peat data is only available in Nordic).

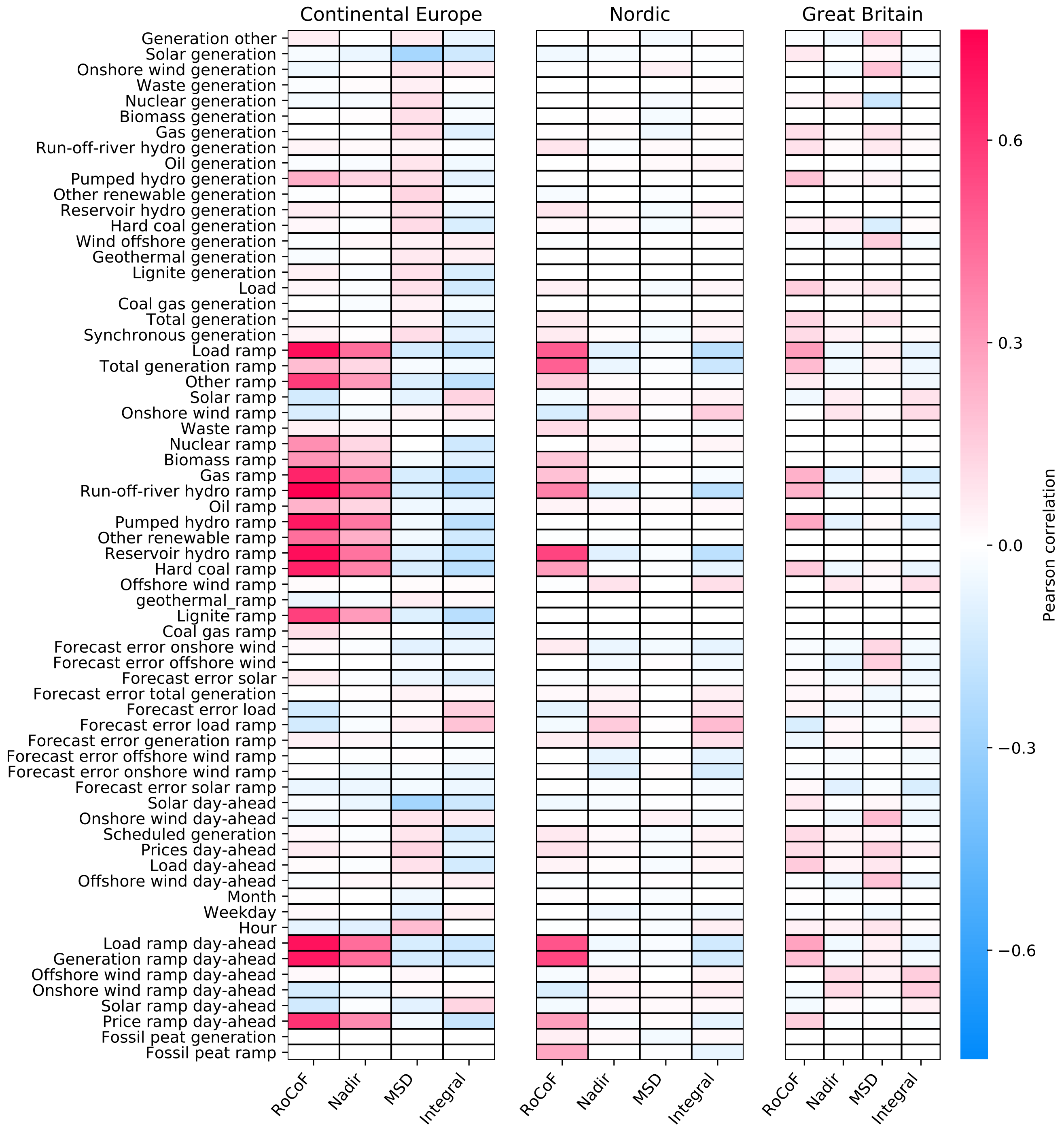

Figure S6. Pearson correlation coefficients between external features and frequency stability indicators. The scale of the colour code is adjusted to the maximum absolute correlation value C_{max} and thus ends at $\pm C_{max}$.

SUPPLEMENTAL EXPERIMENTAL PROCEDURES S5: PERFORMANCE EVALUATION OF THE MACHINE LEARNING MODEL

We evaluate the performance of our Gradient Tree Boosting (GTB) model in terms of the R^2-score, which quantifies the proportion of variability explained by our model. A perfect prediction would result in a score of 1, while predicting the mean of the target results in a score of 0. As a benchmark, we compare the GTB model to the daily profile, which is an important null model for frequency dynamics. In particular, we quantify the gain over the daily profile, which

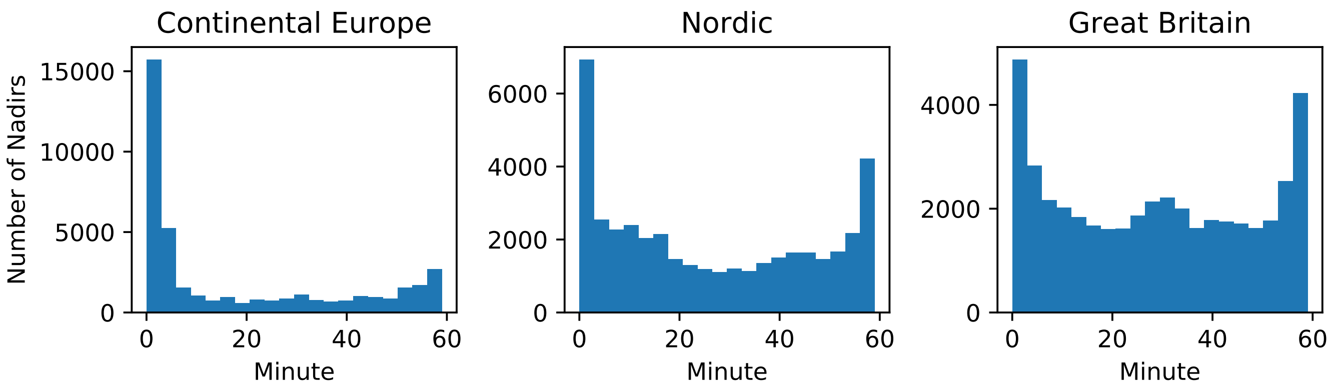

Figure S7. Distribution of Nadir occurrence time. The occurrence time of the nadir is the minute within the hour where the absolute frequency deviation reaches its peak. Its distribution within the hour varies between the grid areas, thus indicating the different importances of DFDs for the grid frequency dynamics.

is the model performance divided by the daily profile performance. Finally, we examine the importance of area-wide feature aggregation and the possibility to predict stability indicators day-ahead. Note that the GTB model used in our main text is referred to as the *full model*. It builds on area-wide aggregated features containing both day-ahead *and* ex-post available data.

The GTB model performs best in Continental Europe, while the performance gain over the daily profile is largest in Great Britain. Figure S8 displays the R^2-score for each stability indicator and each area. We obtain the best predictions in Continental Europe ($R^2 \sim 0.7$) and the lowest scores in Great Britain. In contrast, the performance gain over the daily profile is largest in Great Britain (maximum 16.2) and smallest in Continental Europe (maximum 3.4), while the Nordic area is in between (maximum 7.6). Frequency dynamics in Continental Europe are rather deterministic compared to the stochastic dynamics in Great Britain. Therefore, the prediction is easier and the additional gain through Machine Learning is smaller in Continental Europe. Consistently, the GTB performance is best for the RoCoF as this indicator most strongly reflects the hourly deterministic frequency jumps.

The model performance depends on whether we choose area-wide aggregated features or country-level data (Figure S8). In the Nordic area, we obtain a lower performance when using data from only the largest country (Sweden) instead of aggregating it area-wide. In Continental Europe, the largest country model (using Germany) performs similar to or worse than the aggregated model, and choosing a smaller country (Switzerland) reduces the performance even further. As the grid frequency is affected by all locations within the grid, it is not surprising that data aggregation is important. Overall, the area-wide feature aggregation yields better results than regional data among the areas.

Using only day-ahead available data in our GTB model already outperforms the daily profile for all stability indicators and areas (Figure S9). In Great Britain, the day-ahead model exhibits the strongest performance gain over the daily profile (maximum 8.9), followed by the Nordic area (maximum 3.0) and Continental Europe (maximum 2.6). However, adding ex-post data in the full model can strongly improve the performance, especially in the Nordic area. We quantify this effect in terms of the gain over the day-ahead model, i.e., the full model performance divided by the day-ahead model performance. In the Nordic area, the gain of the full model over the day-ahead model is the largest (maximum 2.6), while it is lowest in Continental Europe (maximum 1.4). The benefits of adding ex-post data in the Nordic area stems from the importance of forecasting errors for the prediction (see main text).

SUPPLEMENTAL EXPERIMENTAL PROCEDURES S6: ADDITIONAL RESULTS WITH SHAP VALUES

We use SHAP values to explain our Machine Learning model for frequency stability indicators. An overview of the most important SHAP dependencies in our model is available in Figures S10, S11, S12 and S13 for each of the four stability indicators. In many cases, we observe non-linear dependencies, which underlines the importance of using a non-linear, complex Machine Learning model such as Gradient Tree Boosting.

Here, we further discuss the effect of synchronous generation on frequency stability indicators. The (total) synchronous generation, which we use as a proxy for the total inertia within the power grid, is not among the eight most important features (Figures S10, S11, S12 and S13). Its feature importance is ranked on places between 19 and 64 in CE, between 38 and 52 in the Nordic area and between 25 and 40 in GB. Overall, the average effect of the (approxi-

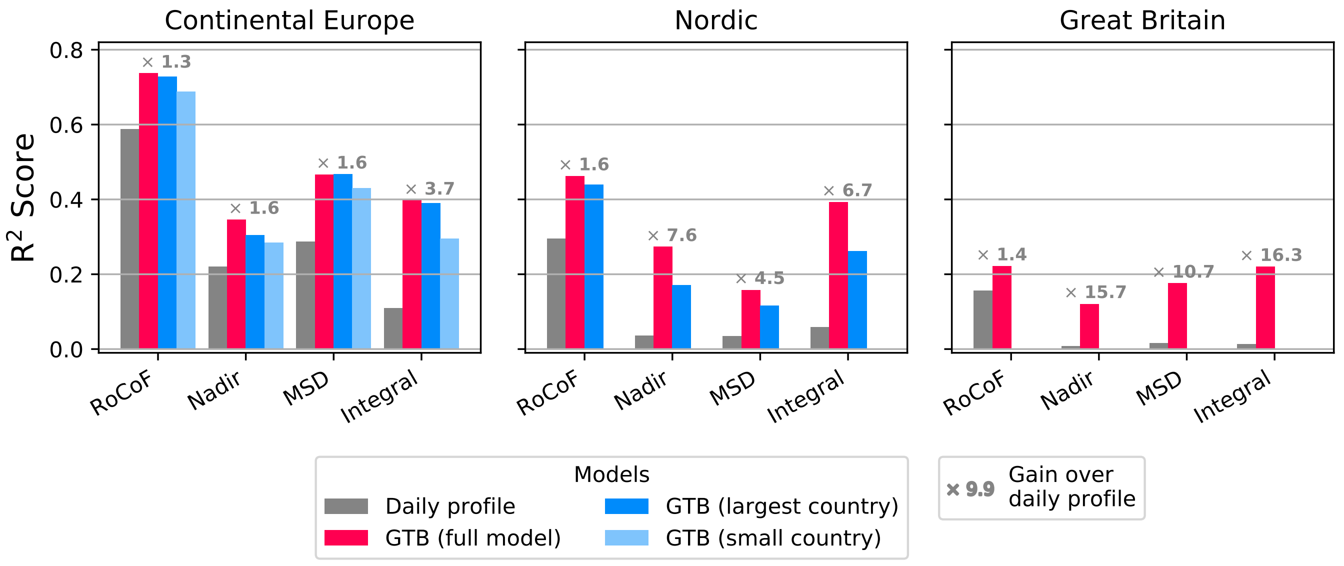

Figure S8. Performance of the full GTB model. The numbers on the bar plots indicate the gain of the GTB model over the daily profile. The full model comprises day-ahead and ex-post available features, which are aggregated area-wide. The country-level models use features from the largest country, i.e., the country with the largest power demand. In Continental Europe, we additionally introduce a model using data from a small country (with smaller average load), which is Switzerland. We retrieve the country-level data from the ENTSO-E transparency platform[1] and construct the same features as in the full model (Table S1).

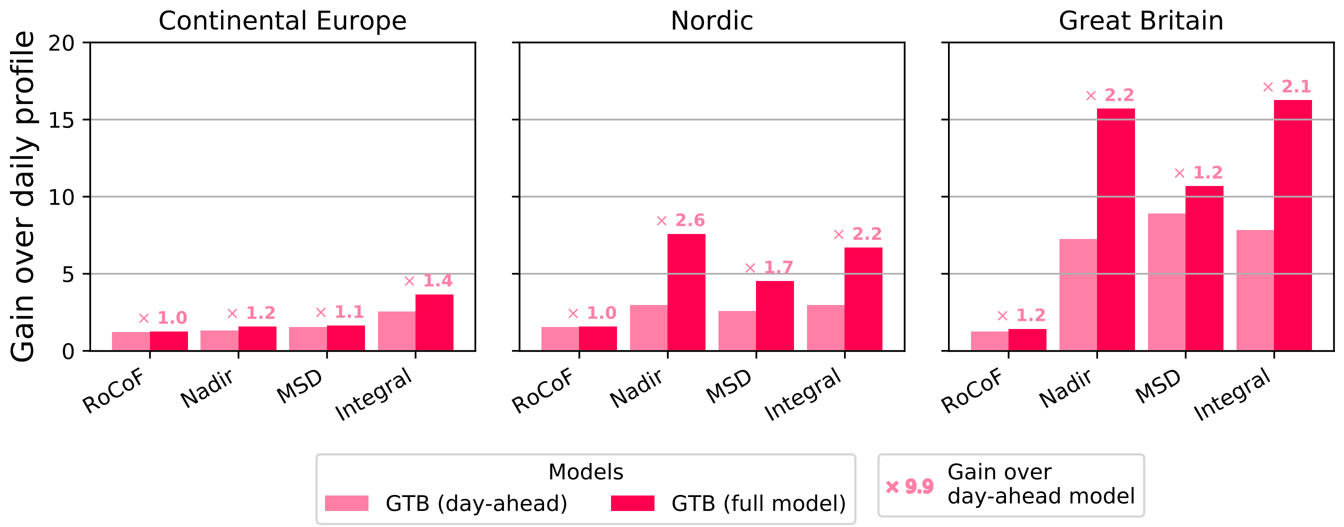

Figure S9. Performance of the day-ahead GTB model. We compare the full model and the day-ahead model in terms of their gain over the daily profile. The numbers on the bar plots indicate the gain of the full model over the day-ahead model.

mated) inertia on the aggregated stability indicators is thus relatively low compared to the most important features. Among the areas, the total synchronous generation is most important in Great Britain, as the feature is constantly among the 40 most important variables. This is consistent with the high share of renewable energy sources in the British power system and the resulting low-inertia situations.[3] The effect of the inertia in Great Britain is depicted in the dependency plots of Figure S14. For all stability indicators, we observe the maximum (absolute) effect of the synchronous generation at low feature values. This is particularly evident for the MSD and the Integral, where the effect of values larger than 20 GW is near to zero. In conclusion, the (approximated) inertia mostly affects frequency stability in Great Britain in extreme situations of low inertia, but the average effect of this feature on our aggregated stability indicators is negligible.

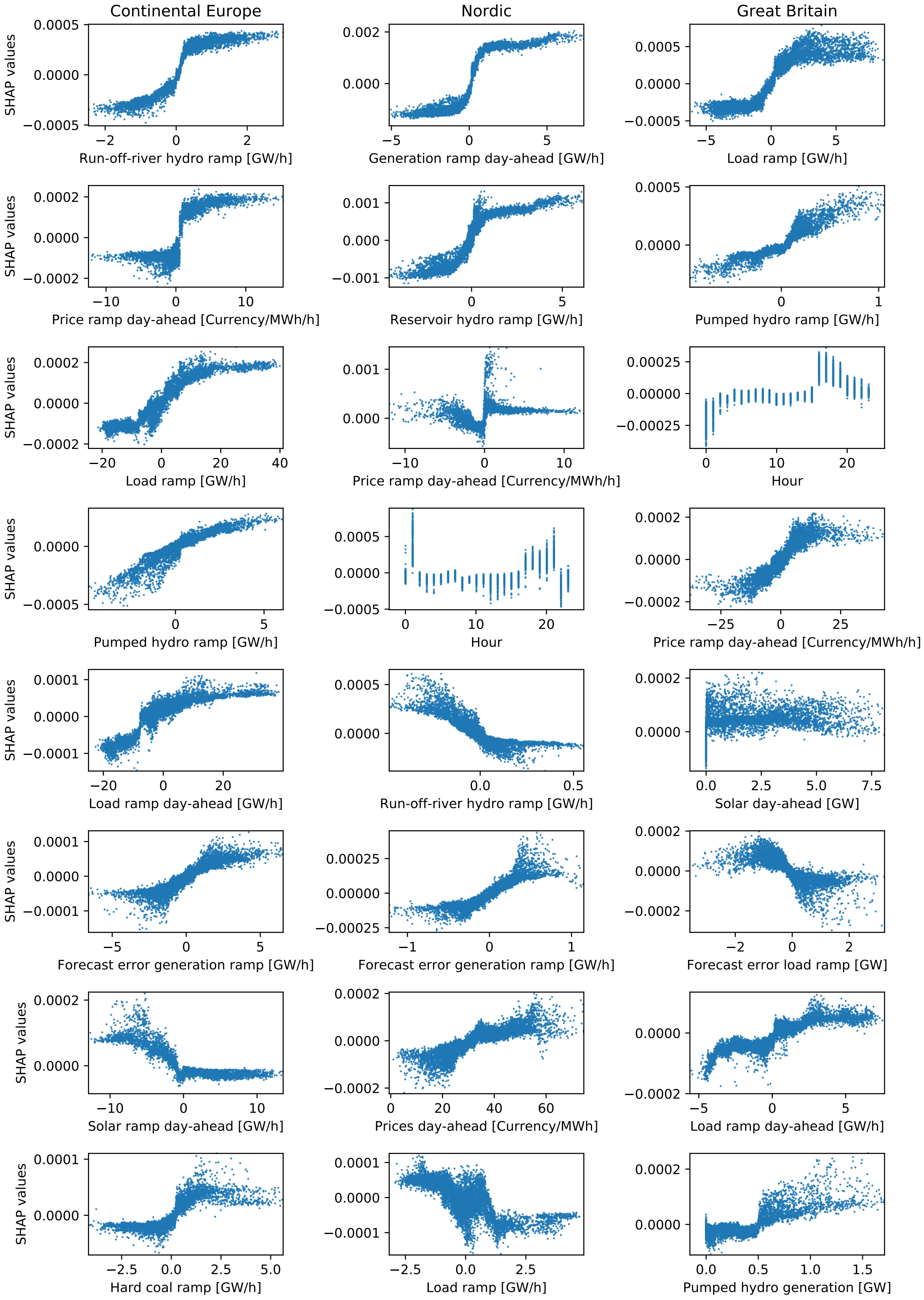

Figure S10. RoCoF dependency plots for the eight most important features.

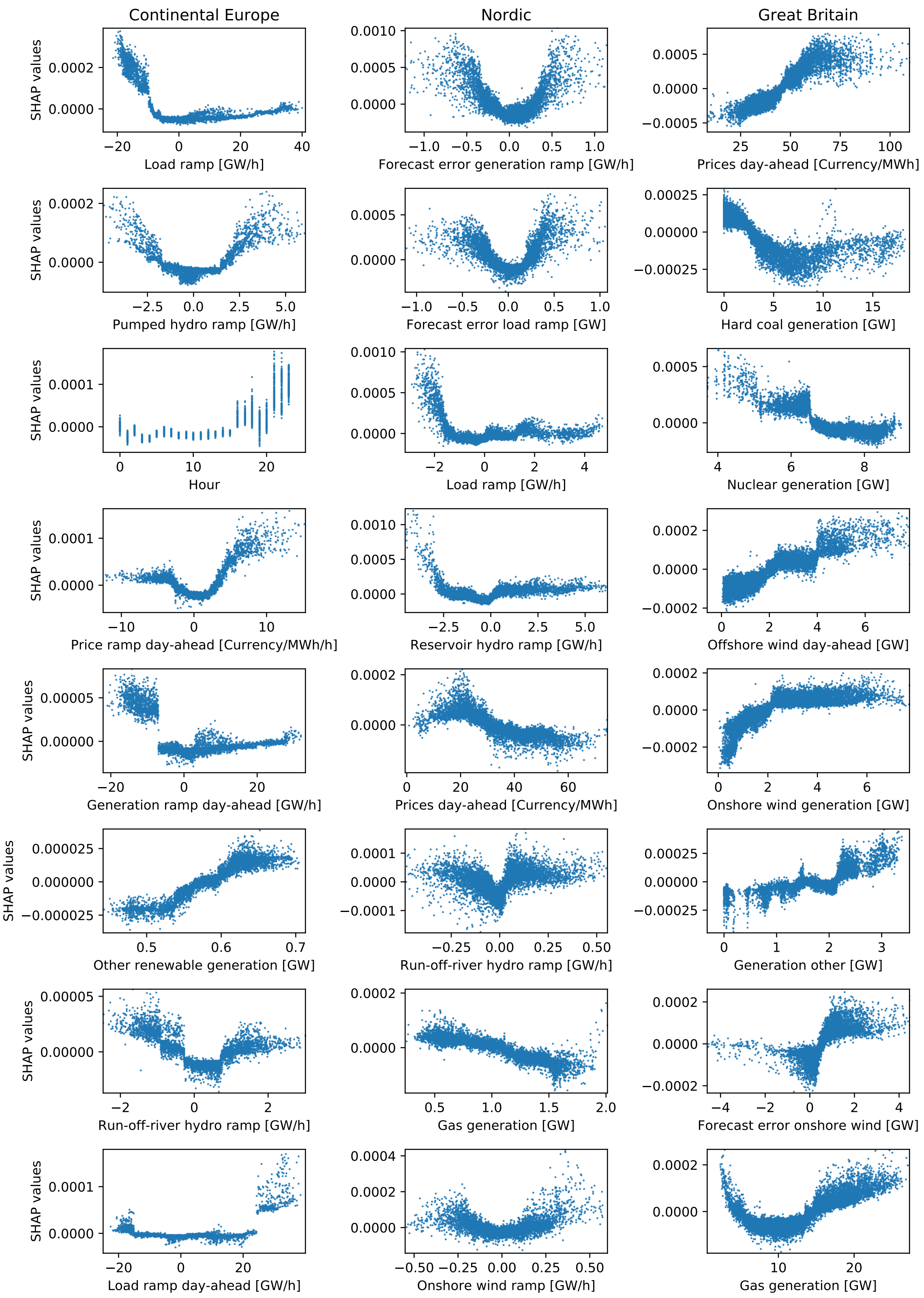

Figure S11. MSD dependency plots for the eight most important features.

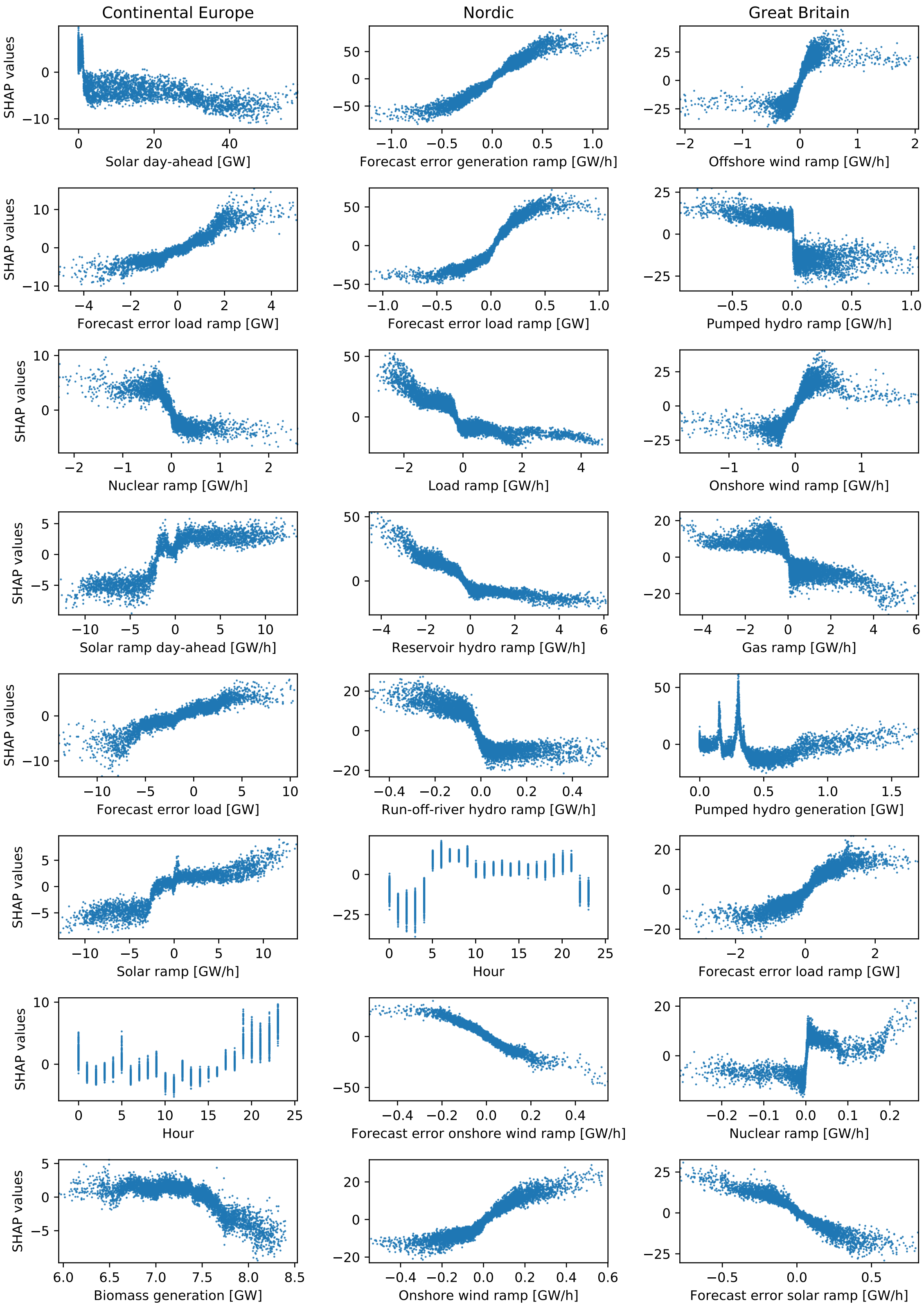

Figure S12. Nadir dependency plots for the eight most important features.

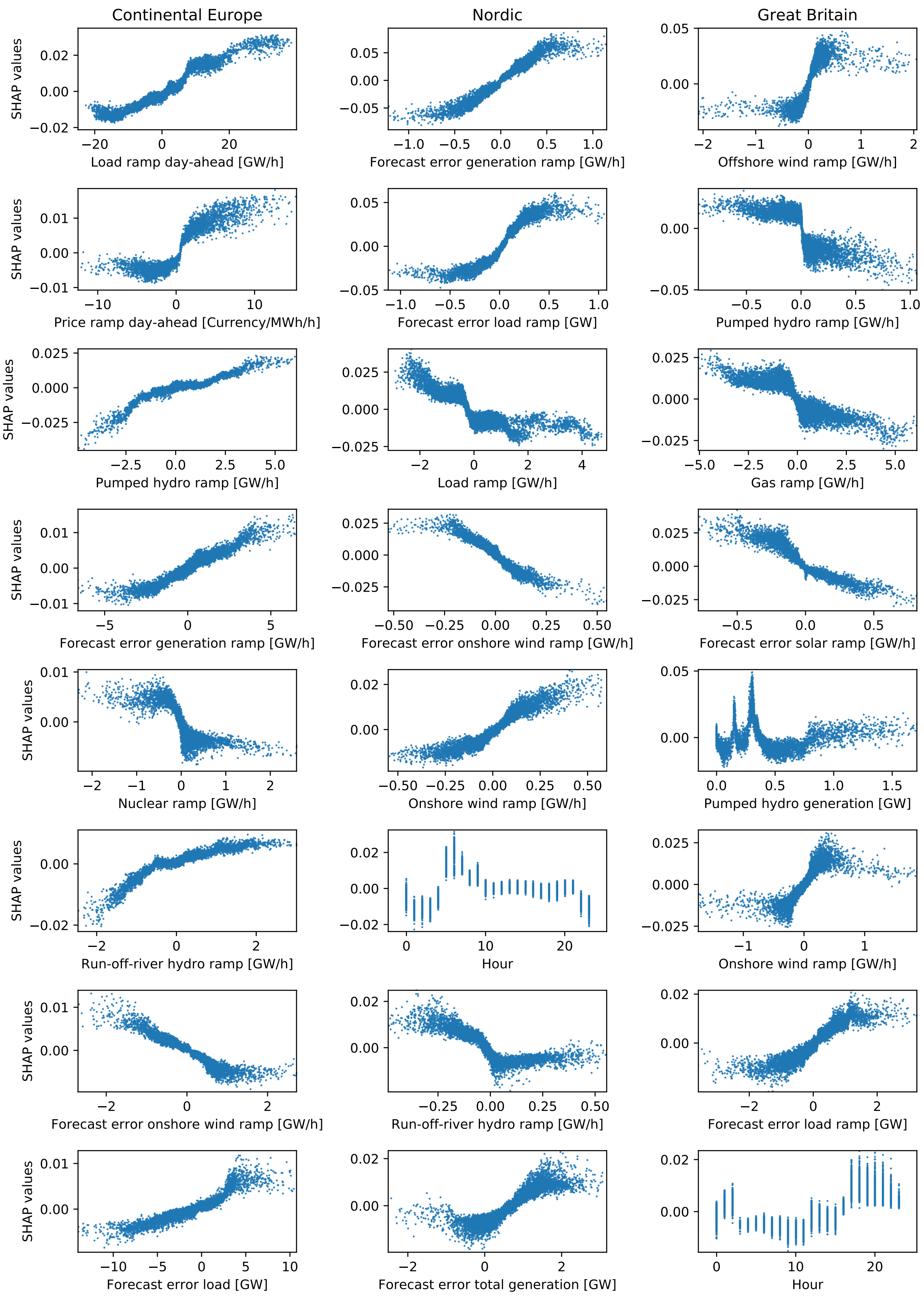

Figure S13. Integral dependency plots for the eight most important features.

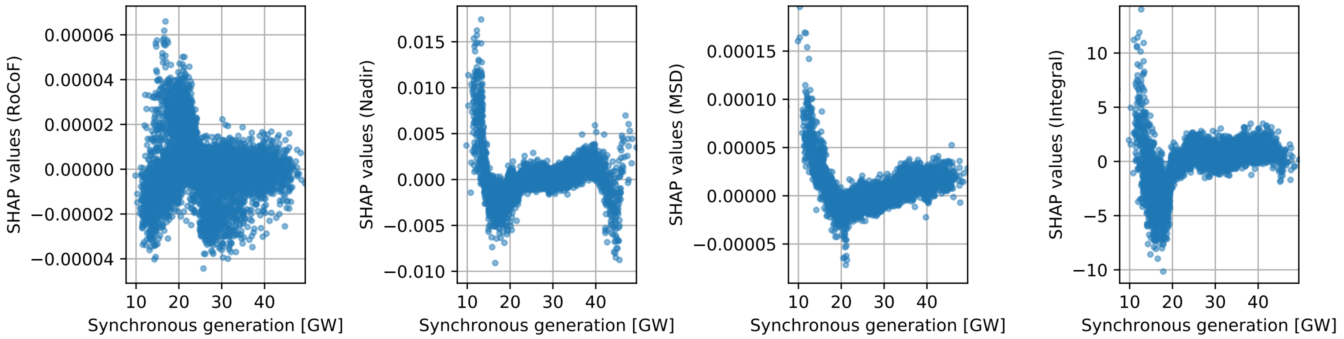

Figure S14. Dependency plots of synchronous generation in Great Britain. Each subplot corresponds to one of our aggregated indicators for frequency stability.

SUPPLEMENTAL REFERENCES

[1] *ENTSO-E Transparency Platform* 2020. https://transparency.entsoe.eu/.

[2] Kruse, J., Schäfer, B. and Witthaut, D. 2021. Supplementary data: "Revealing drivers and risks for power grid frequency stability with explainable AI", https://zenodo.org/record/5118352.

[3] Milano, F., Dörfler, F., Hug, G., Hill, D. J. and Verbič, G. 2018. Foundations and Challenges of Low-Inertia Systems (Invited Paper), *2018 Power Systems Computation Conference (PSCC)*, IEEE, Dublin, pp. 1–25.

[4] Weissbach, T. and Welfonder, E. 2009. High frequency deviations within the European Power System: Origins and proposals for improvement, *2009 IEEE/PES Power Systems Conference and Exposition*, IEEE, Seattle, pp. 1–6.
