# Supplementary material for: Revealing drivers and risks for power grid frequency stability with explainable AI
Source: Patterns (N Y). 2021 Oct 8;2(11):100365. doi: 10.1016/j.patter.2021.100365 (PMC8600233; doi:10.1016/j.patter.2021.100365)
Supplement: Document S2. Article plus supplemental information [file mmc2.pdf]

Article

Patterns

Revealing drivers and risks for power grid frequency stability with explainable AI

Graphical abstract

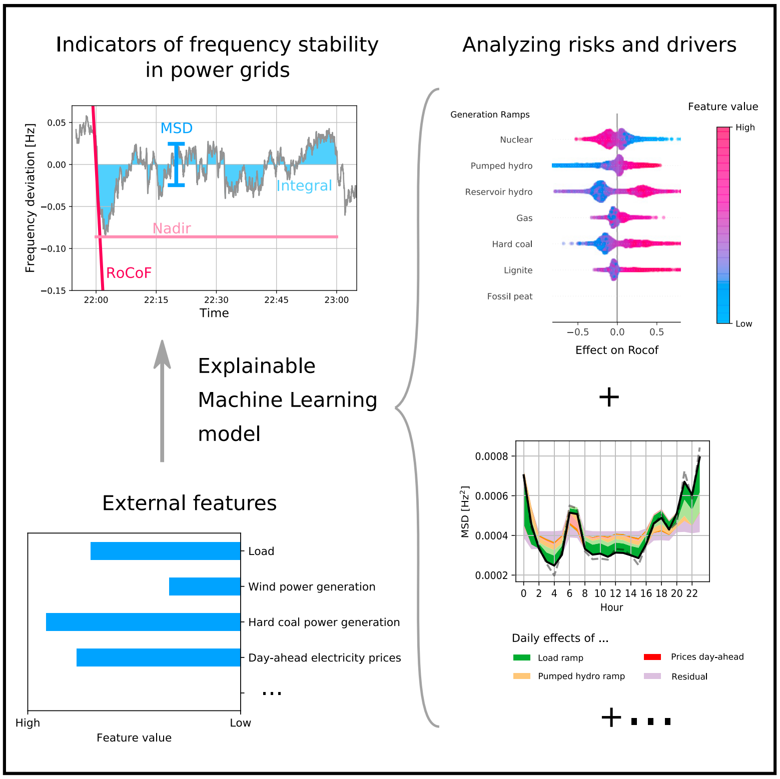

Authors

Johannes Kruse, Benjamin Schäfer, Dirk Witthaut

Correspondence

jo.kruse@fz-juelich.de

In brief

The transition to a sustainable energy system is one of the greatest challenges of our time. With operational data becoming available at an unprecedented level of detail, advanced methods from machine learning must be used to fully harness these data.In our article, we present explainable artificial intelligence (XAI) as a tool to quantify, predict, and explain essential aspects of power system operation and stability in three major European areas, giving indications of how to stably operate future power systems.

Highlights

- Power grid frequency stability is analyzed via explainable artificial intelligence
- Effect of generation ramps differ between Continental Europe, Britain, Nordic grids
- Control efforts are driven by electricity prices and load ramps
- Renewable generation and forecasting errors dominate Britain and Nordic grids

Kruse et al., 2021, Patterns 2, 100365
November 12, 2021 © 2021 The Authors.
https://doi.org/10.1016/j.patter.2021.100365

CellPress

Patterns

CellPress
OPEN ACCESS

Article

Revealing drivers and risks for power grid frequency stability with explainable AI

Johannes Kruse,[1,2,5,*] Benjamin Schäfer,[3,4] and Dirk Witthaut[1,2]
[1]Forschungszentrum Jülich, Institute of Energy and Climate Research - Systems Analysis and Technology Evaluation (IEK-STE), 52425 Jülich, Germany
[2]Institute for Theoretical Physics, University of Cologne, 50937 Köln, Germany
[3]School of Mathematical Sciences, Queen Mary University of London, London E1 4NS, UK
[4]Faculty of Science and Technology, Norwegian University of Life Sciences, 1432 Ås, Norway
[5]Lead contact
*Correspondence: jo.kruse@fz-juelich.de
https://doi.org/10.1016/j.patter.2021.100365

THE BIGGER PICTURE The transition to a sustainable energy system is challenging for the operation and stability of electric power systems as power generation becomes increasingly uncertain, grid loads increase, and their dynamical properties fundamentally change. At the same time, operational data are available at an unprecedented level of detail, enabling new methods of monitoring and control. To fully harness these data, advanced methods from machine learning must be used.

In this paper, we present explainable artificial intelligence (XAI) as a tool to quantify, predict, and explain essential aspects of power system operation and stability in three major European synchronous areas. We focus on the power grid frequency, which measures the balance of generation and load and thus provides the central observable for control and balancing. Combining XAI with domain knowledge, we identify the main drivers and stability risks, while our model and open dataset may enable further XAI research on power systems.

1 2 3 4 5 **Proof-of-Concept:** Data science output has been formulated, implemented, and tested for one domain/problem

SUMMARY

Stable operation of an electric power system requires strict operational limits for the grid frequency. Fluctuations and external impacts can cause large frequency deviations and increased control efforts. Although these complex interdependencies can be modeled using machine learning algorithms, the black box character of many models limits insights and applicability. In this article, we introduce an explainable machine learning model that accurately predicts frequency stability indicators for three European synchronous areas. Using Shapley additive explanations, we identify key features and risk factors for frequency stability. We show how load and generation ramps determine frequency gradients, and we identify three classes of generation technologies with converse impacts. Control efforts vary strongly depending on the grid and time of day and are driven by ramps as well as electricity prices. Notably, renewable power generation is central only in the British grid, while forecasting errors play a major role in the Nordic grid.

INTRODUCTION

The power grid frequency plays a central role for power system control, as it reflects the balance of power generation and demand.[1] An oversupply of power leads to a frequency increase, while a shortage causes a frequency decrease. Large frequency deviations correspond to large power imbalances, which threaten system stability and may lead to large-scale blackouts.[2] Frequency stability is regarded as a major challenge for the transition to a sustainable energy system because renewable power sources do not provide an intrinsic inertia.[3] Understanding the emergence of large frequency deviations is therefore essential.

Deviations from the reference frequency of 50/60 Hz have distinct causes, which are in turn modified by the complex

This is an open access article under the CC BY license (http://creativecommons.org/licenses/by/4.0/).

interplay of different elements of the energy system. For example, changes in power generation due to electricity trading intervals causes regular frequency jumps,[4] the magnitude of which depends on several technical parameters.[3,5] Fluctuating wind and solar power[6,7] or singular load patterns due to societal events[8] create frequency fluctuations on different scales. To guarantee frequency stability in such a complex and uncertain environment, transmission system operators (TSOs) intensively monitor the system and allocate expensive control reserves as necessary. An improved understanding of the frequency dynamics and external influences could greatly facilitate control efforts and contribute to power system stability. While several studies have investigated univariate correlations to quantify the impact of individual features,[9–11] a comprehensive, data-based analysis is lacking.

Modern machine learning (ML) methods are well suited to this task as they can handle a large number of features and large volumes of data. In recent years, the volume of publicly available energy system data has grown steadily, including frequency recordings[12,13] and data on a variety of external features, such as generation and load time series.[14,15] An optimal basis for analyzing and predicting grid frequency with data-driven models therefore already exists.[16] However, complex ML models do not provide insights on the mapping of input to output.[17,18] This is particularly problematic for critical infrastructures such as power systems, where the black box character poses a security risk.[19,20]

Approaches using explainable artificial intelligence (XAI) could change this. XAI is a quickly growing research field, which covers inherently transparent ML models as well as post-modeling explanations for black box models.[21] Shapley additive explanations (SHAP) values are an example of post-modeling explanations, offering a method of measuring feature effects and avoiding inconsistencies present in other approaches.[22,23] In particular, SHAP values have certain desirable properties, such as additivity, efficiency, and symmetry. SHAP values can be quickly computed for gradient boosted trees,[24] which in turn offer a powerful nonlinear modeling and are particularly suited to tabular data. The combination of tree-based models and SHAP values is already widely used, with applications ranging from medicine[25] to geoscience.[26] In contrast, only a few applications of this methodology have been presented in the field of energy systems analysis to date: for example, to explain solar power forecasts,[27] transient security assessments,[28] or power project failures.[29]

Here, we present an explainable ML model based on gradient boosted trees for selected indicators of frequency stability, and we evaluate its predictive power for three grids in Europe: Continental Europe (CE), the Nordic area, and Great Britain (GB). We demonstrate the benefits of explainability via SHAP values, ranging from coarse-grained global feature importances to detailed dependencies and finally to fine-grained interactions between different external features. In particular, we quantify the impacts of generation and load ramps on the rate of change of frequency (RoCoF) at the beginning of each hour. SHAP values explain the different impacts and roles of different generation technologies. We use aggregated SHAP values to analyze efforts to control generation, which vary strongly depending on the grid and time of the day. We then investigate enduring frequency deviations, which can be attributed to systemic power imbalances, and discuss the role of solar power generation. As data, we utilize the hourly time series of four stability indicators (model outputs or targets) and 66 external features (model inputs) for the years 2015–2019 (see also our Zenodo[30] repository).

Our approach complements established simulation-based methods that predict frequency deviations on the basis of load and generation forecasts. Although simulations can be very accurate, they are reliant on the quality of input data, underlying forecasts, and specific parameters. Data-driven models can reveal additional driving factors, unknown effects, and emerging risks and thus complement and improve existing simulations. For instance, our analysis highlights the role of forecasting errors, which varies depending on the grid.

The next two sections of this paper present the four frequency stability indicators and our ML model. Then, the most important features in each synchronous area are identified before the influence on generator ramps—in particular, on RoCoF predictions—are discussed and nonlinear feature dependencies are revealed. We go on to demonstrate how SHAP analysis reveals feature interactions before concluding with a discussion.

RESULTS

Frequency stability: Indicators and influences

The power grid frequency fluctuates on various timescales, ranging from seconds to weeks.[31] In our model, we aggregated frequency deviations to hourly indicators, which are directly relevant for power system stability (Figure 1; experimental procedures). We analyzed the maximum frequency deviation within the hour (nadir)[32] and the RoCoF,[32] which are of central relevance for grid monitoring and control. Nadirs above a threshold level indicate immediate danger and can be counteracted with measures such as load shedding. High RoCoFs are dangerous because control actions require a few seconds to take effect. In addition, we evaluated two integrated stability measures to account for the duration of frequency deviations. We characterized the variability of hourly time series using the mean square deviation (MSD) from 50 Hz. The MSD also indicates the total (primary) control effort, meaning that a large MSD reflects high operational costs.[33] Finally, we evaluated the integrated frequency deviation (integral), which is proportional to the mean deviation within the hour. Large integrals correspond to a systematic imbalance between the hourly power generation and the demand. Regional differences in the grid frequency within a synchronous area are small during normal operation and are typically damped out after several seconds.[34,35] Although we used local grid frequency measurements, the above indicators characterize frequency stability in an entire synchronous area.

We evaluated these four indicators on an hourly basis, as this timescale is central for power system operation.[37] Electricity is traded predominantly in blocks of one hour, and generation is adapted at the beginning of each hour, leading to deterministic patterns in frequency.[4] When the load decreases continuously during an hour, but the dispatch is set to the hourly mean, then power is scarce at the beginning of the hour and the frequency drops. As a consequence, frequency deviations show a pronounced daily profile, which we use later as a null model to evaluate prediction performance. Another reason for choosing to

CellPress
OPEN ACCESS

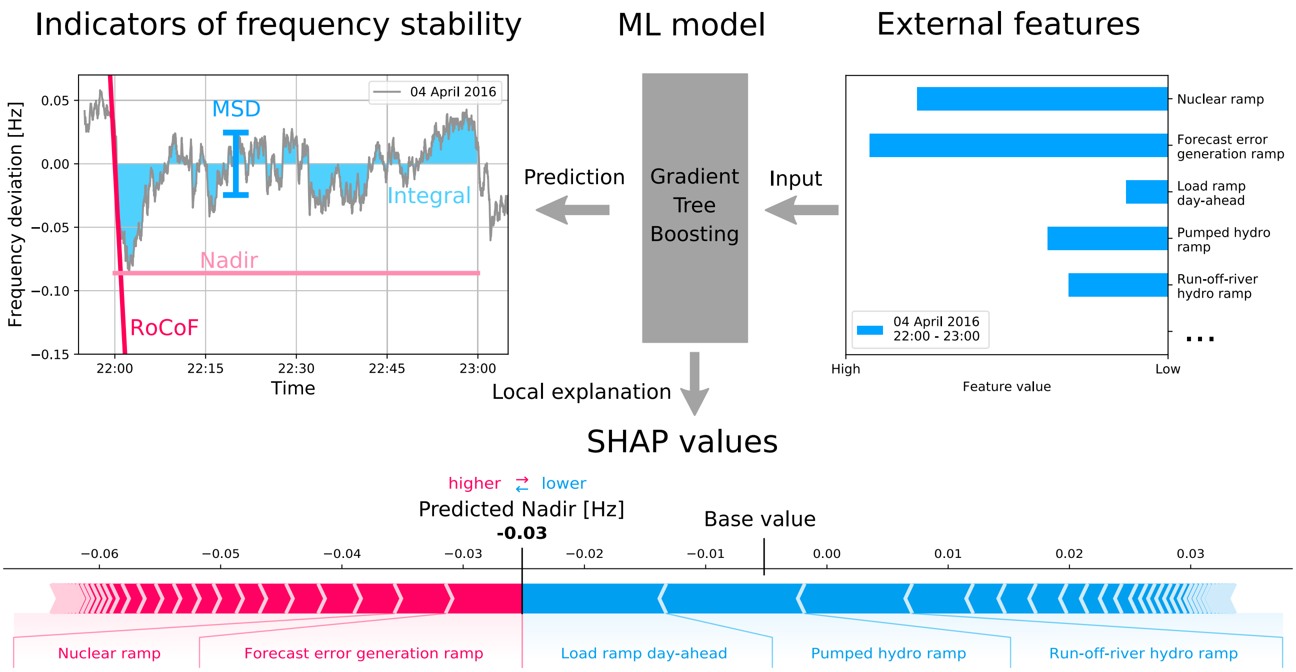

Figure 1. Overview of our explainable ML model

From right to left: using publicly available external features from the ENTSO-E transparency platform,[36] such as load ramps or generation ramps, a gradient tree boosting ML model was constructed to predict indicators of frequency stability. The model was then interpreted using SHAP values, which quantify the effect of the features on the model output in relation to a base value (see experimental procedures). The SHAP value of each feature is shown at the bottom of the figure, separated by white arrows; only the most important features are named. Together with the base value, positive (red) and negative (blue) SHAP values add up to the model prediction. The data represent a sample hour in 2016 from Continental Europe.

evaluate on an hourly basis is that most external features are only publicly available at an hourly resolution.[14]

The assessment of frequency stability indicators conventionally focuses on the transient response after a major disturbance.[1] Many model-based simulation studies have investigated the effects of various parameters on the frequency response, in particular the effect of inertia,[38] as well as effects on the properties of the load-frequency control system.[10] In recent years, ambient and deterministic frequency fluctuations have received more attention in the context of model-based simulations. Studies have highlighted the influence of inertia, control system parameters,[5] and intermittent wind power feed-in[39] on the frequency statistics. Deterministic frequency deviations (DFDs) have been studied using dynamical models[4] and stochastic models[40] revealing the importance of the daily load evolution and generation jumps caused by electricity trading. The main limitation of the simulation approach is that data and parameters are often not publicly available to accurately model all interactions within the power system. For example, load-frequency control systems are operated by individual TSOs, and parameters may have been disclosed to other TSOs.[41] Thus, simplified assumptions are used, which often do not reflect effects present in real-world data.

Over the last few years, comprehensive datasets have become publicly available, enabling an *empirical analysis* of power system frequency stability.[13,35] Most data-driven studies focus on the impact of a single isolated feature and resort to a linear correlation analysis. For instance, studies have quantified the correlations between different measures of frequency quality and the load value and ramps in the Nordic grid,[11] wind power generation in the Irish grid,[9] load ramps in the British grid,[10] and societal events coinciding with large frequency deviations.[8] A correlation between load and solar ramps as well as trading volumes reflects the role of solar power in power balancing.[42] The relation between wind power generation and large frequency increments in the CE grid has been studied using conditional probabilities by Haehne et al.[7] Although existing studies provide us with essential insights into power system operation and frequency stability, they are limited in two ways. Firstly, linear correlation analyses cannot capture nonlinear dependencies and may thus underestimate or even overlook important effects. Secondly, only one feature/covariate is used in most cases, and the observed effects are not adjusted for other variables. This is problematic when features are correlated, e.g. due to confounding. Modern ML methods can capture multiple dependencies and thus provide more accurate results.[16]

An explainable model for frequency deviations

We developed an explainable ML model to predict indicators of frequency stability from external features (Figure 1; experimental procedures). We used gradient tree boosting (GTB), which produces nonlinear models with state-of-the-art performance for many ML applications[43] while enabling a fast and efficient calculation of SHAP values to explain the predictions.[24] We fed our model with physically meaningful features based on load, generation, and electricity price time series. Our data included both

day-ahead available features, such as the day-ahead predicted load change ("load ramp day-ahead") and ex post available features, such as the error between the day-ahead forecast and the actual total generation change ("forecast error generation ramp"). Finally, we computed SHAP values to quantify how each feature contributes to the model output. For example, in Figure 1 (bottom), the feature "load ramp day-ahead" has a negative contribution (blue), thus causing the predicted nadir to be lower than its average. SHAP values make local predictions more transparent and enable aggregated insights into global feature effects, dependencies, and interactions. However, it should be noted that SHAP values do not guarantee causal relations (see experimental procedures for a more detailed discussion).

Based on its R^2 score, our model outperformed the daily average profile of the stability indicators, which is an important system-specific null model (experimental procedures and supplemental experimental procedures S5). We achieved performances 3.7 (CE), 7.6 (Nordic), and 16.3 (GB) times higher than the daily profile, thus indicating additional important dependencies. Restricting the full model to day-ahead available features resulted in similar performance gains of 2.6 (CE), 3.0 (Nordic), and 8.9 (GB), which opens the possibility of predicting stability indicators a day ahead. The ability to include ex post available features, such as forecast errors, was particularly beneficial in the Nordic area. Here, the full model performed 2.6 times better than the restricted day-ahead model. This indicates the importance of forecast errors for the Nordic frequency dynamics, which we examine in the next section.

Main features affecting frequency deviations

We demonstrated our model explainability on the coarse-grained level of global feature importances, which characterize how much a certain feature affects the hourly frequency stability indicators within the trained model (Figure 2).

In the RoCoF model, only a few features dominated: mainly generation ramps from hydropower and load ramps. The importance of hydropower generation ramps relates to their large ramping speed, which we discuss below. In the Nordic area, the total day-ahead generation ramp is much more important than load ramps for the RoCoF. This suggests that changes in power export and storage may be relevant here, as these are not represented in the load for the area.

The nadir was primarily affected by ramps and their respective forecasting errors. In CE, the day-ahead load ramp was the most important feature. This reflects the importance of DFDs, which are strongly correlated to the direction of the load ramps.[4] In the Nordic grid, the forecast errors of generation and load ramp were by far the most important features, partly explaining the large performance gain when ex post data were included in the model (see above). In contrast, there were many features of almost equal importance in the British nadir model. Here, wind power ramps and solar ramp forecast errors were among the five most important features. This indicates the importance of renewables for frequency deviations in GB.

The MSD behaved similarly to the nadir in CE and in the Nordic grid, with some subtle differences. Load ramps were the most important feature in CE. Forecasting errors again dominated in the Nordic grid, but load and hydropower generation ramps also played a role. A different situation was found in GB. Day-ahead prices dominated the MSD prediction, with some generation forms (coal and nuclear) coming in at a distant second, while generation ramps did not significantly contribute. These differences point to a more complex behavior of the MSD, which we further discuss below.

Finally, the integral was largely affected by forecasting errors for load and generation ramps, which caused long-lasting power mismatches. This was particularly evident in the Nordic grid, where other features were not as important. In GB, wind power ramps were ranked highly, confirming the importance of renewables. In CE, solar power generation and ramps, as well as nuclear power ramps, were relevant for the prediction. We investigated how the interaction of these two distinct generation types explain model variance.

In summary, CE exhibited strong DFDs that were connected to hourly load and generation ramps. This is consistent with previous results[4,41] (Figure S10). Nordic frequency deviations were strongly connected to forecasting errors, which is in line with Nordic TSOs reporting forecast errors as a driver.[44] In GB, hourly DFDs were less important (supplemental experimental procedures S4) and frequency deviations were mainly affected by renewables, i.e. their fluctuations and forecast errors (cf. National-grid ESO[45]). The total synchronous generation, which approximates the inertia in our model (experimental procedures), affected the British frequency dynamics only in extreme situations where there was very low inertia (supplemental experimental procedures S6). Despite the importance of reduced inertia in renewable energy systems,[3,45] our model showed that the average effect of inertia on the aggregated stability indicators is negligible (Figure 2). This was consistent with other studies on aggregated frequency fluctuations (cf. Vorobev et al.[5]), which found that inertia is important for extreme events but aggregated dynamics are not. It should be noted that we focused on frequent daily fluctuations and stability concerns, which are highly relevant for TSOs and for reducing daily operational costs.[33] This supplements studies focusing on blackouts and cascading failures.[46]

Characterizing the effect of generation ramps

Fast generation ramps significantly affect the hourly RoCoF. For this reason, we went beyond mean feature importances and examined the direction of these dependencies using SHAP values (Figure 3). The effect of ramps is mostly monotonic, meaning that a feature effect either increases or decreases monotonically with the feature value (Figures 3A–3C). Remarkably, the direction of the dependency varies depending on the type of generation and the grid. As expected, hydropower generation ramps were consistently positively correlated (see Figure 3D for CE). The dependency of hard coal ramps for CE was the opposite to the dependency for GB and the Nordic grid.

The observed differences between the generation types can be explained in terms of the *relative* ramping speed of a generation type within a respective area (see experimental procedures on how this speed is estimated). In the Nordic grid, hydropower, a technology capable of fast ramps, is essential and all other generation types must be considered slow in comparison. In GB and CE, non-hydropower dominates the generation mix and technologies with slower ramps than hydropower plants

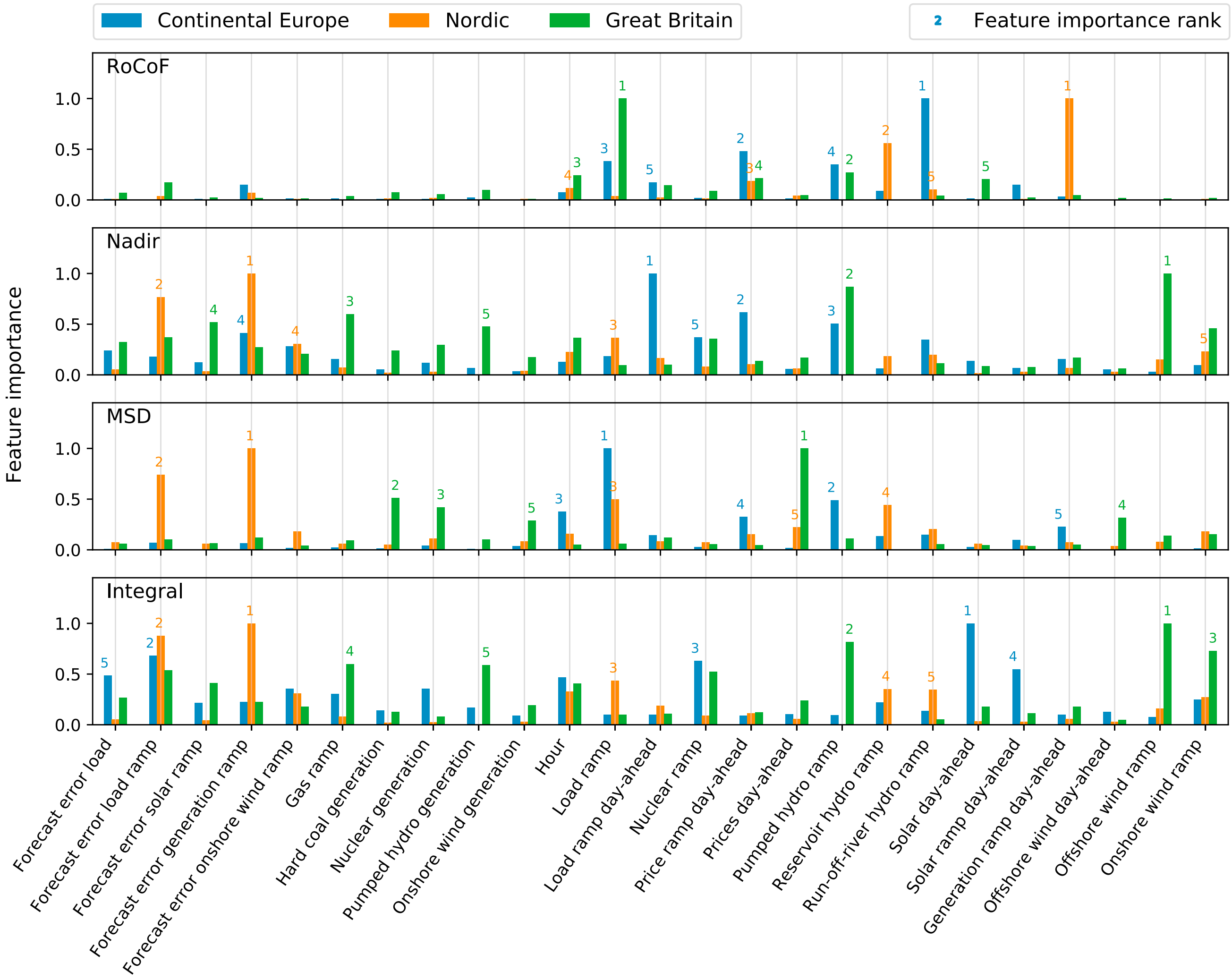

Figure 2. Most important features for predicting frequency stability

The feature importance in our model is measured by the mean absolute SHAP value. The union of the five most important features for each stability indicator and area is shown (see experimental procedures). The importance rank of the five most important features for each area is given above the corresponding bar. While forecast errors and load and generation ramps have a high relative importance, the total synchronous generation is not among the five most important features and its average effect is therefore negligible.

but ramps faster than other generation types play important roles. Notably, hard coal is one of the slow generation types in GB but one of the fast types in CE due to the importance of nuclear and lignite generation in CE, which are even slower than coal. We categorized the generation types using SHAP values for the generation ramps to predict the RoCoF and relative ramping speeds (Figures 3D and 3E). We found that fast generation ramps drove the RoCoF. A positive ramp was associated with more positive frequency jumps. In contrast, ramps of slow generation types offset the RoCoF, leading to a negative correlation. The only exception here was the behavior of gas power plants in GB, which showed a negative correlation despite being fast. This is due to their role as the prime source of balancing reserve in GB.[47] To summarize, the ramps no longer drove the RoCoF, but the RoCoF drove the ramps.

Notably, a model-agnostic data analysis does not produce such consistent results, as our features are strongly correlated (experimental procedures). For example, the Pearson correlation coefficient between nuclear ramps and RoCoFs in CE is positive (supplemental experimental procedures S3). Instead, the SHAP framework indicates a negative relationship, which we consistently explain with relative ramping speeds.

Relating large control efforts to nonlinear dependencies

Frequency stability indicators often exhibit a complex nonlinear dependency on the features. Using the MSD, an indicator for the (primary) control effort,[33] as an example, we found that the daily profiles of the MSD differed strongly between the three grids (Figure 4). These differences were well reproduced by the ML model and were explained using daily aggregated SHAP values (experimental procedures).

In CE, the control effort peaked around midnight (Figure 4A) due to the nonlinear effects of negative load ramps. Details on this relation are shown in a dependency plot (Figure 4D). Load

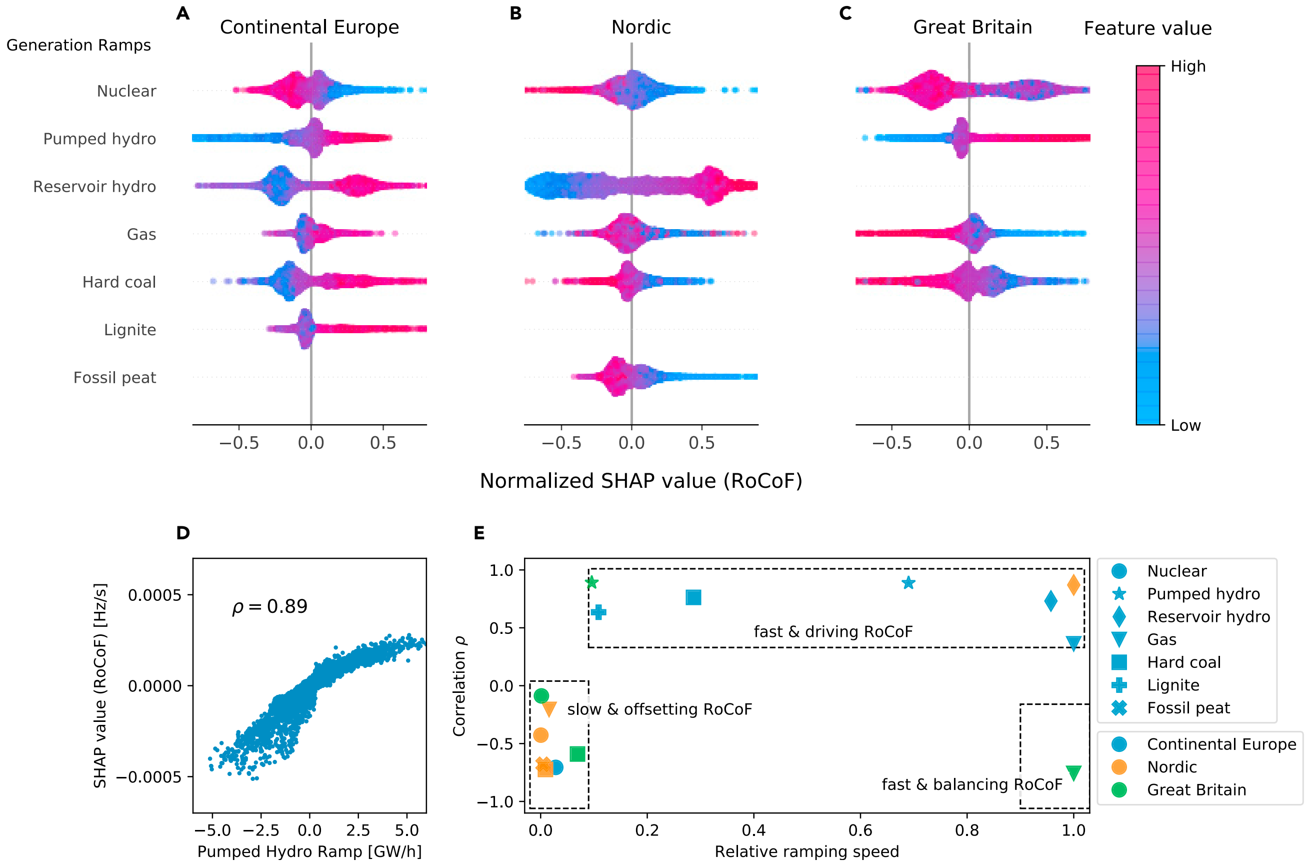

Figure 3. Effect of generation ramps on the RoCoF

(A–C) Examination of the effects of dispatchable, i.e., weather-independent, generation technologies, which generally affect the hourly RoCoF due to their gradual change at the beginning of (hourly) trading intervals.[4] The bee swarm chart depicts the SHAP effects on the RoCoF in the Continental Europe (A), the Nordic (B), and the Great Britain (C) grid areas. For each area and generation type, we normalized the SHAP values by their maximum absolute value to improve visibility. Each colored dot represents one time step in the dataset and the dots pile up vertically to indicate their density on the x axis. The figure only examines generation ramps with a feature importance higher than 0.01 to ensure reliability of results.

(D) Quantification of the direction of the dependencies with the correlation between the feature value and the SHAP effect, shown here for pumped hydro ramps.

(E) Combining the directions with the relative ramping speeds of the generation technologies (see experimental procedures) helps to distinguish RoCoF-driving and RoCoF-offsetting technologies within the three areas.

ramps between −7 and +25 GW/h had a small negative effect on the MSD because such small ramps are easy to control. Outside this range, the effect increased strongly in a nonlinear and asymmetric manner. Negative load ramps had much larger effects than positive load ramps, and they occurred almost exclusively around midnight (see color code). In the Nordic daily profile, load ramps were also the most important feature (Figure 4B), and they showed a very similar nonlinear dependency (Figure 4E). In contrast, the daily MSD profile in GB strongly depended on day-ahead prices (Figure 4C), which had an almost linear dependency (Figure 4F). The control effort peaked during the day in response to high prices in the day-ahead market, while the MSD and the prices were low at night (00:00 to 04:00 h).

Notably, fluctuating renewables did not contribute strongly to the daily MSD profile in our model, although they are an important driver for frequency fluctuations in GB in general (cf. Figure 2). The observed differences between the synchronous areas could be due to different control regulations. For example, in GB, wind power farms must provide frequency control,[48] and secondary control is allocated manually.[49]

Explaining systematic imbalances with interactions

The SHAP framework explains the role of different features and reveals how predictions depend on the *interaction* of features (see Figure 5 for an application of the integral in the CE grid and experimental procedures for technical details). It should be noted that the ML predictors for the other targets also displayed clear interactions. The most important features were solar and nuclear power ramps, which had a reverse dependency (Figure S13). Without interactions, the SHAP value increased gradually and nonlinearly with the solar ramp (Figure 5B). Strong negative ramps of solar power generation induced an ongoing shortage of power and thus led to negative integrals.

Interactions with nuclear and gas ramps altered the effect of solar ramps by up to 60%, leading to a strong vertical dispersion of the observed SHAP values (Figure 5A). In particular, negative nuclear ramps amplified the effect of negative solar ramps, while

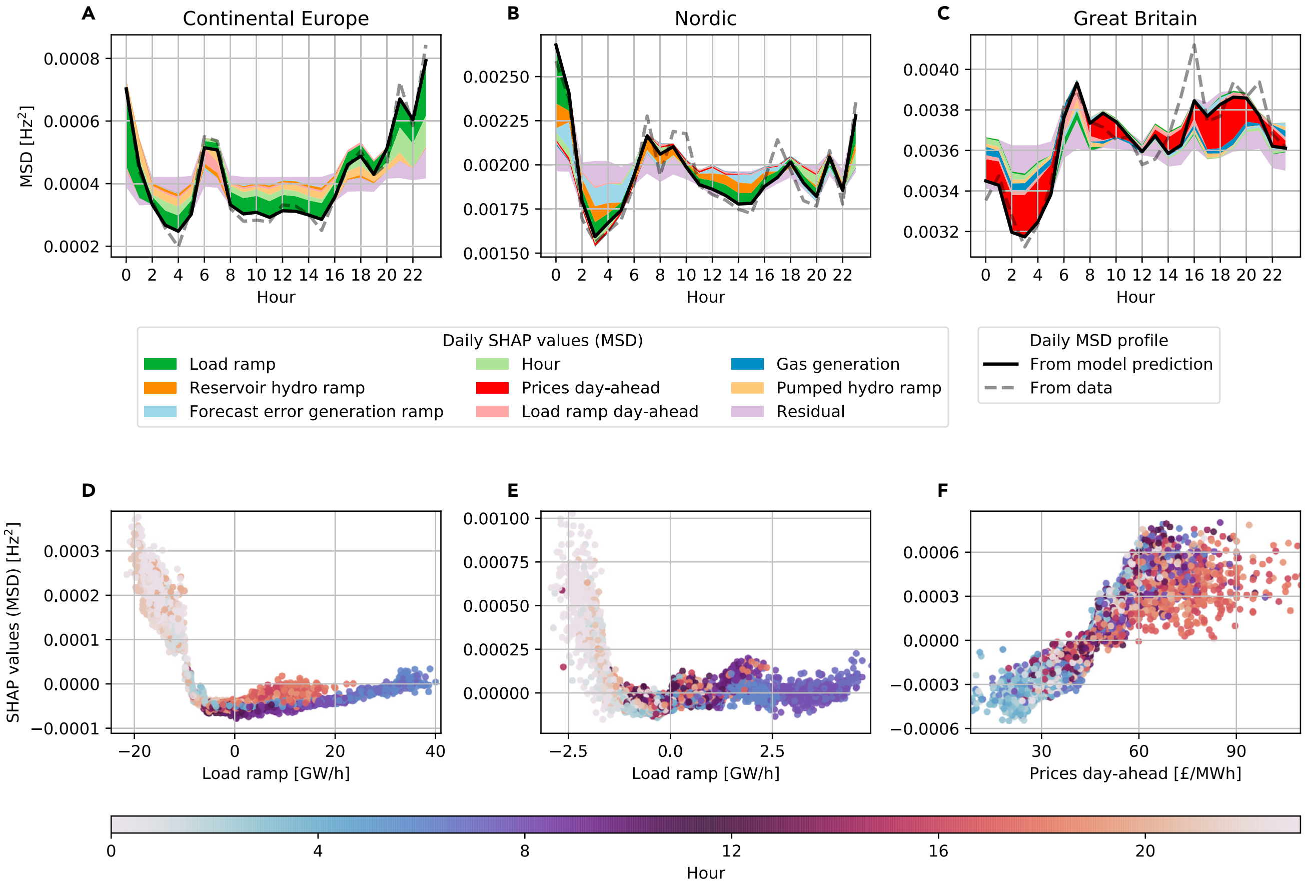

Figure 4. Explaining the daily average control effort with SHAP values

(A–C) The daily average profile of the MSD (dashed line), i.e., the daily average control effort, is very well reproduced by the ML model (solid line), but its shape differs between the areas. We examine these differences with daily SHAP values for the MSD in Continental Europe (A), the Nordic grid (B), and Great Britain (C). Daily SHAP values (see experimental procedures) are sorted such that negative effects are plotted above the prediction line and positive effects below it. The importance of the plotted feature effect decreases the farther away the feature is from the prediction line. Less important features are aggregated in a residual variable.

(D–F) For the Continental Europe (D) and Nordic (E) grids, the dependency plots of the most important daily SHAP effects reveal nonlinear relationships. These relationships explain the large control effort around midnight (color code), while the linear effect of prices explains the low control effort in GB during the night (F).

negative gas ramps dampened the effect on the integral (Figures 5C and 5D). These opposite interactions were again related to different ramping speeds. Nuclear power has the lowest ramping speed in CE, which meant that negative nuclear ramps amplified the continuous ramping behavior in interaction with solar ramps. In contrast, gas power had a fast ramp and therefore often provided balancing power, leading to the opposite effect. In general, these results demonstrate that interactions can influence how strongly a single feature affects power system stability.

DISCUSSION

Our model is based on explainable ML, and it predicts important indicators for power system frequency stability using external features, such as day-ahead electricity prices or total system load. Using real data (ex post analysis), our ML model outperformed the daily profile, a system-specific null model, by a factor of up to 16.3. Using only day-ahead available data, our ML models performed similarly in most cases. When SHAP values were calculated and examined, our model revealed important features and dependencies, and could thus pave the way for multiple applications.

Our model offers a versatile and substantially improved approach for analyzing risks and drivers of grid frequency stability. Previous data-driven studies analyzed the impact of one external feature on grid frequency fluctuations based on linear correlations[9–11] or conditional probabilities.[7] Such univariate analyses cannot be adjusted for effects of other features, which could be correlated with the feature of interest and may lead to incorrect conclusions (cf. Weißbach et al.[42]). Moreover, univariate, linear dependency analyses underestimate the effects of nonlinearities and ignore feature interactions. Our model includes multiple variables and fits nonlinear dependencies and interactions, which are made transparent by SHAP values. It breaks down the effect of correlated features (as discussed in Figure 3) and reveals otherwise undetectable nonlinear effects (Figure 4) and feature interactions (Figure 5). In addition, our model visualizes feature effects in the daily average evolution of frequency stability (Figure 4), which adds to the many useful visualization tools available

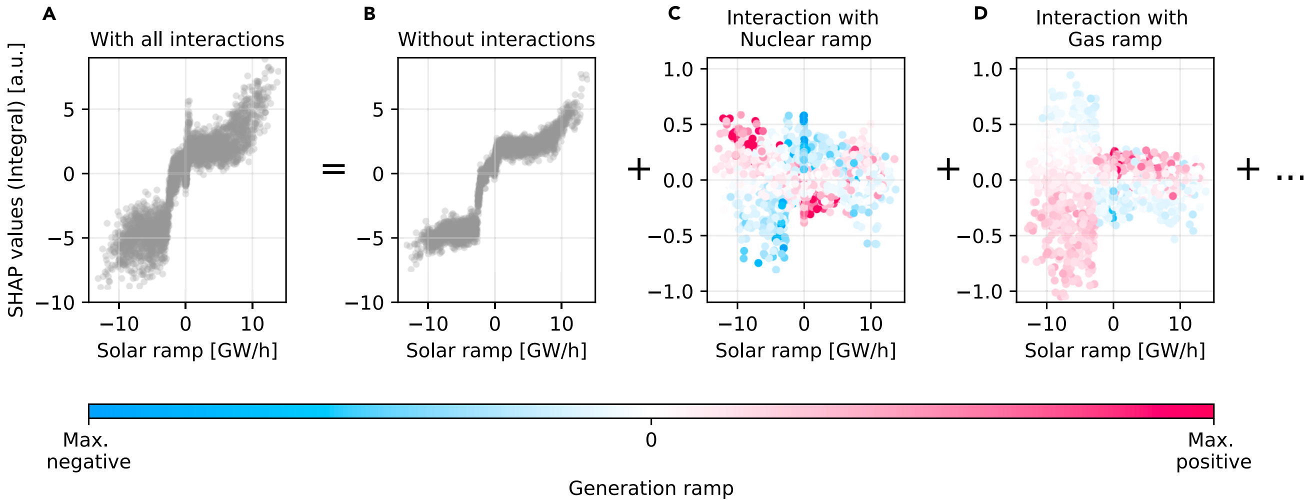

Figure 5. The effect of feature interactions on systematic power imbalances

(A) CE is used to show the SHAP effects of solar ramps on the frequency integral, which are the most important effects in CE (Figure 2). The integral, which represents systematic imbalances, decreases for negative solar ramps, but the SHAP effects vary strongly, as indicated by their vertical dispersion.

(B–D) Using SHAP interaction values (see experimental procedures), this dispersion was broken down into different interaction effects. These effects depend on the generation type, as negative nuclear ramps weaken the effect of negative solar ramps (C), while negative gas ramps lead to an amplified effect (D). Subtracting all interaction effects from the original SHAP values (A) yields the remaining effect of solar ramps (B), which is strongly altered.

in the SHAP framework. Our publicly available model[50] and dataset[30] can be used to predict any frequency stability indicator, thus offering a ready-to-use and flexible tool for analyzing power grid stability.

We applied our model to three different synchronous areas and identified options for improving power grid operation strategies. We discussed four examples for potential applications: (1) first, we showed how generation ramps drove the RoCoF and the nadir and revealed subtle differences between generation types and grids. These insights can help to optimize ramping behavior and mitigate DFDs[51] and improve frequency quality. In particular, hydropower generation ramps should be optimized in response to other ramps. (2) Our results show that forecasting errors play an essential role in the Nordic grid. While TSOs are generally aware of the problem,[44] the SHAP analysis provides a much more detailed view and reveals when and how these errors affect frequency stability. An example is given in Figure 4B, which shows that the features “forecasting errors of the generation ramps” are particularly important during the night. Our model identifies situations where forecasting errors are particularly critical and will thus improve risk awareness in grid operation. (3) Low inertia has been identified as a major threat for the stability of future power grids.[3] Our analysis provides a more finely nuanced view on this topic. In our XAI model, inertia does not generally have a high feature importance. Instead, the impact of inertia on frequency stability is nonlinear and more pronounced for low inertia values (Figure S14). (4) Finally, the predictive power of our XAI model can be harnessed for online grid monitoring and preventive control measures. For instance, a model-predictive frequency restoration reserve has been proposed to mitigate DFDs.[52] The applicability of such predictive control strategies can be extended by data-enabled methods (cf., e.g., Huang et al.[53]).

The main restrictions to our model performance and explainability are due to the quality of available power system data. Firstly, frequency deviations due to renewable fluctuations[7] or load fluctuations[8] occur on timescales that are smaller than the intervals of electricity trading. The limited time resolution of publicly available power system time series restricts both the performance of an ML model and its ability to suggest causal relationships because the time order of events is partly hidden. Secondly, all locations in a synchronous power grid affect the frequency deviations; but in large grid areas, such as CE, many countries provide no or only a limited amount of data.[14] This further emphasizes the need for open data in energy system analysis and design.[54]

Our paper contributes to the applicability of XAI methods in energy systems and engineering sciences in general. Firstly, deriving causal relationships from data is a key challenge in modern ML techniques. With the power grid frequency, we provide a very well-suited test bed; while not all features and interactions are available, there is plenty of domain knowledge to interpret and cross-check XAI results. Secondly, we provide an excellent dataset[30] for applications and the benchmarking of ML methods, such as causal inference or predictive models. While generation data are already publicly available,[36] aggregating these for a whole synchronous area and combining them with frequency stability indicators yields a novel dataset for future usage. Finally, in Figure 4, we explored how daily aggregated SHAP data may be used to explain specific temporal profiles, which could be useful in other ML applications when dealing with strong daily or seasonal trends, e.g., in weather or traffic predictions.[55,56]

In conclusion, we hope that our work will trigger further applications of XAI in energy science, harnessing the strengths of modern ML tools while avoiding the drawbacks of black box approaches, which impede scientific insights[18] and pose security risks.[19] Our model provides insights by explaining feature effects

with SHAP values in the context of the domain science. SHAP dependency and interaction plots visualize the knowledge learned by the model and offer individual explanations for each prediction. The most predictive associations then suggest causal relationships, which can then be validated by domain knowledge or further experiments. For example, we identify RoCoF-driving, RoCoF-offsetting, and RoCoF-balancing generation technologies by connecting our model results to physical ramping rates, thus suggesting different causal relationships. "Suggesting relations" is key here since neither boosted trees nor SHAP guarantee causal relationships but rather indicate associations based on the data. All in all, SHAP values alone do not provide scientific insights, but, when combined with domain knowledge, they can lead to further knowledge.

Future work includes explicitly forecasting the given indicators and classifying whether upcoming events could be problematic for grid operation. Once forecasts or other early warning and control methods have been implemented, our model will need to be retrained using these new, controlled datasets to derive the updated feature-target interactions. Furthermore, while we already outperform the daily profile, the performance of our tree-based predictor could be improved if further features were integrated and our model was compared with other ML prediction models. Finally, such regression models should be complemented by causal inference models to provide clear counterfactual statements and comparisons with XAI approaches.

EXPERIMENTAL PROCEDURES

Resource availability

Lead contact

Further information questions should be directed to the lead author, Johannes Kruse (jo.kruse@fz-juelich.de).

Materials availability

This study did not generate new unique materials.

Data and code availability

The dataset to reproduce our results is available on Zenodo: https://doi.org/10.5281/zenodo.5118352. The Python code used to create our results and the figures is also archived on Zenodo: https://doi.org/10.5281/zenodo.5497609.

Data preparation of frequency stability indicators

In a modern AC power grid, the grid frequency is typically spatially synchronized and its dynamics can be represented by a single bulk time series on timescales of several seconds and more.[1] In Europe, different synchronous areas exist, which are only inter-connected through DC links and hence display their own frequency dynamics and follow their own specific regulations. We modeled the bulk frequency dynamics for different synchronous areas in Europe, specifically for the CE, Nordic, and GB areas. We used pre-processed frequency time series $\tilde{f}(t)$ with a time resolution of $\tau = 1$ s,[13] which were originally measured by regional TSOs.[57–59]

From the centered frequency time series $f(t) = \tilde{f}(t) - 50$ Hz, we extracted four hourly stability indicators, which are directly relevant for power system operation.[32,33] For the ith hour starting at time t_i, we calculated the (positive or negative) nadir, the integral and the MSD based on the hourly time steps $I_i = \{t_i, t_i + \tau, \ldots, t_i + \tau\gamma\}$ with $\gamma = 3600$:

$$\mathrm{Nadir}(t_i) = f\left(\underset{t \in I_i}{\arg\max}|f(t)|\right),$$

$$\mathrm{Integral}(t_i) = \tau \sum_{t \in I_i} f(t),$$

$$\mathrm{MSD}(t_i) = \frac{1}{\gamma} \sum_{t \in I_i} f^2(t).$$

From the derivative of the frequency time series $\frac{df}{dt}(t)$, we obtained the hourly (positive or negative) RoCoF by looking for the steepest slope within a window $W_i = [t_i - T, t_i + T]$ of length $2T$ around the beginning of the hour t_i:

$$\mathrm{RoCoF}(t_i) = \frac{df}{dt}\left(\underset{t \in W_i}{\arg\max}\left|\frac{df}{dt}\right|\right).$$

We estimated the derivative $\frac{df}{dt}(t)$ using a low-pass filter on the frequency increments,[60] i.e., by smoothing the increments $\Delta f(t) = f(t) - f(t - \tau)$ with a rectangular rolling window of length L. We chose the parameters L and T in such a way that they accounted for the different timescales of the RoCoF in the synchronous areas (supplemental experimental procedures S2). This resulted in a choice of $L = T = 60$s for the CE and GB areas, while the Nordic area with its fast hydropower exhibited larger RoCoFs so that we chose $L = T = 30$s instead.

Data preparation of external features

We collected different power system time series as external features to predict frequency deviations in Europe. We retrieved six different sets of publicly available time series from the ENTSO-E transparency platform.[36] These sets comprise the day-ahead load forecast, day-ahead scheduled generation, day-ahead wind and solar power forecast, day-ahead electricity prices, actual load, and actual generation per production type. Most of the time series are available on an hourly basis. Since we predicted stability indicators on an hourly basis, we downsampled a few higher-resolution time series to a common hourly resolution.

We then aggregated the data within the three synchronous areas. Since time series from ENTSO-E are only available for smaller regions within the synchronous areas (i.e., countries), we added up the load and generation data within each area. To aggregate the price data, we calculated area-wide averages weighted by the regional mean load. The time series from the ENTSO-E transparency platform contained multiple missing or corrupted data points,[14] which required a careful aggregation and cleansing procedure (supplemental experimental procedures S1). We deemed area-wide feature aggregation necessary because all locations within the synchronous power grid contribute to large frequency deviations.[1] We additionally prepared selected country-level data for the CE and the Nordic areas. The area-wide aggregated features resulted in a similar or higher model performance than country-level data (supplemental experimental procedures S5). Therefore, we decided to use area-wide aggregated features for this publication. An overview of the available (aggregated) features per area is available in supplemental experimental procedures S1.

Finally, we engineered additional meaningful features based on the hourly ENTSO-E time series $X(t_i)$, which comprise both day-ahead forecast data $X_{D-1}(t_i)$ and actual data $X_D(t_i)$. For each hourly interval $\Delta t = \tau\gamma$, we introduced ramp features (slopes) $(X(t_i) - X(t_i - \Delta t))/\Delta t$, which are inspired by the importance of generation ramps for the CE frequency dynamics.[4] We also added forecast errors $X_{D-1}(t_i) - X_D(t_i)$ and the artificial features of hours (of the day), weekdays, and months. To include the total available inertia as a feature, we calculated the sum of the synchronous generation which approximates to the time-dependent inertia.[38]

In summary, our dataset comprises hourly time series of 4 stability indicators (model outputs or targets) and 66 external features (model inputs) for the years 2015–2019. The dataset is available on Zenodo[30] and our scripts for downloading and preparing the dataset are online at GitHub.[50]

GTB model

To predict indicators of frequency stability from external features, we used GTB. Tree-based ensemble methods, such as GTB, are complex, nonlinear ML models, which makes them suitable for predicting the nonlinear behavior of power grids.[1] They offer a quick method of calculating SHAP values, thus facilitating efficient post-modeling explanation.[24] In addition, they are immune to the effects of feature outliers and perform inherent feature selection, making them robust to the inclusion of correlated or irrelevant features.[16] This is beneficial for our dataset, which exhibits strongly correlated features (supplemental experimental procedures S3) as well as outliers due to bad data quality (supplemental experimental procedures S1).

To fit our GTB model, we used XGBoost, which is a scalable gradient tree boosting system that provides state-of-the-art results for many ML applications.[43] We randomly split our data into a training set (64%), a validation set (16%), and a test set (20%). To optimize the hyperparameters of the XGBoost model, we performed a grid search over selected parameter values and evaluated the performance via 5-fold cross-validation on our training set. To determine the number of trees in the XGBoost models, we performed early stopping on the validation set. Finally, we concatenated the training and validation sets, retrained the model on this data with optimal hyperparameters, and tested its performance on the unseen test set. We also calculated the SHAP values on the test set. The detailed implementation in Python code is available on GitHub[50] and the sets of final hyperparameters are online at Zenodo.[30]

To quantify the model performance, we evaluated the R^2 score, which quantifies the proportion of variability explained by the model. Predicting the true targets results in a score of 1, while always predicting the mean of the target gives a score of 0. To benchmark our predictor, we compared its performance with the daily profile prediction. The daily profile, i.e., the daily average evolution of a target, is the most important recurring pattern of frequency dynamics.[61] Predicting the stability indicators based on their daily profiles thus represents an important null model. Our GTB model consistently outperformed the daily profile for all areas and indicators (see supplemental experimental procedures S5 for a detailed performance evaluation).

Model interpretation with SHAP

SHAP values can explain the output of any ML model.[23] Based on the game-theoretical Shapley values, they attribute a model output to the individual effects of each input feature. In particular, SHAP values quantify the marginal effect of including a feature into the prediction and comparing them with a randomized baseline.[24,62] Within the class of additive feature attributions, they guarantee certain optimal properties, such as local accuracy and consistency.[22] As they are locally accurate, the SHAP values always add up to the total model output. Consistency guarantees that a SHAP value does not decrease if the corresponding feature contributes more to the prediction when the model is altered.

SHAP values represent the feature effects on individual model outputs relative to the base value, which is given by the average prediction (cf. Figure 1). By combining many of these local explanations, SHAP values also offer global insights.[24]

The mean absolute SHAP value measures the global importance of a feature within a model. We identified the five most important features for each stability indicator and area (Figure 2). Figure 2 also displays feature importances for the *union* of these feature sets, i.e. features with an importance rank below five are also displayed. In addition to global feature importances, dependency plots show how the effect of a feature changes with the value of the feature (e.g., Figure 4D). Notably, these dependencies differ from observing relationships in scatterplots or between targets and features in a simple correlation analysis. Such model-agnostic methods cannot distinguish the effect of two correlated features. In contrast, we estimated interventional SHAP values, which quantify the marginal feature effect in the model by breaking down correlations with other features.[63,64]

In addition to first-order attributions, SHAP offers interaction values that attribute the model output to pairs of interacting features.[24] Interaction values decompose the first-order SHAP effects into diagonal effects and pairwise interaction effects (such as in Figure 5). The interaction effects therefore explain the vertical dispersion within the first-order SHAP dependency plots, thus offering scientific insights as well as additional consistency checks for the model applications.

Finally, there is a fundamental difference between predictive models and causal models.[65] Predictive models try to infer the conditional probability of the target given the feature variables by fitting associations. Causal models identify the effect on the target when manipulating or intervening on a feature. ML models, such as the boosted trees used here, are typically predictive models. Using XAI methods to explain how these ML models work reveals only associations learned from the data.[21] In particular, using SHAP values to explain predictive models does not necessarily reflect causal effects.[66] However, causation involves correlation so that predictive and explainable ML models can suggest causal dependencies, which then have to be further validated, e.g. by domain knowledge or causal inference methods.

Aggregated SHAP values

To explain daily average profiles of the model predictions, we visualized the SHAP values in a way that builds on their additivity. Due to their property of "local accuracy," the prediction $f(t)$ at every point in time t can be written as a sum of the respective SHAP values,

$$f(t) = \varphi_0 + \sum_{j=1}^{N} \varphi_j(t), \qquad \text{(Equation 1)}$$

where $\varphi_j(t)$ is the SHAP value of feature j at time t. This property of SHAP values enables a new application in the analysis of daily profiles and other recurrent patterns. The daily profile of the prediction is the average $\langle f(t) \rangle_h$ for the hour h over all days. Based on the SHAP values $\varphi_j(t)$ for feature j ($j = 1, \ldots, N$) and their base value φ_0,[24] we decomposed the daily profile as follows:

$$\langle f(t) \rangle_h = \langle \varphi_0 + \sum_{j=1}^{N} \varphi_j(t_i) \rangle_h = \varphi_0 + \sum_{j=1}^{N} \langle \varphi_j(t) \rangle_h.$$

The daily aggregated SHAP values $\langle \varphi_j \rangle_h$ then explain the daily profile of the prediction. To display the daily SHAP values, such as in Figures 4A–4C, we identified the three most important features according to their average effect $\frac{1}{24}\sum_{h=1}^{24} |\langle \varphi_j \rangle_h|$ on the daily profile in each area. In Figures 4A–4C, we then visualized these features from the union of these sets to display the most important daily SHAP values. The remaining daily SHAP values were aggregated and displayed as a residual variable.

Finally, we add three notes on the interpretation of (daily) aggregated SHAP values. (1) We note that the aggregated SHAP values do *not* coincide with SHAP values of a model trained on the aggregated data. This must be taken into account when interpreting the results. (2) Due to the nonlinearity of an ML model, a large daily SHAP value does not necessarily correspond to a large average for the corresponding feature in that hour. (3) Second-order interactions between features are "fairly" distributed between first-order SHAP values according to the classical Shapley values,[24] i.e., large daily SHAP values can partly relate to strong interaction within this specific hour. To further resolve interactions within the daily SHAP values, the additivity of second-order SHAP values can be used to generate daily profiles of the interactions. This is beyond the scope of this paper.

Relative ramping rates

We used relative ramping rates to validate our SHAP results, particularly for the prediction of the RoCoF. In particular, we quantified the relative ramping speed of each conventional generation technology k within a synchronous area. The ramping speed $\tilde{s}_k$ is determined both by the absolute change of generation ΔX_k and the timescale λ_k on which the generator adapts its output to the new set point:

$$\tilde{s}_k := \frac{\Delta X_k}{\lambda_k}.$$

We approximated the typical value of ΔX_k with the median of the absolute generation changes $\Delta X_k \approx \mathrm{Median}_{t_i} |X_k(t_i - \Delta t) - X_k(t_i)|$. The *relative* ramp speed s_k, compared with the fastest technology m within the area, then reads

$$s_k = \frac{\tilde{s}_k}{\tilde{s}_m} = \frac{\Delta X_k}{\Delta X_m} \frac{\lambda_m}{\lambda_k} \approx \frac{\Delta X_k}{\Delta X_m} \frac{r_k}{r_m}.$$

Finally, we approximated the ratio of timescales λ_k by using the inverse ratio of technology-specific ramping rates r_k.[67] The technology m with the largest absolute ramping speed was determined by the maximum value of $\Delta X_k r_k$.

SUPPLEMENTAL INFORMATION

Supplemental information can be found online at https://doi.org/10.1016/j.patter.2021.100365.

ACKNOWLEDGMENTS

We would like to thank Bo Tranberg for fruitful discussions. Furthermore, we gratefully acknowledge support from the German Federal Ministry of

CellPress
OPEN ACCESS

Education and Research (BMBF grant no. 03EK3055B) and the Helmholtz Association via the Helmholtz School for Data Science in Life, Earth and Energy (HDS-LEE). This project has received funding from the European Union's Horizon 2020 research and innovation program under the Marie Skłodowska-Curie grant agreement no. 840825.

AUTHOR CONTRIBUTIONS

J.K., B.S., and D.W. conceived and designed the research. J.K. trained the model and produced the figures. All authors contributed to discussing and interpreting the results and writing the manuscript. B.S. and D.W. contributed equally.

DECLARATION OF INTERESTS

The authors declare no competing interests.

Received: May 31, 2021
Revised: July 23, 2021
Accepted: September 16, 2021
Published: October 8, 2021

REFERENCES

1. Machowski, J., Bialek, J., Bumby, J., and Bumby, D.J. (2008). Power System Dynamics: Stability and Control (John Wiley & Sons).
2. Pourbeik, P., Kundur, P.S., and Taylor, C.W. (2006). The anatomy of a power grid blackout—root causes and dynamics of recent major blackouts. IEEE Power Energy Mag. *4*, 22–29.
3. Milano, F., Dörfler, F., Hug, G., Hill, D.J., and Verbič, G. (2018). Foundations and challenges of low-inertia systems (invited paper). In 2018 Power Systems Computation Conference (PSCC) (IEEE), pp. 1–25.
4. Weissbach, T., and Welfonder, E. (2009). High frequency deviations within the European Power System: origins and proposals for improvement. In 2009 IEEE/PES Power Systems Conference and Exposition (IEEE), pp. 1–6.
5. Vorobev, P., Greenwood, D.M., Bell, J.H., Bialek, J.W., Taylor, P.C., and Turitsyn, K. (2019). Deadbands, droop, and inertia impact on power system frequency distribution. IEEE Trans. Power Syst. *34*, 3098–3108.
6. Ayodele, T.R., Jimoh, A., Munda, J.L., and Tehile, A.J. (2012). Challenges of grid integration of wind power on power system grid integrity: a review. Int. J. Renew. Energy Res. *2*, 618–626.
7. Haehne, H., Schottler, J., Waechter, M., Peinke, J., and Kamps, O. (2018). The footprint of atmospheric turbulence in power grid frequency measurements. EPL (Europhys. Lett.) *121*, 30001.
8. Chen, L., Markham, P., Chen, C.-f., and Liu, Y. (2011). Analysis of societal event impacts on the power system frequency using FNET measurements. In 2011 IEEE Power and Energy Society General Meeting (IEEE), pp. 1–8.
9. Adeen, M., Jónsdóttir, G.M., and Milano, F. (2019). Statistical correlation between wind penetration and grid frequency variations in the Irish network. In 2019 IEEE International Conference on Environment and Electrical Engineering and 2019 IEEE Industrial and Commercial Power Systems Europe (EEEIC/I&CPS Europe), pp. 1–6.
10. Homan, S., Mac Dowell, N., and Brown, S. (2021). Grid frequency volatility in future low inertia scenarios: challenges and mitigation options. Appl. Energy *290*, 116723.
11. Persson, M., and Chen, P. (2017). Frequency evaluation of the Nordic power system using PMU measurements. IET Generation, Transmission and Distribution *11*, 2879–2887.
12. Gorjão, L.R., Schäfer, B., and Hassan, G. (2020). Open Access Power-Grid Frequency Database (OSF). https://doi.org/10.17605/OSF.IO/M43TG.
13. Kruse, J., Schäfer, B., and Witthaut, D. (2020a). Pre-Processed Power Grid Frequency Time Series (Zenodo). https://doi.org/10.5281/zenodo.3744121.
14. Hirth, L., Mühlenpfordt, J., and Bulkeley, M. (2018). The ENTSO-E Transparency Platform—a review of Europe's most ambitious electricity data platform. Appl. Energy *225*, 1054–1067.
15. Morrison, R. (2018). Energy system modeling: public transparency, scientific reproducibility, and open development. Energy Strategy Rev. *20*, 49–63.
16. Hastie, T., Tibshirani, R., and Friedman, J. (2016). The Elements of Statistical Learning: Data Mining, Inference, and Prediction, 2nd edition (Springer).
17. Adadi, A., and Berrada, M. (2018). Peeking inside the black-box: a survey on explainable artificial intelligence (XAI). IEEE Access *6*, 52138–52160.
18. Roscher, R., Bohn, B., Duarte, M.F., and Garcke, J. (2020). Explainable machine learning for scientific insights and discoveries. IEEE Access *8*, 42200–42216.
19. Ahmad, T., Zhang, D., Huang, C., Zhang, H., Dai, N., Song, Y., and Chen, H. (2021). Artificial intelligence in sustainable energy industry: status quo, challenges and opportunities. J. Clean. Prod. *289*, 125834.
20. Cremer, J.L., Konstantelos, I., and Strbac, G. (2019). From optimization-based machine learning to interpretable security rules for operation. IEEE Trans. Power Syst. *34*, 3826–3836.
21. Barredo Arrieta, A., Díaz-Rodríguez, N., Del Ser, J., Bennetot, A., Tabik, S., Barbado, A., Garcia, S., Gil-Lopez, S., Molina, D., Benjamins, R., et al. (2020). Explainable artificial intelligence (XAI): concepts, taxonomies, opportunities and challenges toward responsible AI. Inf. Fusion *58*, 82–115.
22. Lungberg, S.M., Erion, G.G., and Lee, S.-I. (2019). Consistent individualized feature attribution for tree ensembles. arXiv, arXiv:1802.03888 https://arxiv.org/abs/1802.03888.
23. Lundberg, S.M., and Lee, S.-I. (2017). A unified approach to interpreting model predictions. In Proceedings of the 31st International Conference on Neural Information Processing Systems, NIPS'17 (Curran Associates Inc.), pp. 4768–4777.
24. Lundberg, S.M., Erion, G., Chen, H., DeGrave, A., Prutkin, J.M., Nair, B., Katz, R., Himmelfarb, J., Bansal, N., and Lee, S.-I. (2020). From local explanations to global understanding with explainable AI for trees. Nat. Machine Intelligence *2*, 56–67.
25. Lundberg, S.M., Nair, B., Vavilala, M.S., Horibe, M., Eisses, M.J., Adams, T., Liston, D.E., Low, D.K.-W., Newman, S.-F., Kim, J., and Lee, S.-I. (2018). Explainable machine-learning predictions for the prevention of hypoxaemia during surgery. Nat. Biomed. Eng. *2*, 749–760.
26. Padarian, J., McBratney, A.B., and Minasny, B. (2020). Game theory interpretation of digital soil mapping convolutional neural networks. Soil *6*, 389–397.
27. Kuzlu, M., Cali, U., Sharma, V., and Güler, Ö. (2020). Gaining insight into solar photovoltaic power generation forecasting utilizing explainable artificial intelligence tools. IEEE Access *8*, 187814–187823.
28. Chen, M., Liu, Q., Chen, S., Liu, Y., Zhang, C., and Liu, R. (2019). XGBoost-based algorithm interpretation and application on post-fault transient stability status prediction of power system. IEEE Access *7*, 13149–13158.
29. Alova, G., Trotter, P.A., and Money, A. (2021). A machine-learning approach to predicting Africa's electricity mix based on planned power plants and their chances of success. Nat. Energy *6*, 158–166.
30. Kruse, J., Schäfer, B., and Witthaut, D. (2021c). Supplementary Data: "Revealing Drivers and Risks for Power Grid Frequency Stability with Explainable AI" (Zenodo). https://doi.org/10.5281/zenodo.5118352.
31. Meyer, P.G., Anvari, M., and Kantz, H. (2020). Identifying characteristic time scales in power grid frequency fluctuations with DFA. Chaos *30*, 013130.
32. Gross, D., Bolognani, S., Poolla, B.K., and Dörfler, F. (2017). Increasing the resilience of low-inertia power systems by virtual inertia and damping. In Proceedings of IREP'2017 Symposium (IREP), p. 64.
33. Tyloo, M., and Jacquod, P. (2021). Primary control effort under fluctuating power generation in realistic high-voltage power networks. IEEE Control. Syst. Lett. *5*, 929–934.

34. Gorjão, L.R., Vanfretti, L., Witthaut, D., Beck, C. and Schäfer, B. (2021). Phase and amplitude synchronisation in power-grid frequency fluctuations in the Nordic grid. arXiv, arXiv:2105.00228, https://arxiv.org/abs/2105.00228.
35. Gorjão, L.R., Jumar, R., Maass, H., Hagenmeyer, V., Yalcin, G.C., Kruse, J., et al. (2020). Open database analysis of scaling and spatio-temporal properties of power grid frequencies. Nat. Commun. *11*, 6362.
36. ENTSO-E. (2020). Transparency Platform. https://transparency.entsoe.eu/.
37. Lin, J., and Magnago, F.H. (2017). Electricity Markets: Theories and Applications (John Wiley & Sons).
38. Ulbig, A., Borsche, T.S., and Andersson, G. (2014). Impact of low rotational inertia on power system stability and operation. IFAC Proc. Vol. *47*, 7290–7297.
39. Schmietendorf, K., Peinke, J., and Kamps, O. (2017). The impact of turbulent renewable energy production on power grid stability and quality. Eur. Phys. J. B *90*, 222.
40. Gorjão, L.R., Anvari, M., Kantz, H., Beck, C., Witthaut, D., Timme, M., and Schäfer, B. (2020). Data-driven model of the power-grid frequency dynamics. IEEE Access *8*, 43082–43097.
41. ENTSO-E. (2019). Report on Deterministic Frequency Deviations. https://consultations.entsoe.eu/system-development/deterministic_frequency_deviations_report/user_uploads/report_deterministic_frequency_deviations_final-draft-for-consultation.pdf.
42. Weißbach, T., Remppis, S., and Lens, H. (2018). Impact of current market developments in Europe on deterministic grid frequency deviations and frequency restauration reserve demand. In 2018 15th International Conference on the European Energy Market (EEM) (IEEE), pp. 1–6.
43. Chen, T., and Guestrin, C. (2016). XGBoost: a scalable tree boosting system. In Proceedings of the 22nd ACM SIGKDD International Conference on Knowledge Discovery and Data Mining, KDD '16 (ACM), pp. 785–794.
44. Svenska kraftnät, Statnett, Energinet.dk, and Fingrid. (2016). Challenges and Opportunities for the Nordic Power System. https://www.fingrid.fi/globalassets/dokumentit/fi/yhtio/tki-toiminta/report-challenges-and-opportunities-for-the-nordic-power-system.pdf.
45. Nationalgrid ESO. (2019b). Operability Strategy Report 2019. https://www.nationalgrideso.com/document/159726/download.
46. Schäfer, B., Witthaut, D., Timme, M., and Latora, V. (2018). Dynamically induced cascading failures in power grids. Nat. Commun. *9*, 1–13.
47. Nationalgrid ESO. (2019a). Monthly Balancing Services Summary 2019/20. https://www.nationalgrideso.com/document/119361/download.
48. Díaz-González, F., Hau, M., Sumper, A., and Gomis-Bellmunt, O. (2014). Participation of wind power plants in system frequency control: review of grid code requirements and control methods. Renew. Sustain. Energy Rev. *34*, 551–564.
49. ENTSO-E Balancing Report 2020 (2020). https://eepublicdownloads.azureedge.net/clean-documents/Publications/Market%20Committee%20publications/ENTSO-E_Balancing_Report_2020.pdf
50. Kruse, J., Schäfer, B., and Witthaut, D. (2021a). Explainable Machine Learning for Power Grid Frequency Stability. https://doi.org/10.5281/zenodo.5497609.
51. Kruse, J., Schäfer, B., and Witthaut, D. (2021b). Exploring deterministic frequency deviations with explainable AI. arXiv, arXiv:2106.09538 https://arxiv.org/abs/2106.09538.
52. Avramiotis-Falireas, I., Troupakis, A., Abbaspourtorbati, F., and Zima, M. (2013). An MPC Strategy for Automatic Generation Control with Consideration of Deterministic Power Imbalances. In 2013 IREP Symposium Bulk Power System Dynamics and Control - IX Optimization, Security and Control of the Emerging Power Grid (IEEE), pp. 1–8.
53. Huang, L., Coulson, J., Lygeros, J., and Dörfler, F. (2021). Decentralized data-enabled predictive control for power system oscillation damping. IEEE Trans. Control. Syst. Technol. https://doi.org/10.1109/TCST.2021.3088638.
54. Pfenninger, S. (2017). Energy scientists must show their workings. Nature *542*, 393.
55. Lana, I., Del Ser, J., Velez, M., and Vlahogianni, E.I. (2018). Road traffic forecasting: recent advances and new challenges. IEEE Intell. Transport. Syst. Mag. *10*, 93–109.
56. Schultz, M., Betancourt, C., Gong, B., Kleinert, F., Langguth, M., Leufen, L., Mozaffari, A., and Stadtler, S. (2021). Can deep learning beat numerical weather prediction? Philos. Trans. R. Soc. A *379*, 20200097.
57. Fingrid Oyj (2020). Frequency—Historical Data. https://data.fingrid.fi/en/dataset/frequency-historical-data.
58. Nationalgrid ESO. (2020). Historic Frequency Data. https://www.nationalgrideso.com/balancing-services/frequency-response-services/historic-frequency-data.
59. TransnetBW GmbH (2020). Regelenergie Bedarf + Abruf. https://www.transnetbw.de/de/strommarkt/systemdienstleistungen/regelenergie-bedarf-und-abruf.
60. Frigo, G., Derviškadić, A., Zuo, Y., and Paolone, M. (2019). PMU-based ROCOF measurements: uncertainty limits and metrological significance in power system applications. IEEE Trans. Instrum. Meas. *68*, 3810–3822.
61. Kruse, J., Schäfer, B., and Witthaut, D. (2020b). Predictability of power grid frequency. IEEE Access *8*, 149435–149446.
62. Sundararajan, M., and Najmi, A. (2020). The Many Shapley Values for Model Explanation (International Conference on Machine Learning, PMLR), pp. 9269–9278.
63. Chen, H., Janizek, J.D., Lundberg, S., and Lee, S.-I. (2020). True to the model or true to the data? arXiv, arXiv:2006.16234 https://arxiv.org/abs/2006.16234.
64. Janzing, D., Minorics, L., and Bloebaum, P. (2020). Feature relevance quantification in explainable AI: a causal problem. In International Conference on Artificial Intelligence and Statistics (PMLR), pp. 2907–2916.
65. Spirtes, P., and Zhang, K. (2016). Causal discovery and inference: concepts and recent methodological advances. Appl. Inform. *3*, 3.
66. Ma, S., and Tourani, R. (2020). Predictive and causal implications of using Shapley value for model interpretation. In Proceedings of the 2020 KDD Workshop on Causal Discovery (PMLR), pp. 23–38.
67. Gonzalez-Salazar, M.A., Kirsten, T., and Prchlik, L. (2018). Review of the operational flexibility and emissions of gas- and coal-fired power plants in a future with growing renewables. Renew. Sustain. Energy Rev. *82*, 1497–1513.

Patterns, Volume *2*

Supplemental information

Revealing drivers and risks for power grid frequency stability with explainable AI

Johannes Kruse, Benjamin Schäfer, and Dirk Witthaut

SUPPLEMENTAL EXPERIMENTAL PROCEDURES S1: EXTERNAL FEATURE AGGREGATION AND DATA CLEANSING

To model frequency stability indicators, we collect publicly available times series of external features from the ENTSO-E Transparency platform.[1] For the synchronous areas investigated here, we aggregate the ENTSO-E time series, which are originally only available for smaller regions within the areas (e.g., countries). However, the time series contain many missing data points, so that we need a careful procedure to aggregate the region contributions within the synchronous areas.

Firstly, we specify region types for which we obtain the best data quality. In all but a few cases, we retrieve country level data. Only in Continental Europe, we retrieve bidding zone data for Italy (North, Center North, Center South, South and Sicilia) and control zone data for Germany (TenneT, TransnetBW, 50Hertz, Amprion), as data quality is better for these regions. For Denmark, we also retrieve bidding zone data, as one zone belongs to the Continental Europe area, while the other belongs to the Nordic area.

Secondly, in the Continental Europe and the Nordic areas, we aggregate the region contributions and propagate missing data points through the data set. For each feature and region, we mark missing time steps as "NaN". Then, during aggregation, we propagate missing values by setting the sum of region contributions to NaN if at least one of them is NaN. For example, if the German load data had a missing value at 10:00 on the 6th of June 2018, the Continental European aggregated load data would have a NaN at this time step. Finally, we clean all aggregated features together with the grid frequency data. In particular, we omit a time step from the *whole* data set if at least one feature is NaN or the corresponding frequency measurements contains missing or corrupted values. This cleansed data set is used in model training and testing.

Notably, we will not have many data points left in the aggregated data set, if the region contributions contain too many NaNs. To avoid this problem, we collect the time series of each feature and region, which we call the *region-variable contribution*, and sort them according to their NaN share. Then, we successively add up the region-variable contributions with increasing NaN share. A contribution is only added if the NaN share S of the aggregated data set *including* the new contribution does not exceed a certain threshold S_T. If the data exceeds the threshold ($S > S_T$), we omit the contribution from the aggregation. We choose $S_T = 37\%$, which we found to result in a good balance between having enough data and including as many contributions as possible. As an example, consider a (hypothetical) area consisting of 2 countries, "A" and "B", with two features "Load" and "Total generation". Initially, we add load data from country A as this (hypothetically) has the lowest NaN share of 30%. Then, we add the total generation from country B with a NaN share of 31%. The contribution is not omitted, since the time steps with NaN values overlap and the final NaN share of the whole data set only yields 33% thus staying below the threshold. However, the last two region-variable contributions might not be added, as they introduce to many new NaN values.

This procedure omits a certain amount of data but allows us to retain a large sample size. In Continental Europe, most of the omitted contributions would increase the feature value by less than 30% on average (Figure S1A) and all omitted feature contributions are smaller than 5% of the total mean load (Figure S1B). In the Nordic area, we only omit the Finish day-ahead solar power forecast, accounting for 0.03% of the area total mean load, and in GB there are no omissions. The aggregated data sets obtained from this procedure contain more than 26800 data points in each area (Table S2). We thus generate large data sets to efficiently learn structures in the data, while still representing most of the load and generation within the areas.

Finally, we obtain 25 different (aggregated) times series of external features. Combining them with additional engineered features, such as forecast errors, we end up with 66 different external features (Table S1), which both contain day-ahead available features (such as the load forecast) and ex-post available features (such as the actual generation per type). None of the synchronous areas exhibits all 66 features and the number of model inputs thus varies between 50 and 64 (Table S2).

SUPPLEMENTAL EXPERIMENTAL PROCEDURES S2: ROCOF EXTRACTION

The Rate of Change of Frequency (RoCoF) is an indicator of frequency stability, which we use in our study. We extract the RoCoF at the beginning of each hour by smoothing the frequency increments with a rolling window of length L and then looking for the maximum (absolute) RoCoF within a window of $\pm T$ around the full hour.

We choose the values of L and T according to the typical time scale of the RoCoF in the three different synchronous areas. The average hourly evolution of the absolute frequency deviation indicates this time scale (Figure S4). In Continental Europe and Great Britain, the average deviation reaches its maximum 60 s after the full hour, while the Nordic area exhibits its peak already after 30 s. We thus choose $L = T = 60$ s in the Continental Europe and Great Britain areas, but a shorter time scale of $L = T = 30$ s in the Nordic grid area.

Ex-post	Ramps [MW/h]	Load ramp, Total generation ramp, Biomass ramp, Coal gas ramp, Fossil peat ramp, Gas ramp, Geothermal ramp, Hard coal ramp, Lignite ramp, Nuclear ramp, Offshore wind ramp, Onshore wind ramp, Oil ramp, Other ramp, Other renewables ramp, Pumped hydro ramp, Reservoir hydro ramp, Run-off-river hydro ramp, Solar ramp, Waste ramp
	Generation and load [MW]	Load, Total generation, Synchronous generation, Biomass generation, Coal gas generation, Fossil peat generation, Gas generation, Geothermal generation, Hard coal generation, Lignite generation, Nuclear generation, Oil generation, Other generation, Other renewable generation, Pumped hydro generation, Reservoir hydro generation, Run-off-river hydro generation, Solar generation, Waste generation, Wind offshore generation, Wind onshore generation,
	Forecast errors of generation and load [MW]	Forecast error load, Forecast error total generation, Forecast error solar, Forecast error offshore wind, Forecast error onshore wind
	Forecast errors of ramps [MW/h]	Forecast error load ramp, Forecast error generation ramp, Forecast error solar ramp, Forecast error offshore wind ramp, Forecast error onshore wind ramp
Day-ahead	Generation and load [MW]	Load day-ahead, Scheduled generation, Solar day-ahead, Offshore wind day-ahead, Onshore wind day-ahead
	Ramps [MW/h]	Load ramp day-ahead, Generation ramp day-ahead, Solar ramp day-ahead, Offshore wind ramp day-ahead, Onshore wind ramp day-ahead
	Other	Price ramp day-ahead [Currency/MWh/h], Prices day-ahead [Currency/MWh], Hour, Weekday, Month

Table S1. All external features in the data set. The units correspond to those used in our publicly available data set.[2]

Area	Number of features	Number of data points
Continental Europe	64	26857
Nordic	58	37154
Great Britain	50	43240

Table S2. Properties of our data sets.

SUPPLEMENTAL EXPERIMENTAL PROCEDURES S3: BASIC CORRELATION ANALYSIS

A basic correlation analysis between external features and frequency stability indicators can already reveal interesting dependencies. However, this model-agnostic correlation analysis does not account for correlations among the features, which might affect the correlation coefficient between a feature and the stability indicator. Following the main text, we demonstrate this for the effect of nuclear ramps on the RoCoF in Continental Europe.

As depicted in Figure S5, there are various strong correlations between features in all three grid areas. For example, nuclear power generation is positively correlated with the load in Continental Europe. In Figure S6, the features are correlated with our four stability indicators. We observe that nuclear ramps are positively correlated with the RoCoF in Continental Europe, which is not consistent with our SHAP results (see main text). We can explain the positive correlation of nuclear ramps with the hidden relationships to other variables, such as load ramps. Load ramps have a positive effect on the RoCoF in Continental Europe (Figure S10). Due to the strong correlation between load and nuclear power generation (Figure S5) the effect of load ramps can thus "leak" into the correlation coefficients of nuclear ramps. This can explain why we observe a positive correlation between nuclear ramps and the RoCoF in Continental Europe, although nuclear ramps are RoCoF-offsetting in this area, as revealed by SHAP analysis in the main text.

SUPPLEMENTAL EXPERIMENTAL PROCEDURES S4: DETERMINISTIC FREQUENCY DEVIATIONS

Deterministic frequency deviations (DFDs) occur at the beginning of electricity trading intervals.[4] The generation is adapted in a step-wise manner at the beginning of these intervals, which are mostly hourly time periods. The mismatch between the step-wise generation and the continuously evolving load generates an instantaneous power imbalance, which causes a deterministic frequency jump at the beginning of the hour.

Such DFDs are an important factor for frequency stability in Continental Europe. This is indicated by the time within the hour when the absolute frequency deviation peaks ("Nadir occurrence time"). Figure S12 shows the histograms of these Nadir occurrence times within the hour. In Continental Europe, most of the Nadirs occur in the

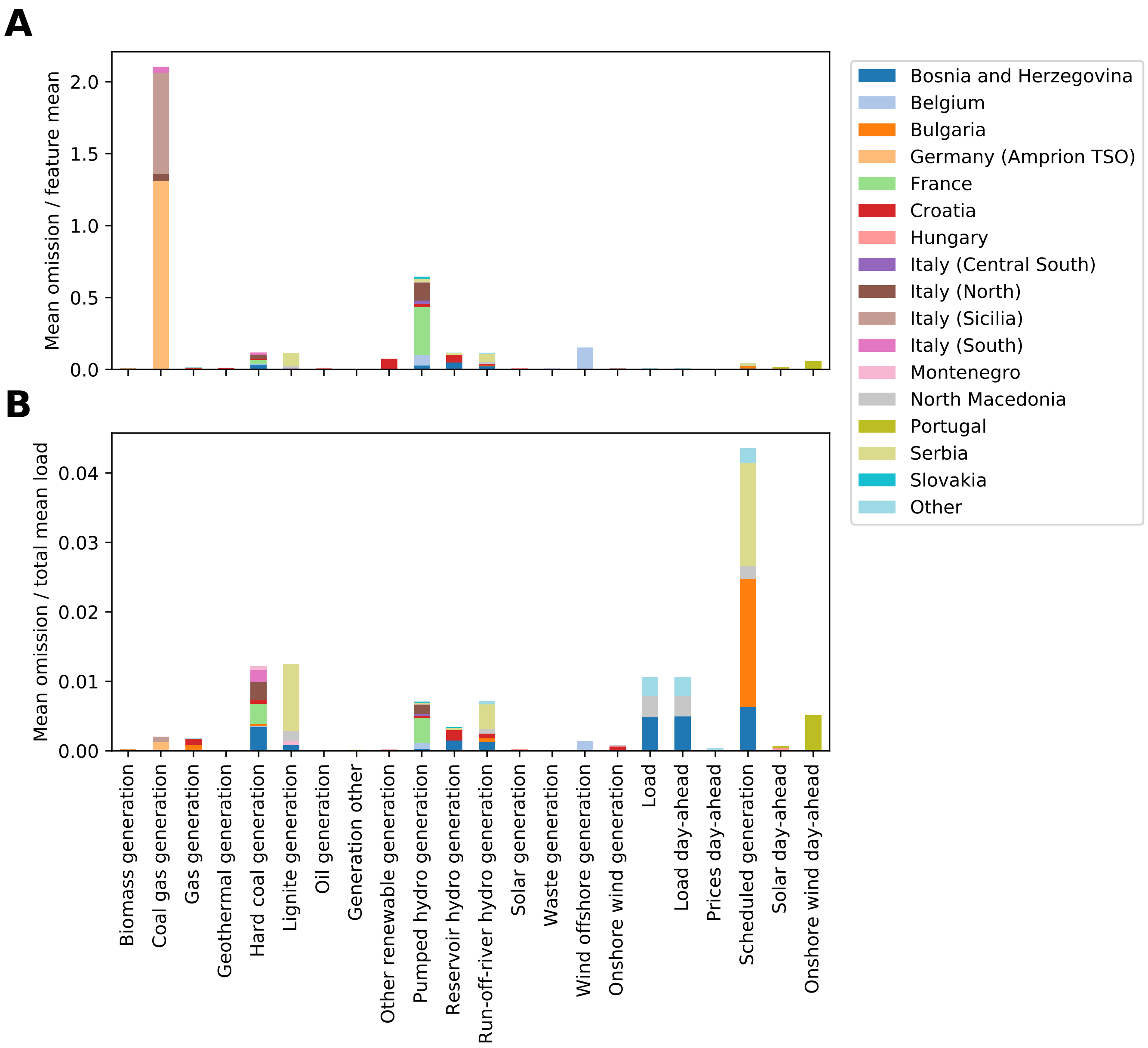

Figure S1. Omitted data contributions in Continental Europe. (a): We quantify the omitted values with the mean omitted feature value relative to the mean of the included features. The legend indicates the regions (mostly countries) where the omitted contributions come from. Regions with omitted (relative) contributions below 0.8% are aggregated in the "Other" variable. (b) The mean omitted feature value relative to the total mean load of the synchronous area remains below 5%.

first five minutes, which indicates their strong connection to the deterministic electricity trading. In contrast, large deviations in Great Britain occur much more often during the hour and not only at the beginning. This indicates that DFDs play a smaller role in Great Britain than in Continental Europe. The Nordic area is in between, showing strong hourly DFDs as well as Nadirs within the hour.

An extension of the work presented here could consider a threshold for the nadir and turn our regression machine learning task into a classification taks: Will the nadir of the next hour be above or below the security threshold?

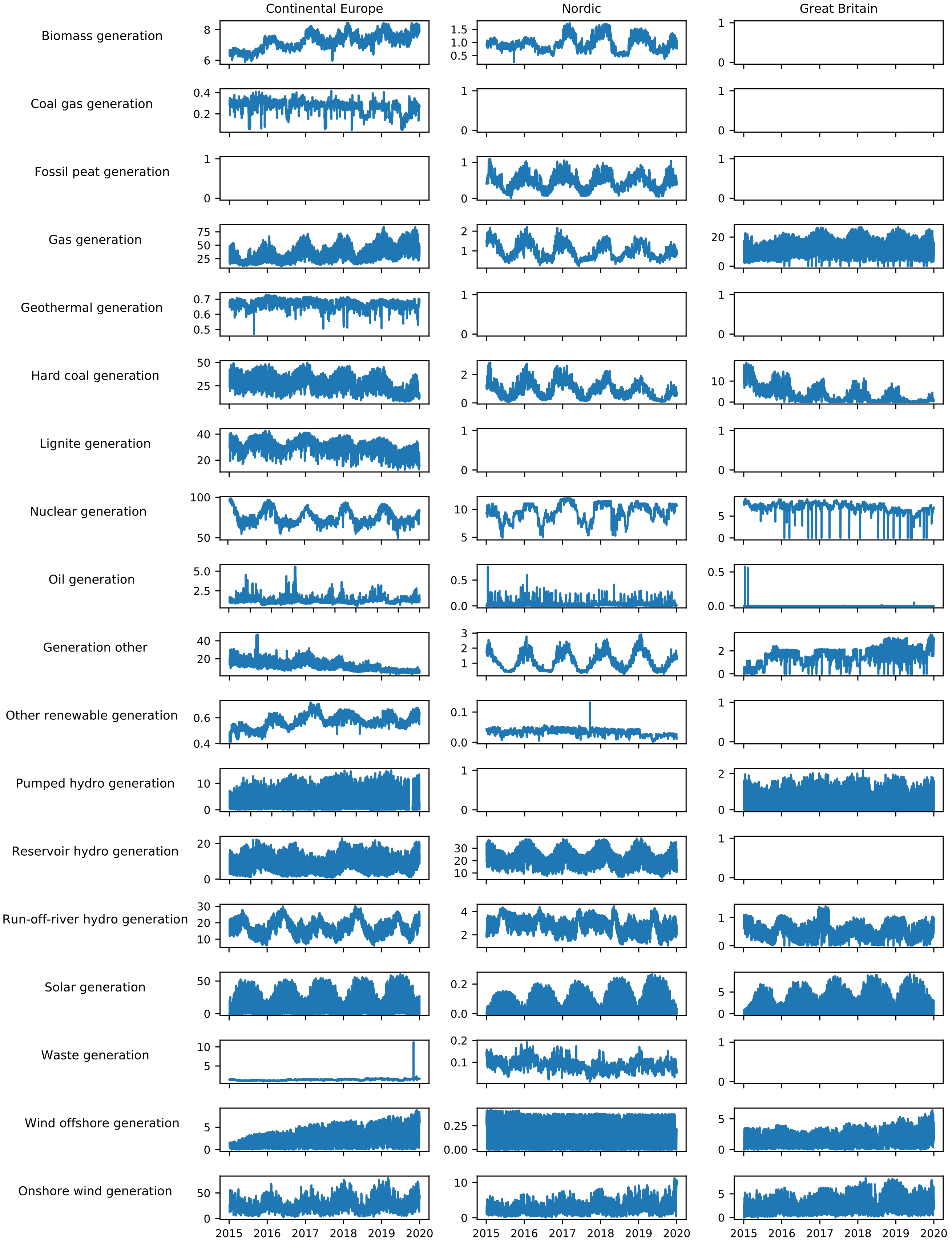

Figure S2. All (aggregated) time series from the ENTSO-E transparency platform:[1] Actual generation per type (in GW).

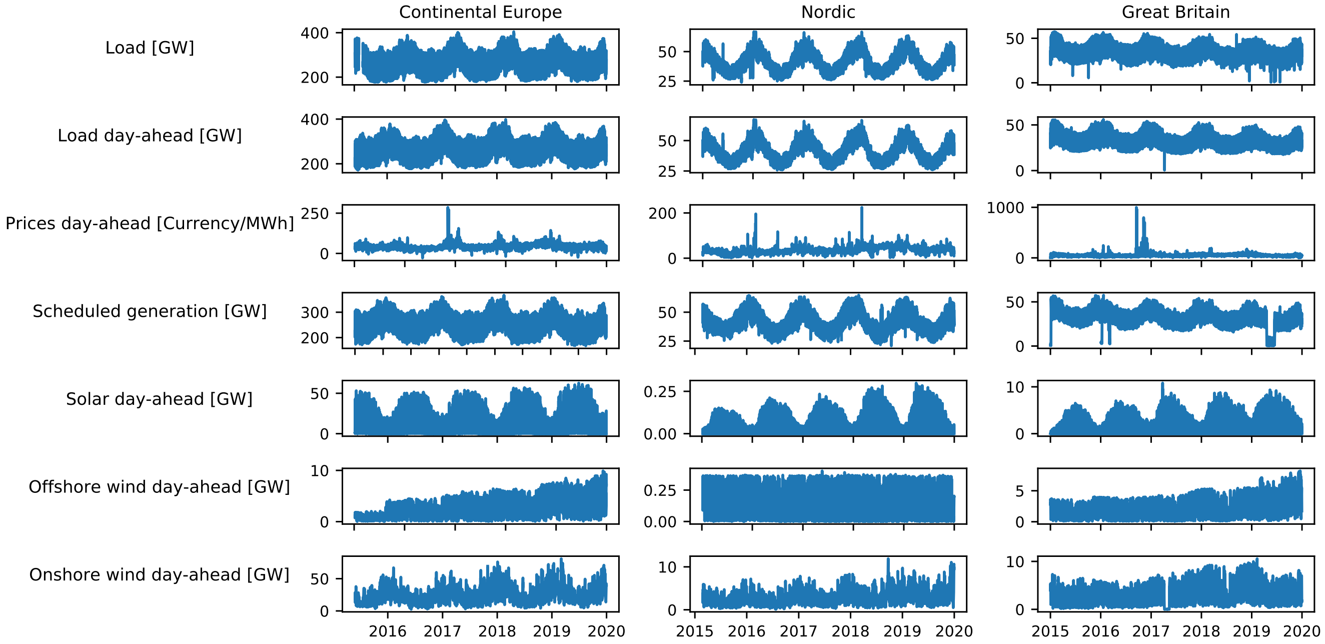

Figure S3. All (aggregated) time series from the ENTSO-E transparency platform:[1] All features except from actual generation per type.

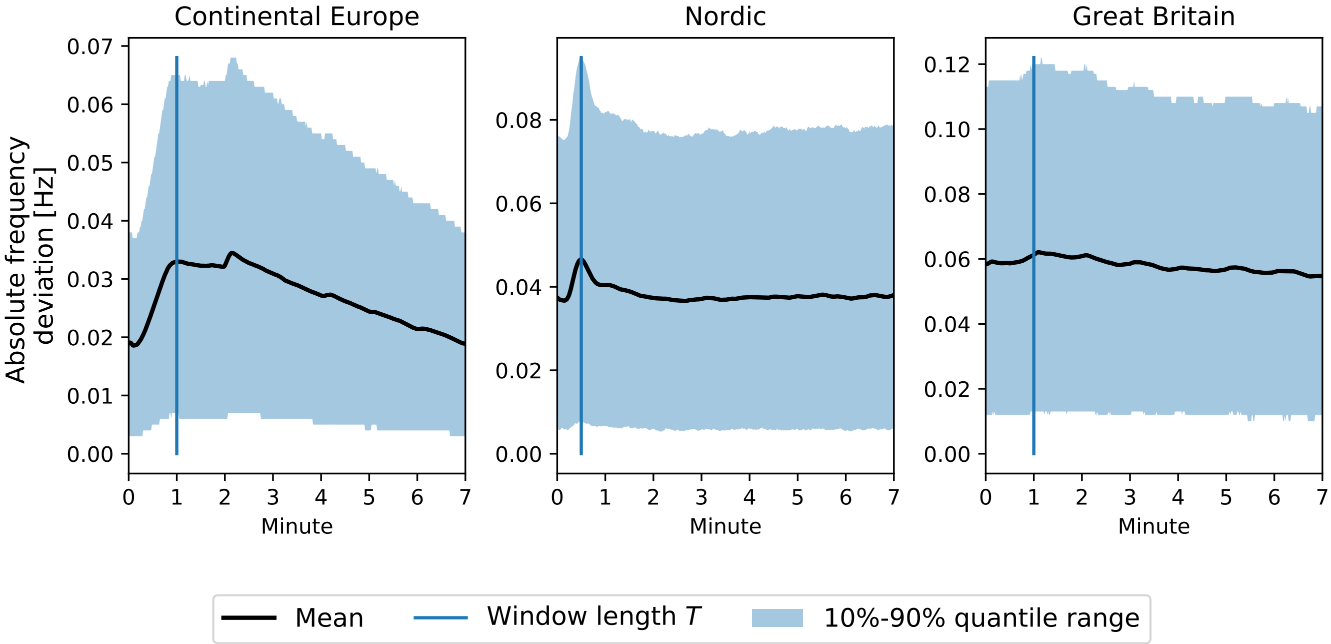

Figure S4. Evolution of hourly absolute frequency deviations. We display the mean evolution of the absolute frequency deviation during the first seven minutes of an hour. The deviation peaks at the beginning of the hour due to the impact of electricity trading.[4] The time scale of this initial increase depends on the grid area. We choose the window length T for the RoCoF extraction according to this time scale.

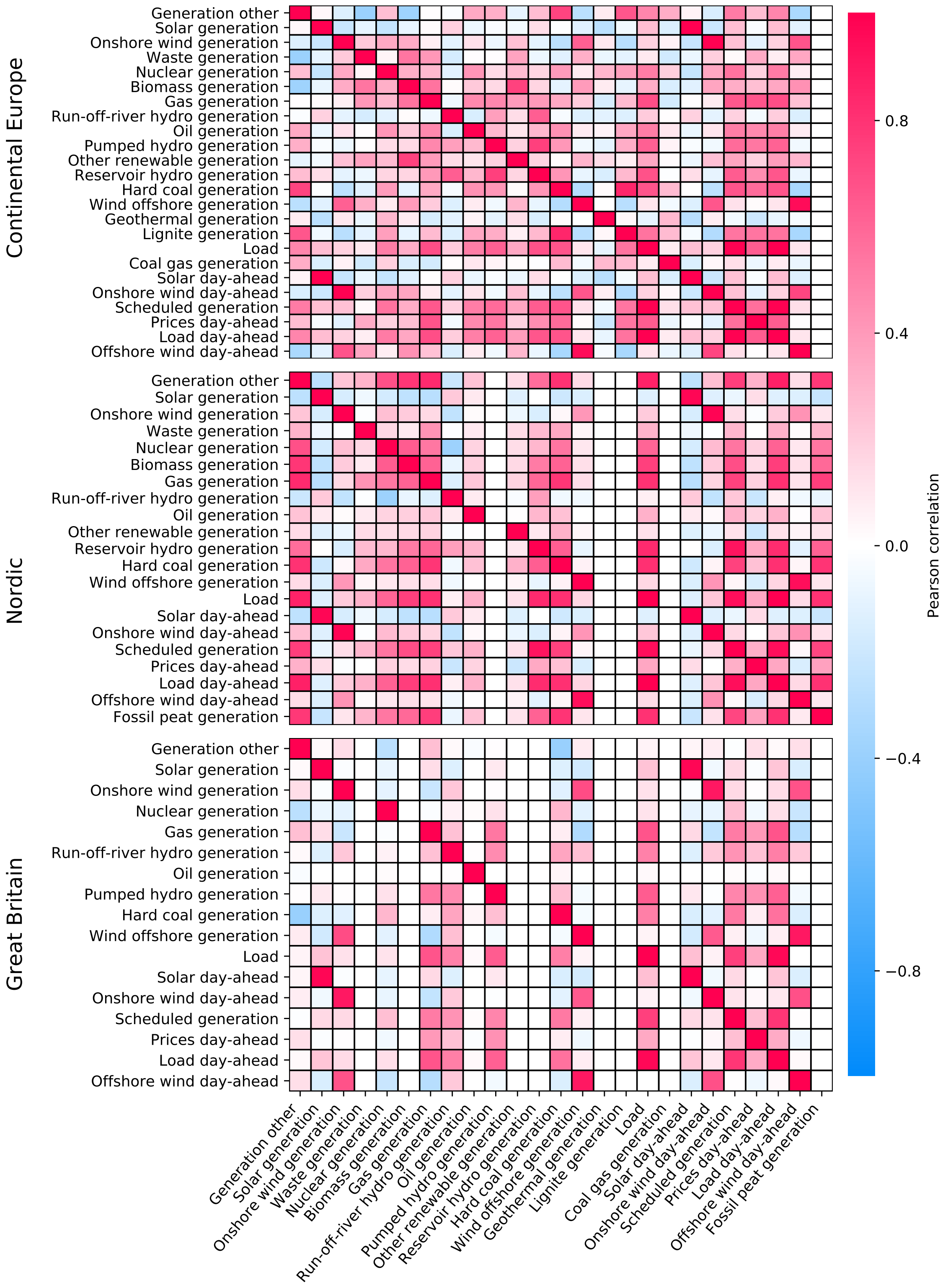

Figure S5. Pearson correlation coefficients between external features. To improve the visibility, we exclude our additional, engineered features from this plot. Features with 0 correlation everywhere have no data in a specific grid (e.g., Fossil peat data is only available in Nordic).

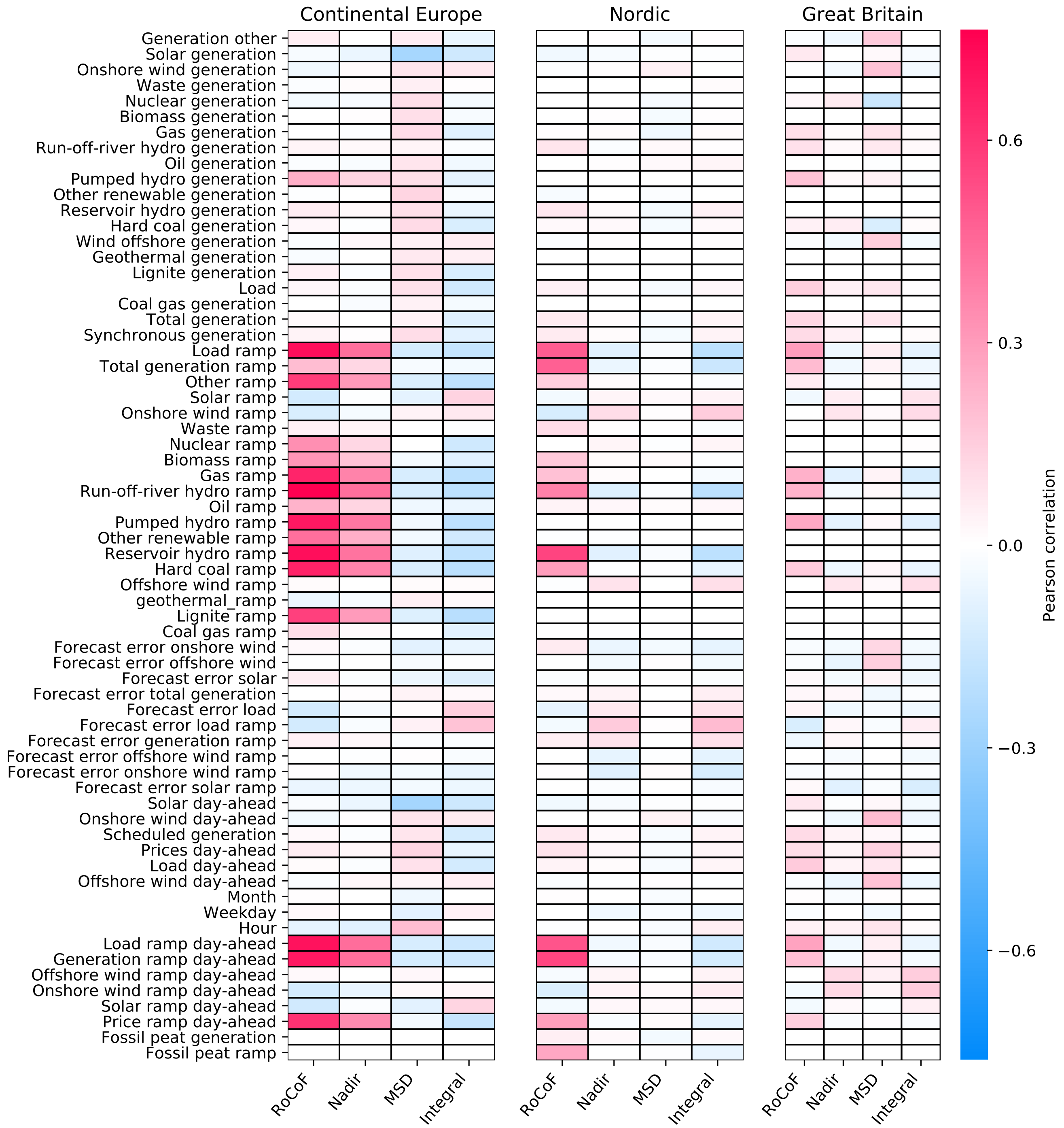

Figure S6. Pearson correlation coefficients between external features and frequency stability indicators. The scale of the colour code is adjusted to the maximum absolute correlation value C_{max} and thus ends at $\pm C_{max}$.

SUPPLEMENTAL EXPERIMENTAL PROCEDURES S5: PERFORMANCE EVALUATION OF THE MACHINE LEARNING MODEL

We evaluate the performance of our Gradient Tree Boosting (GTB) model in terms of the R^2-score, which quantifies the proportion of variability explained by our model. A perfect prediction would result in a score of 1, while predicting the mean of the target results in a score of 0. As a benchmark, we compare the GTB model to the daily profile, which is an important null model for frequency dynamics. In particular, we quantify the gain over the daily profile, which

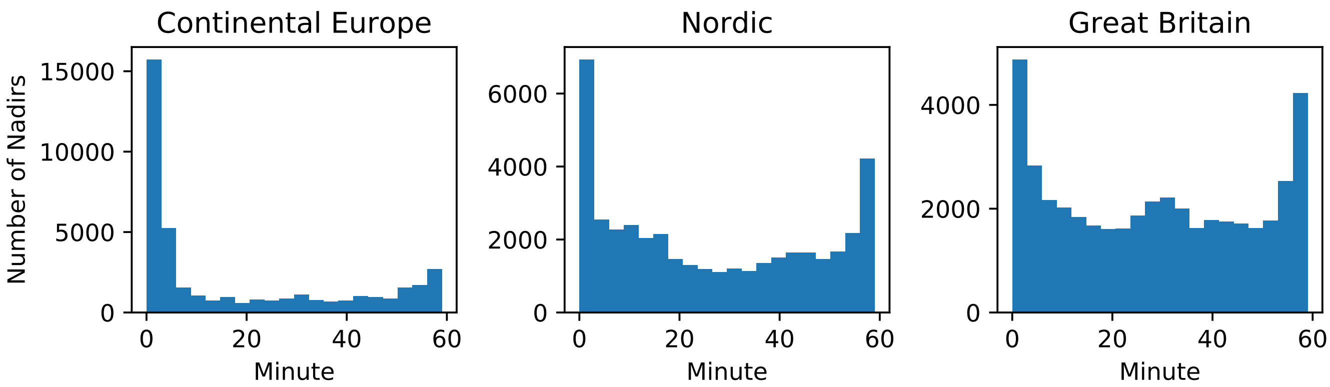

Figure S7. Distribution of Nadir occurrence time. The occurrence time of the nadir is the minute within the hour where the absolute frequency deviation reaches its peak. Its distribution within the hour varies between the grid areas, thus indicating the different importances of DFDs for the grid frequency dynamics.

is the model performance divided by the daily profile performance. Finally, we examine the importance of area-wide feature aggregation and the possibility to predict stability indicators day-ahead. Note that the GTB model used in our main text is referred to as the *full model*. It builds on area-wide aggregated features containing both day-ahead *and* ex-post available data.

The GTB model performs best in Continental Europe, while the performance gain over the daily profile is largest in Great Britain. Figure S8 displays the R^2-score for each stability indicator and each area. We obtain the best predictions in Continental Europe ($R^2 \sim 0.7$) and the lowest scores in Great Britain. In contrast, the performance gain over the daily profile is largest in Great Britain (maximum 16.2) and smallest in Continental Europe (maximum 3.4), while the Nordic area is in between (maximum 7.6). Frequency dynamics in Continental Europe are rather deterministic compared to the stochastic dynamics in Great Britain. Therefore, the prediction is easier and the additional gain through Machine Learning is smaller in Continental Europe. Consistently, the GTB performance is best for the RoCoF as this indicator most strongly reflects the hourly deterministic frequency jumps.

The model performance depends on whether we choose area-wide aggregated features or country-level data (Figure S8). In the Nordic area, we obtain a lower performance when using data from only the largest country (Sweden) instead of aggregating it area-wide. In Continental Europe, the largest country model (using Germany) performs similar to or worse than the aggregated model, and choosing a smaller country (Switzerland) reduces the performance even further. As the grid frequency is affected by all locations within the grid, it is not surprising that data aggregation is important. Overall, the area-wide feature aggregation yields better results than regional data among the areas.

Using only day-ahead available data in our GTB model already outperforms the daily profile for all stability indicators and areas (Figure S9). In Great Britain, the day-ahead model exhibits the strongest performance gain over the daily profile (maximum 8.9), followed by the Nordic area (maximum 3.0) and Continental Europe (maximum 2.6). However, adding ex-post data in the full model can strongly improve the performance, especially in the Nordic area. We quantify this effect in terms of the gain over the day-ahead model, i.e., the full model performance divided by the day-ahead model performance. In the Nordic area, the gain of the full model over the day-ahead model is the largest (maximum 2.6), while it is lowest in Continental Europe (maximum 1.4). The benefits of adding ex-post data in the Nordic area stems from the importance of forecasting errors for the prediction (see main text).

SUPPLEMENTAL EXPERIMENTAL PROCEDURES S6: ADDITIONAL RESULTS WITH SHAP VALUES

We use SHAP values to explain our Machine Learning model for frequency stability indicators. An overview of the most important SHAP dependencies in our model is available in Figures S10, S11, S12 and S13 for each of the four stability indicators. In many cases, we observe non-linear dependencies, which underlines the importance of using a non-linear, complex Machine Learning model such as Gradient Tree Boosting.

Here, we further discuss the effect of synchronous generation on frequency stability indicators. The (total) synchronous generation, which we use as a proxy for the total inertia within the power grid, is not among the eight most important features (Figures S10, S11, S12 and S13). Its feature importance is ranked on places between 19 and 64 in CE, between 38 and 52 in the Nordic area and between 25 and 40 in GB. Overall, the average effect of the (approxi-

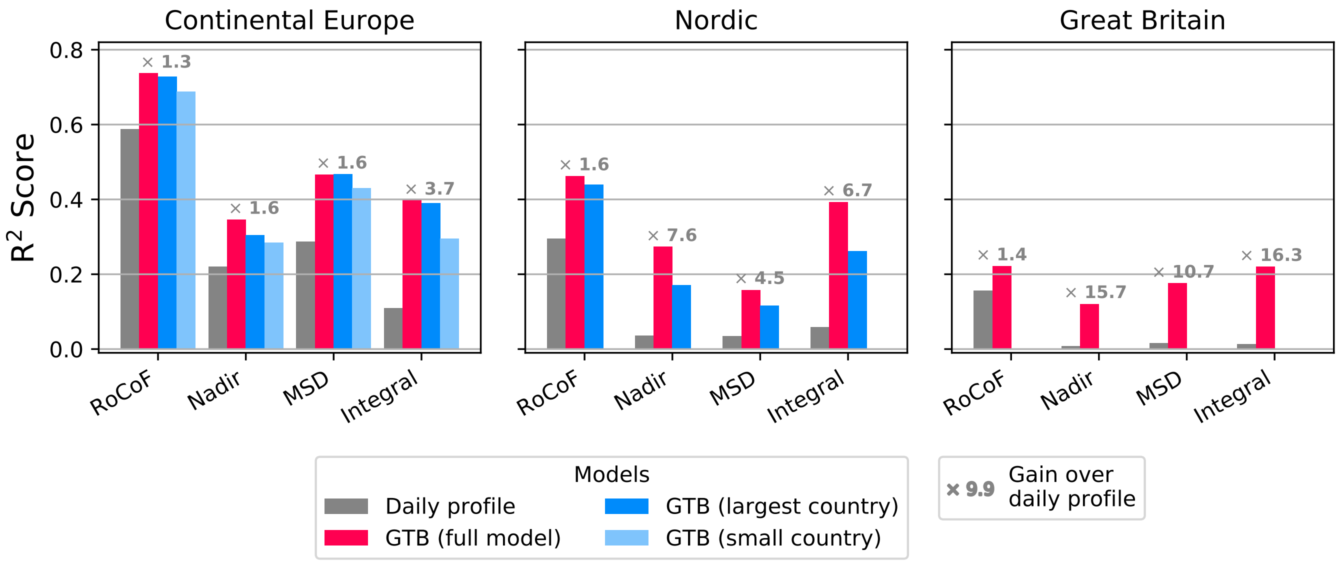

Figure S8. Performance of the full GTB model. The numbers on the bar plots indicate the gain of the GTB model over the daily profile. The full model comprises day-ahead and ex-post available features, which are aggregated area-wide. The country-level models use features from the largest country, i.e., the country with the largest power demand. In Continental Europe, we additionally introduce a model using data from a small country (with smaller average load), which is Switzerland. We retrieve the country-level data from the ENTSO-E transparency platform[1] and construct the same features as in the full model (Table S1).

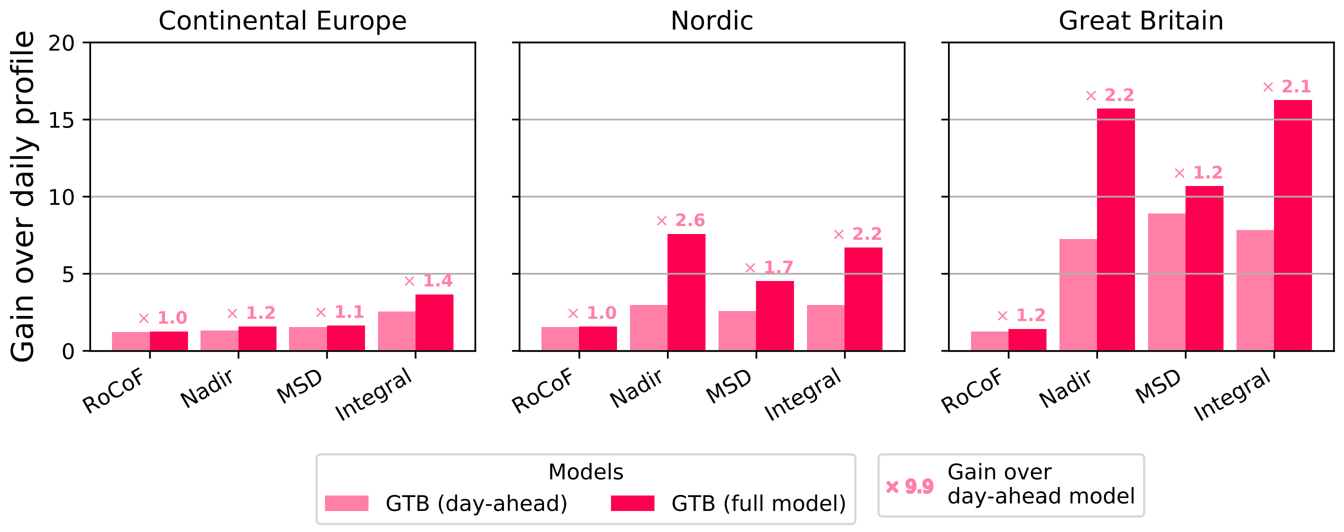

Figure S9. Performance of the day-ahead GTB model. We compare the full model and the day-ahead model in terms of their gain over the daily profile. The numbers on the bar plots indicate the gain of the full model over the day-ahead model.

mated) inertia on the aggregated stability indicators is thus relatively low compared to the most important features. Among the areas, the total synchronous generation is most important in Great Britain, as the feature is constantly among the 40 most important variables. This is consistent with the high share of renewable energy sources in the British power system and the resulting low-inertia situations.[3] The effect of the inertia in Great Britain is depicted in the dependency plots of Figure S14. For all stability indicators, we observe the maximum (absolute) effect of the synchronous generation at low feature values. This is particularly evident for the MSD and the Integral, where the effect of values larger than 20 GW is near to zero. In conclusion, the (approximated) inertia mostly affects frequency stability in Great Britain in extreme situations of low inertia, but the average effect of this feature on our aggregated stability indicators is negligible.

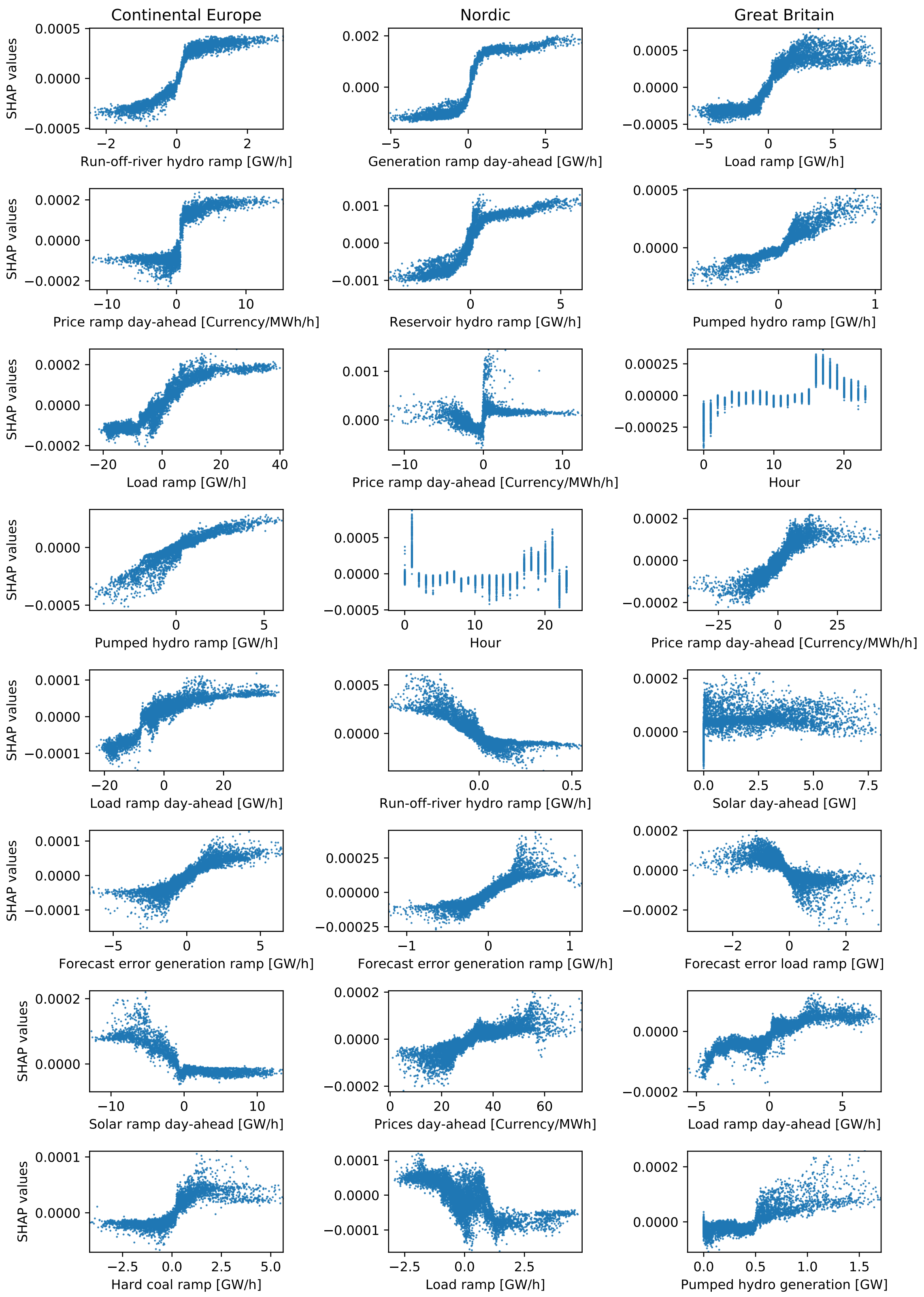

Figure S10. RoCoF dependency plots for the eight most important features.

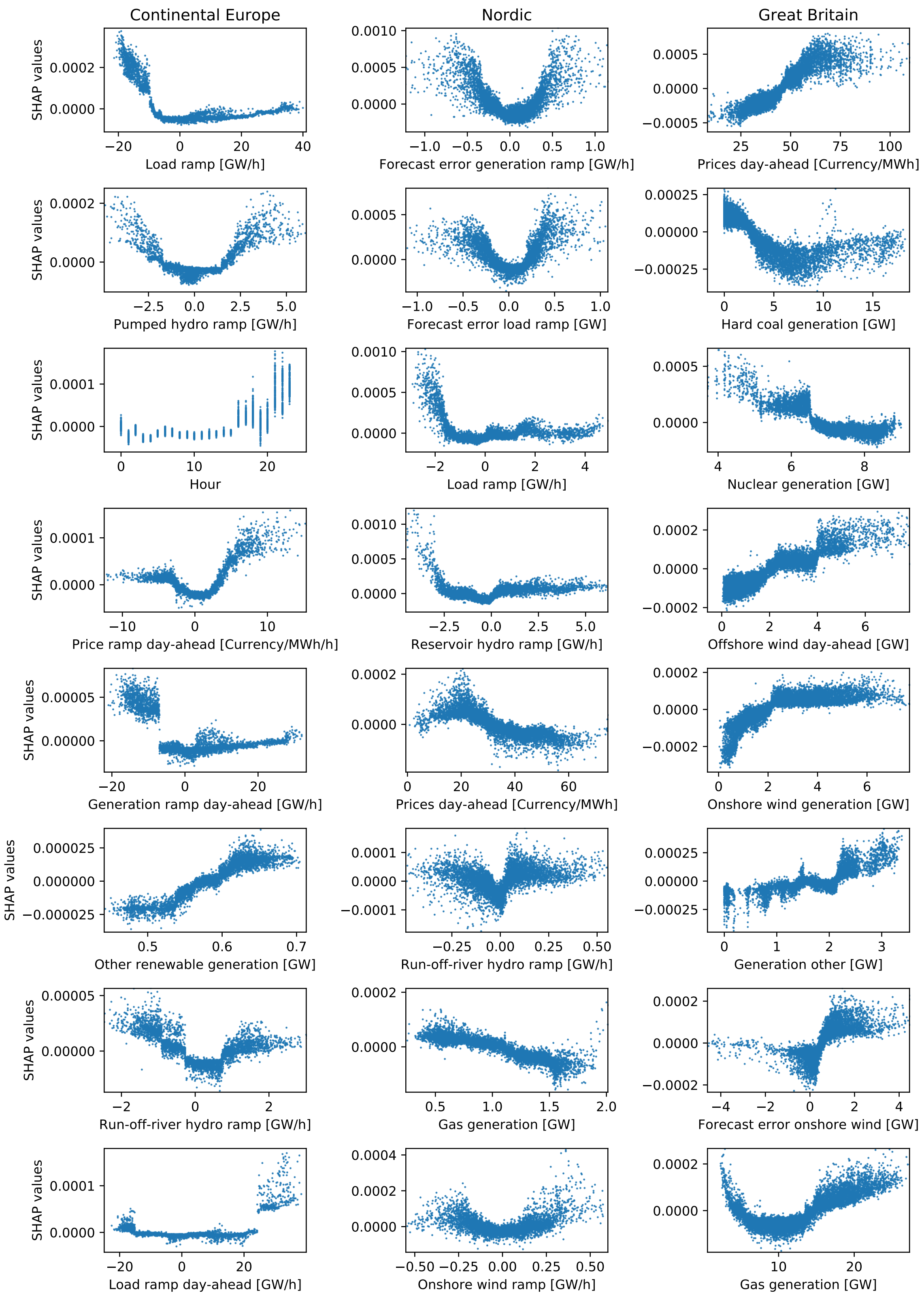

Figure S11. MSD dependency plots for the eight most important features.

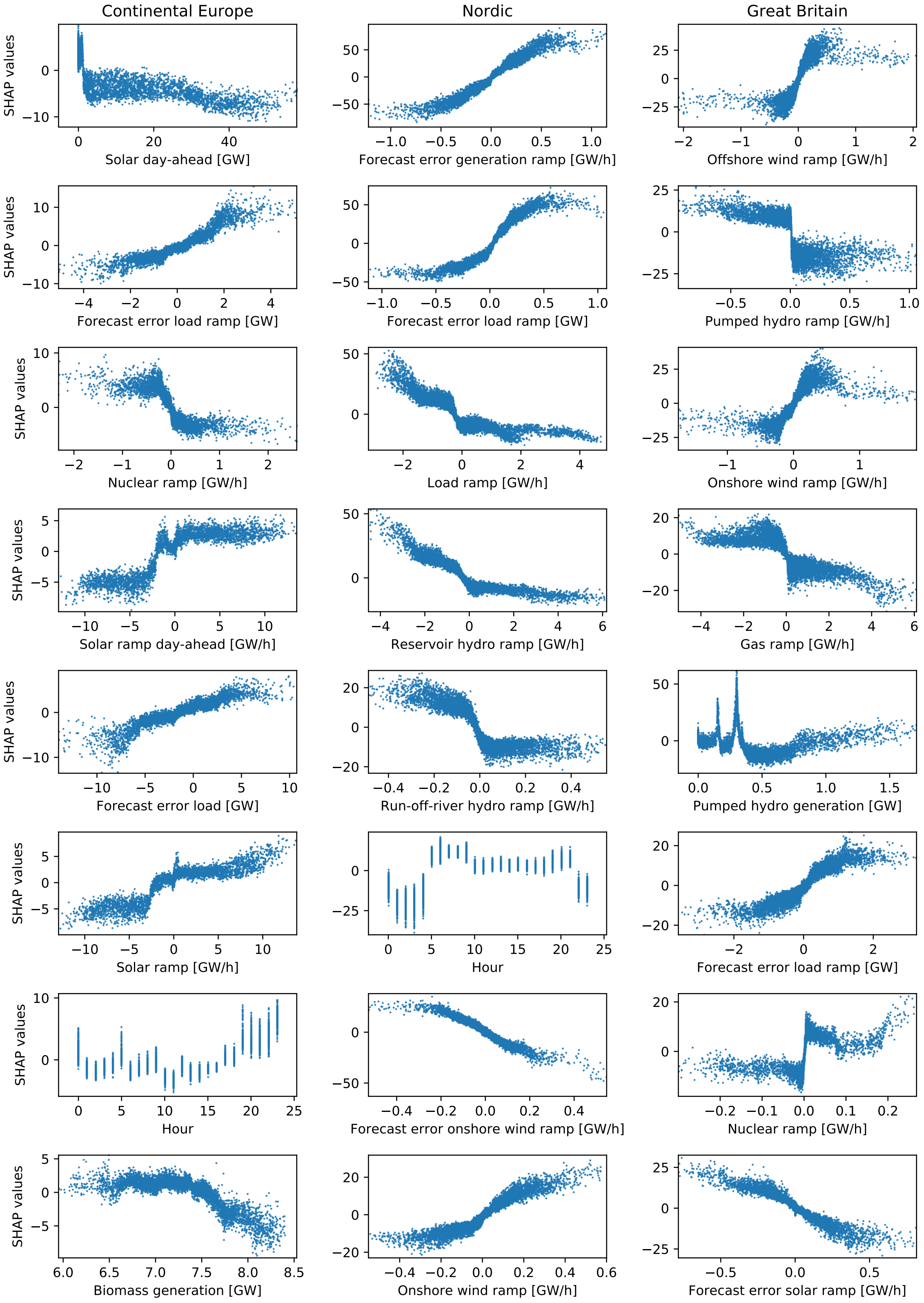

Figure S12. Nadir dependency plots for the eight most important features.

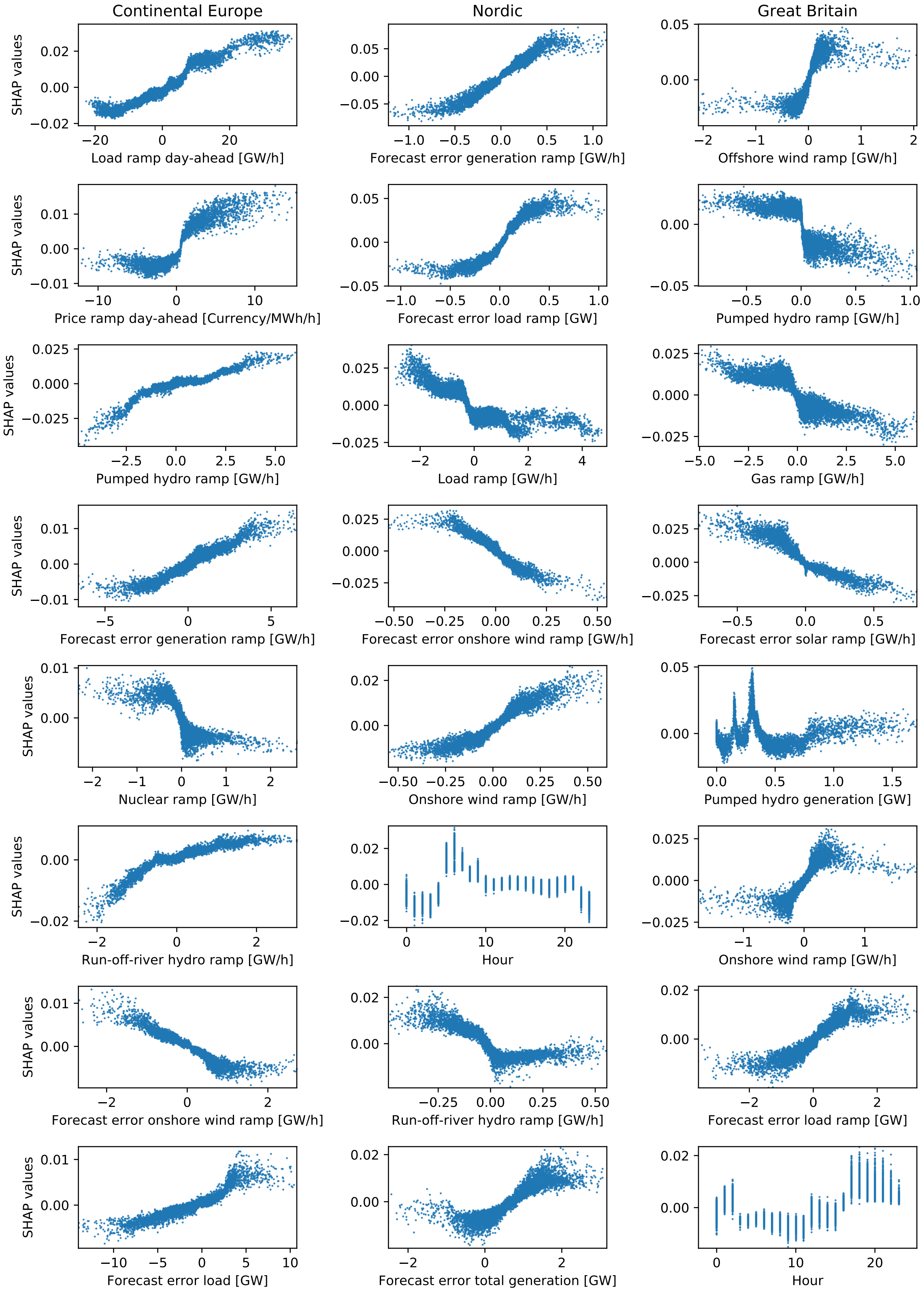

Figure S13. Integral dependency plots for the eight most important features.

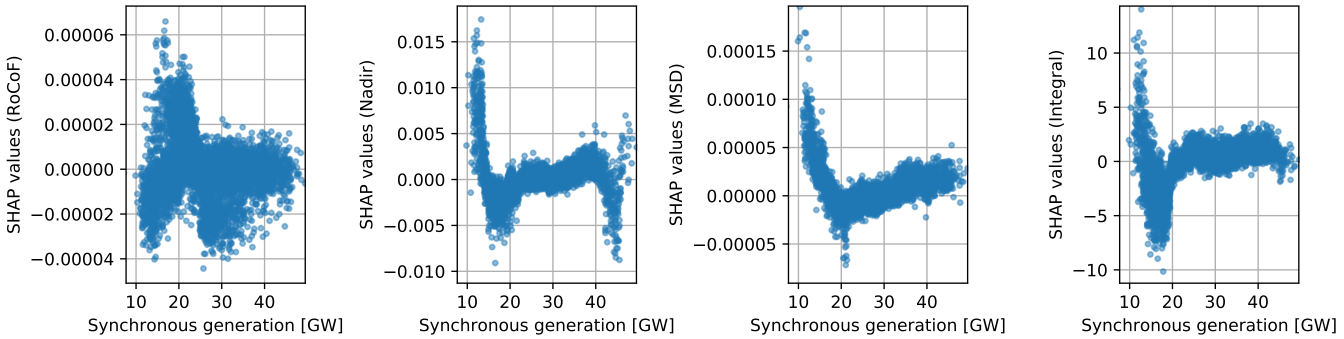

Figure S14. Dependency plots of synchronous generation in Great Britain. Each subplot corresponds to one of our aggregated indicators for frequency stability.

SUPPLEMENTAL REFERENCES

[1] *ENTSO-E Transparency Platform* 2020. https://transparency.entsoe.eu/.

[2] Kruse, J., Schäfer, B. and Witthaut, D. 2021. Supplementary data: "Revealing drivers and risks for power grid frequency stability with explainable AI", https://zenodo.org/record/5118352.

[3] Milano, F., Dörfler, F., Hug, G., Hill, D. J. and Verbič, G. 2018. Foundations and Challenges of Low-Inertia Systems (Invited Paper), *2018 Power Systems Computation Conference (PSCC)*, IEEE, Dublin, pp. 1–25.

[4] Weissbach, T. and Welfonder, E. 2009. High frequency deviations within the European Power System: Origins and proposals for improvement, *2009 IEEE/PES Power Systems Conference and Exposition*, IEEE, Seattle, pp. 1–6.
